# Reduced volume of neuromodulatory brainstem nuclei is associated with early hallucination-related vulnerability in Parkinson’s disease

**DOI:** 10.1093/braincomms/fcag357

**Published:** 2026-09-20

**Authors:** Gabriella Monopoli, Federica Tomaiuolo, Carlo Sestieri, Miriam Punzi, Simone Marino, Francesco Gambi, Mauro Gianni Perrucci, Antonio Ferretti, Stefano L Sensi, Stefano Delli Pizzi

**Affiliations:** Institute for Advanced Biomedical Technologies (ITAB), University ‘G. d'Annunzio’ of Chieti-Pescara, Chieti 66100, Italy; Department of Neuroscience, Imaging, and Clinical Sciences, University ‘G. d'Annunzio’ of Chieti-Pescara, Chieti 66100, Italy; Institute for Advanced Biomedical Technologies (ITAB), University ‘G. d'Annunzio’ of Chieti-Pescara, Chieti 66100, Italy; Department of Engineering and Geology, University ‘G. d'Annunzio’ of Chieti-Pescara, Chieti 66100, Italy; Institute for Advanced Biomedical Technologies (ITAB), University ‘G. d'Annunzio’ of Chieti-Pescara, Chieti 66100, Italy; Department of Neuroscience, Imaging, and Clinical Sciences, University ‘G. d'Annunzio’ of Chieti-Pescara, Chieti 66100, Italy; Institute for Advanced Biomedical Technologies (ITAB), University ‘G. d'Annunzio’ of Chieti-Pescara, Chieti 66100, Italy; Department of Neuroscience, Imaging, and Clinical Sciences, University ‘G. d'Annunzio’ of Chieti-Pescara, Chieti 66100, Italy; Institute for Advanced Biomedical Technologies (ITAB), University ‘G. d'Annunzio’ of Chieti-Pescara, Chieti 66100, Italy; Department of Neuroscience, Imaging, and Clinical Sciences, University ‘G. d'Annunzio’ of Chieti-Pescara, Chieti 66100, Italy; Department of Neuroscience, Imaging, and Clinical Sciences, University ‘G. d'Annunzio’ of Chieti-Pescara, Chieti 66100, Italy; Molecular Neurology Unit, Center for Advanced Studies and Technology (CAST), University ‘G. d'Annunzio’ of Chieti-Pescara, Chieti 66100, Italy; Institute for Advanced Biomedical Technologies (ITAB), University ‘G. d'Annunzio’ of Chieti-Pescara, Chieti 66100, Italy; Department of Neuroscience, Imaging, and Clinical Sciences, University ‘G. d'Annunzio’ of Chieti-Pescara, Chieti 66100, Italy; UdA-TechLab, Research Center, University ‘G. d'Annunzio’ of Chieti-Pescara, Chieti 66100, Italy; Institute for Advanced Biomedical Technologies (ITAB), University ‘G. d'Annunzio’ of Chieti-Pescara, Chieti 66100, Italy; Department of Neuroscience, Imaging, and Clinical Sciences, University ‘G. d'Annunzio’ of Chieti-Pescara, Chieti 66100, Italy; UdA-TechLab, Research Center, University ‘G. d'Annunzio’ of Chieti-Pescara, Chieti 66100, Italy; Institute for Advanced Biomedical Technologies (ITAB), University ‘G. d'Annunzio’ of Chieti-Pescara, Chieti 66100, Italy; Department of Neuroscience, Imaging, and Clinical Sciences, University ‘G. d'Annunzio’ of Chieti-Pescara, Chieti 66100, Italy; Molecular Neurology Unit, Center for Advanced Studies and Technology (CAST), University ‘G. d'Annunzio’ of Chieti-Pescara, Chieti 66100, Italy; Neurology Institute, SS Annunziata University Hospital, University ‘G. d'Annunzio’ of Chieti-Pescara, Chieti 66100, Italy; Institute for Advanced Biomedical Technologies (ITAB), University ‘G. d'Annunzio’ of Chieti-Pescara, Chieti 66100, Italy; Department of Neuroscience, Imaging, and Clinical Sciences, University ‘G. d'Annunzio’ of Chieti-Pescara, Chieti 66100, Italy; Molecular Neurology Unit, Center for Advanced Studies and Technology (CAST), University ‘G. d'Annunzio’ of Chieti-Pescara, Chieti 66100, Italy

**Keywords:** brainstem, Parkinson’s disease, laterodorsal tegmental nucleus, locus coeruleus, visual hallucinations

## Abstract

Early hallucination-related phenomena, including minor visual hallucinations (mVHs), are clinically relevant features of Parkinson’s disease (PD), but their neuroanatomical substrates remain unclear. Brainstem nuclei supporting sensory gating, together with cholinergic and noradrenergic dysregulation, are proposed modulators of hallucinations. The objective of the present study was to test whether PD participants with early hallucination-related phenomena show reduced volumes of brainstem nuclei with cholinergic and noradrenergic relevance, using cross-sectional analysis of baseline structural MRI, clinical, and neuropsychological data from 641 PD participants in the Parkinson’s Progression Markers Initiative (PPMI). Participants scoring ≥1 at screening, baseline, or during the first nine months of follow-up were categorized as PD–mVHs^+^, whereas those who consistently scored 0 throughout the first year were categorized as PD–mVHs^−^. Histology-informed probabilistic atlases were used to segment brainstem, subcortical, and cortical regions. Analyses of covariance (ANCOVAs) compared regional volumes across groups, with group and sex as fixed factors, the group × sex interaction, and age, education, standardized estimated intracranial volume (eTIV), Montreal Cognitive Assessment (MoCA) score, and disease duration as continuous covariates. Hierarchical logistic regression tested whether brainstem volumes added explanatory value for hallucination-related status beyond clinical, neuropsychological, and other volumetric predictors. PD-mVHs^+^ participants showed reduced left locus coeruleus (LC) and left laterodorsal tegmental nucleus (LDTg) volumes, with no significant differences in pedunculopontine tegmental nucleus or dorsal raphe nuclei. After covariate adjustment, the left LDTg effect survived both Bonferroni and false discovery rate (FDR) correction, whereas the left LC effect survived FDR correction but not the Bonferroni-adjusted threshold. Adding LC and LDTg volumes to the hierarchical logistic regression improved fit beyond disease duration, depressive symptoms, immediate verbal recall, and semantic fluency. These findings suggest that reduced MRI-derived volume of the left LDTg, and possibly of the left LC, is associated with early hallucination-related vulnerability in PD.

## Graphical Abstract

**Graphical Abstract:**
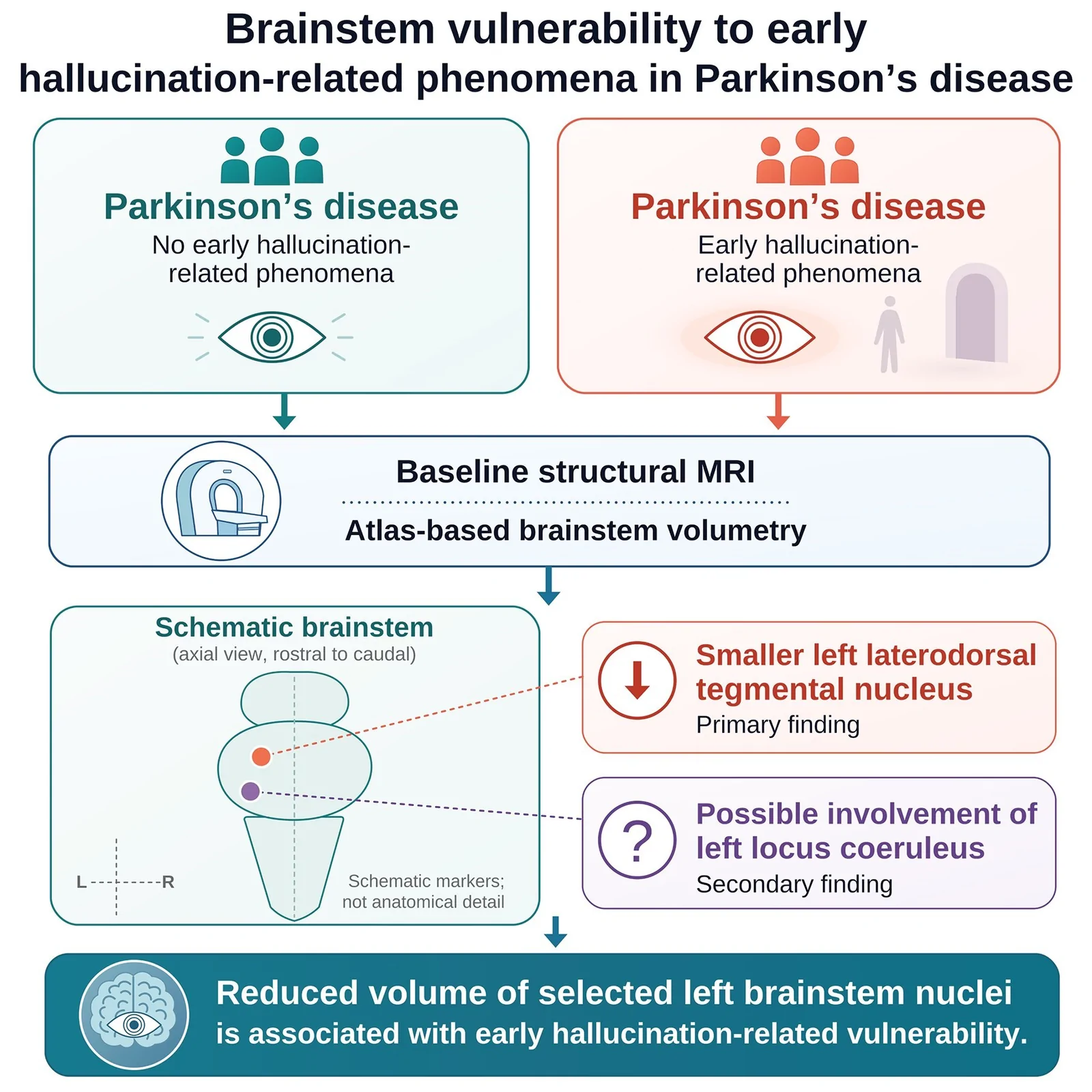

## Teaser

Monopoli *et al*. demonstrate that Parkinson’s disease patients with early hallucination-related phenomena show reduced MRI-derived volume of neuromodulatory brainstem nuclei, most robustly the left laterodorsal tegmental nucleus. These associations, together with clinical and neuropsychological factors, may contribute to vulnerability to early perceptual disturbances.

## Introduction

Parkinson’s disease (PD) is a progressive neurodegenerative disorder that affects ∼1% of individuals over 60 years of age.^1-3^ Although originally characterized by motor symptoms such as tremor, rigidity, bradykinesia, and postural instability, PD is now recognized as a multisystem disorder in which non-motor features play a major role in shaping quality of life and prognosis.^4-6^ Among these non-motor symptoms, visual hallucinations (VHs) are particularly common and clinically relevant, as they are associated with functional decline, caregiver distress, institutionalization, and increased mortality.^7,8^ Up to 70% of patients with advanced disease may develop VHs,^9,10^ yet their neurobiological basis remains incompletely understood. A clearer understanding of the anatomical and neurobiological correlates of early hallucination-related phenomena may improve risk stratification and inform models of disease progression.

VHs in PD are heterogeneous and span a broad clinical spectrum. A widely adopted classification separates complex visual hallucinations (cVHs) from minor visual hallucinations (mVHs) based on their phenomenology and presumed substrates.^8,9,11^ CVHs consist of vivid, detailed percepts of people, animals, or scenes, frequently endowed with emotional salience and typically experienced as real.^7,9,10^ By contrast, mVHs comprise subtler perceptual phenomena such as presence or passage hallucinations, fleeting peripheral images, shadows, flashes, or misperceptions of objects.^7,8,10^ MVHs often precede cVHs by several years, suggesting shared pathophysiological mechanisms.

Neuroimaging studies have provided important insights into the neural correlates of VHs. Structural MRI analyses using voxel-based morphometry and cortical thickness analysis in patients with cVHs have often identified abnormalities in posterior cortical regions, particularly the occipital and parietal cortices,^10,12-16^ together with involvement of limbic and thalamic regions.^17,18^ Similar posterior cortical and pulvinar alterations have been reported in dementia with Lewy bodies (DLB), supporting a more general contribution of posterior visual and thalamic networks to hallucination phenomenology.^19,20^ In contrast, the neural substrates of mVHs are less firmly established: seminal studies have reported posterior cortical and cerebellar involvement, as well as relative volumetric increases in paralimbic structures.^21-23^ Overall, the anatomical correlates of early hallucination-related phenomena compatible with mVHs remain unclear.

Beyond cortical and higher-order subcortical structures, converging evidence implicates brainstem nuclei in the vulnerability to hallucinations, given their roles in arousal regulation, sensory gating, and neuromodulatory balance.^24^ Distinct neurotransmitter systems may contribute differently to different hallucinatory phenomena. CVHs have been consistently linked to dopaminergic signaling^25,26^ and serotonergic dysfunction, particularly via 5-HT_2A_ receptors.^4,13,27,28^ In contrast, the occurrence of mVHs in untreated PD patients suggests that these experiences cannot be solely attributed to dopaminergic therapy.^7,9^ Cholinergic and noradrenergic dysfunction has been proposed as a key contributor to VHs,^29^ although direct in vivo evidence remains scarce. Clinically, exposure to anticholinergic drugs increases the risk of hallucination,^9^ and both imaging and post-mortem studies demonstrate widespread cholinergic denervation in PD.^30,31^ Atrophy-compatible structural changes in the pedunculopontine nucleus and thalamus have also been associated with VHs.^32^ Finally, converging findings implicate the noradrenergic system: through its extensive projections to cortical and subcortical regions, the locus coeruleus (LC) regulates arousal and perceptual gain.^33-37^ LC degeneration occurs early in PD^38,39^ and has been linked to hallucinations in both PD and DLB,^40,41^ while experimental LC activation enhances visual discrimination.^42^

Despite their relevance, these structures have remained largely underexplored, as conventional MRI has limited the anatomical precision needed to reliably identify small brainstem nuclei in vivo. Recent advances in probabilistic, histology-informed atlases partially address this limitation by enabling atlas-informed volumetric estimation of structures that are otherwise difficult to delineate in vivo. In particular, the combination of the Harvard Ascending Arousal Network (AAN) atlas^43^ and the HistoAtlas integrated within the NextBrain framework^44^ provides improved cytoarchitectonic resolution and probabilistic labelling across the entire brain. Such volumetry remains an indirect structural proxy rather than direct evidence of neurotransmitter-specific degeneration; nonetheless, these methodological advances offer an opportunity to test whether atlas-derived volumetric differences in specific brainstem nuclei are associated with hallucination-related vulnerability in PD, using large-scale clinical MRI datasets.

In the present study, we investigated whether PD patients who experienced early hallucination-related phenomena compatible with mVHs (PD-mVHs^+^) differ from those without such phenomena (PD-mVHs^−^) in the MRI-derived volumes of key cholinergic and noradrenergic brainstem nuclei, including the pedunculopontine tegmental nucleus (PPTg), the laterodorsal tegmental nucleus (LDTg), and the locus coeruleus (LC). To this end, we leveraged data from the Parkinson’s Progression Markers Initiative (PPMI),^45^ a large, multicentre cohort that provides harmonized clinical, neuropsychological, and neuroimaging assessments. Because PPMI does not include a dedicated phenomenological assessment of mVHs, hallucination-related status was defined using MDS-UPDRS Part I item 1.2 assessments obtained at screening, baseline, and during early follow-up. We focused on hallucination-related phenomena occurring around baseline or during the early follow-up period, while acknowledging that PD treatment at baseline and/or during follow-up may represent a potential confounder.

Volumetric quantification of brainstem nuclei was performed using advanced segmentation pipelines incorporating the AAN atlas-based segmentation.^46^ We first conducted a direct region-of-interest analysis aimed at detecting significant volumetric differences across groups, controlling for demographic variables and general cognitive decline. We then conducted hierarchical logistic regression models including clinical, neuropsychological, and additional volumetric predictors identified from exploratory analyses, to assess the incremental association of brainstem volumetric differences with hallucination-related status.

## Materials and methods

### Ethics

The research protocol adhered to the principles outlined in the Declaration of Helsinki. Written informed consent was obtained from all individuals before participation. Ethics approval was secured from the institutional review boards of each contributing centre. Ethical documentation is available at www.ppmi-info.org/data.

### Study cohort and participant selection

Data used in the preparation of this article were obtained from the Parkinson’s Progression Markers Initiative (PPMI) database (https://www.ppmi-info.org/access-data-specimens/download-data), RRID:SCR_006431. For up-to-date information on the study, visit http://www.ppmi-info.org. The PPMI is a publicly available, longitudinal, observational, multi-site cohort designed to characterize the progression of PD and support biomarker development. The present study used baseline structural MRI scans in a cross-sectional imaging analysis, while hallucination-related status was defined using MDS-UPDRS Part I item 1.2 assessments obtained at screening, baseline, and during early follow-up. The October 2023 data release was used, comprising harmonized clinical, neuroimaging, and cognitive assessments. A total of 641 individuals with a clinical diagnosis of PD were included. Diagnosis was made according to standardized criteria requiring asymmetric bradykinesia or tremor, or at least 2 cardinal motor features (bradykinesia, resting tremor, rigidity), and was confirmed by dopamine transporter (DaT) SPECT positivity.

Demographic information included age, sex, education, age at PD diagnosis, and disease duration. Motor severity was assessed using the Movement Disorder Society—Unified Parkinson’s Disease Rating Scale Part III (MDS-UPDRS III) and Hoehn and Yahr (H&Y) staging. Non-motor features were measured with validated tools: depressive symptoms using the 15-item Geriatric Depression Scale (GDS-15), and anxiety using the State-Trait Anxiety Inventory (STAI). Clinical histories were reviewed to exclude comorbidities associated with primary psychotic disorders, dementia, or criteria consistent with DLB. Full inclusion and exclusion criteria are available at www.ppmi-info.org/study-design.

Participants were classified according to early hallucination-related status using assessments obtained at screening, baseline, and during the first year of follow-up. Because the PPMI dataset does not include a dedicated phenomenological instrument specifically designed to distinguish mVHs from other hallucination-related experiences, we used item 1.2 (‘Hallucinations and Psychosis’) of the MDS-UPDRS Part I as a proxy measure. Participants scoring ≥1 at screening, baseline, or during the first nine months of follow-up were categorized as PD–mVHs^+^, whereas those who consistently scored 0 throughout the first year were categorized as PD–mVHs^−^. This asymmetric classification rule was chosen to capture hallucination-related phenomena occurring around baseline and during the early follow-up period, while the PD–mVHs^−^ group was conservatively defined by the absence of hallucination-related symptoms across the full first-year observation window. To ensure classification reliability, only individuals with at least 12 months of validated follow-up were retained, and cases with uncertain symptom onset were excluded. Therefore, the PD-mVHs^+^ group should be interpreted as representing patients with early hallucination-related phenomena compatible with mVHs, rather than a group defined by a dedicated mVH-specific assessment.

PD treatment represents a potential confounder, as a subset of participants was already receiving treatment for PD symptoms at baseline and additional participants may have initiated dopaminergic therapy during follow-up. Because hallucination-related status was defined using assessments obtained at screening, baseline, and during early follow-up, treatment exposure may have contributed to the presence or reporting of hallucination-related symptoms in some PD–mVHs^+^ participants. Detailed medication exposure, treatment timing, drug class, and levodopa equivalent daily dose at the time of hallucination-related assessment could not be consistently determined; thus, potential treatment-related effects cannot be excluded. Accordingly, the present findings should be interpreted as baseline structural correlates of early hallucination-related status, rather than as definitive evidence of drug-independent mVHs. Healthy controls were not included in inferential analyses due to the lack of longitudinal clinical and psychosis assessments.^47^

### Cognitive assessment

Baseline cognitive functioning was evaluated using a standardized neuropsychological battery encompassing domains typically affected in PD. Global cognition was assessed using the Montreal Cognitive Assessment (MoCA). Verbal learning and memory performance was measured using the Hopkins Verbal Learning Test–Revised, which includes immediate and delayed recall and recognition trials. Language function was evaluated using the Modified Boston Naming Test, semantic fluency (animal naming), and phonemic fluency (F-A-S task). Processing speed and attention were measured with the Symbol Digit Modalities Test (SDM-TOT) and Trail Making Test Part A (TMT-A), both sensitive to subcortical dysfunction commonly seen in PD. Visuospatial abilities were assessed using the Benton Judgment of Line Orientation (JLO) and the Clock Drawing Test, both widely accepted measures of visuospatial processing in PD populations. Executive functioning was examined using the Letter-Number Sequencing (LNS) subtest from the WAIS-III, which targets working memory and mental manipulation, and the Trail Making Test Part B (TMT-B), which assesses cognitive flexibility and set-shifting ability.

### MRI data acquisition

High-resolution structural MRI data were acquired using a sagittal 3D T1-weighted sequence (e.g. MP-RAGE or IR-FSPGR). Imaging parameters included a slice thickness of 1.0 mm (expandable to 1.2 mm for full brain coverage), 192 contiguous slices per volume, and an in-plane resolution of 1.0 × 1.0 mm^2^. The field of view was 256 mm, with a 256 × 256 acquisition matrix, no zero filling or interpolation, and anterior-to-posterior phase encoding. Total scan duration was ∼7 min. Parallel imaging techniques (GRAPPA for Siemens systems; ASSET for GE systems) were employed to optimize acquisition time, consistent with vendor guidelines. Imaging protocols were harmonized across all scanning sites, with allowance for minor scanner-specific adjustments where necessary.

### MRI data analysis

We adopted a multi-atlas morphometric approach integrating FreeSurfer’s recon-all pipeline,^48^ the AAN atlas,^46^ and the HistoAtlas, a histology-based probabilistic atlas incorporated into the NextBrain platform (Fig. 1).^44^ MRI preprocessing, analysis, and segmentation were performed using FreeSurfer v8.0.0 (https://surfer.nmr.mgh.harvard.edu/), which includes the deep learning-based SynthSeg algorithm.^49^ The combined use of the AAN and HistoAtlas enabled cytoarchitectonically informed, atlas-based volumetric estimation of brainstem nuclei as well as cortical and subcortical regions. This approach provided probabilistic labels for structures that are difficult to delineate with conventional T1-weighted MRI, although we acknowledge that segmentation of very small brainstem nuclei remains primarily atlas-informed rather than directly contrast-driven.

**Figure 1:**
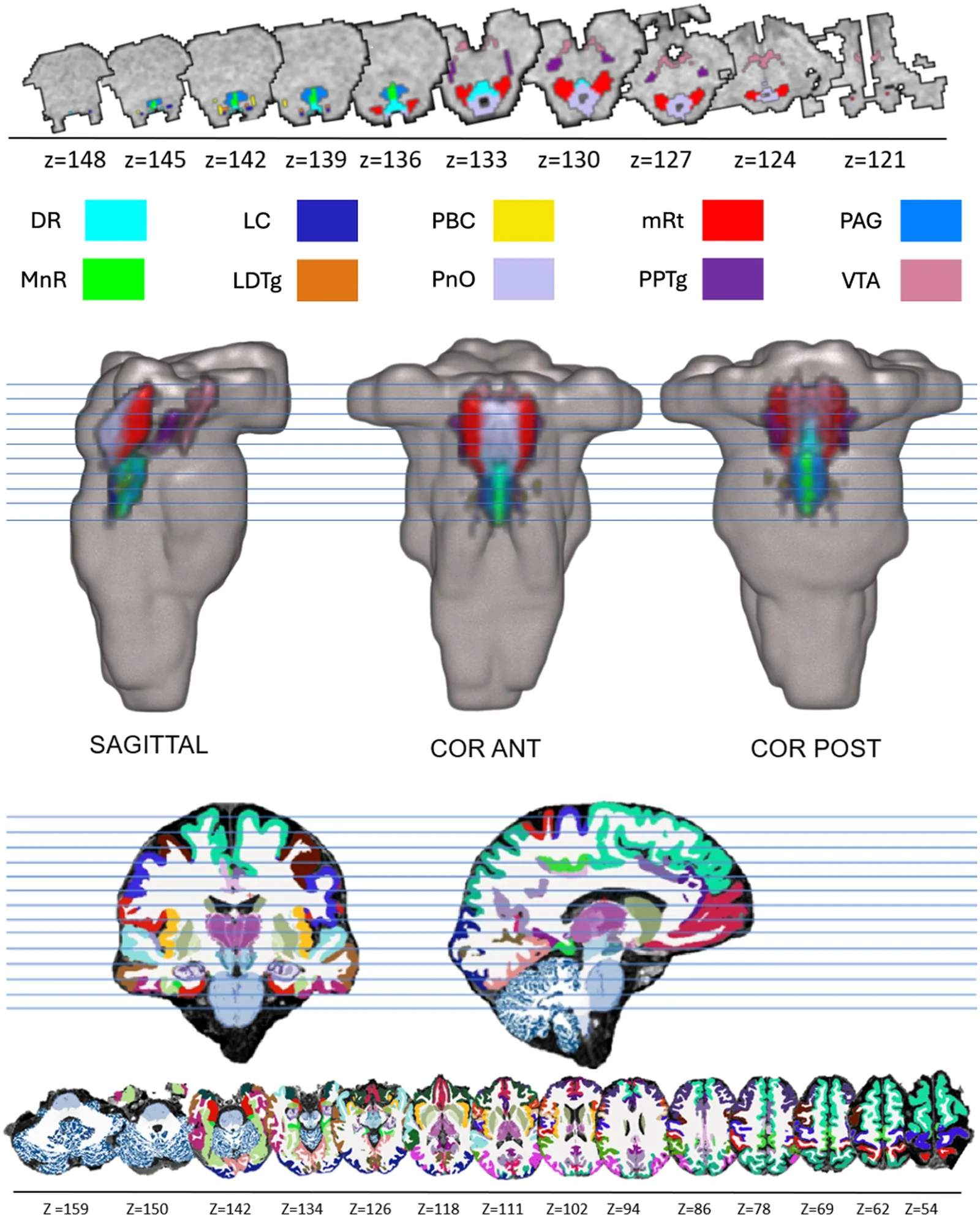
**Brainstem and whole-brain segmentation using histology-informed probabilistic atlases.** The upper panel shows serial axial brainstem sections along the rostrocaudal axis, with colour-coded overlays of nuclei derived from the AAN atlas: DR, median raphe (MnR), LC, LDTg, PBC, nucleus pontis oralis (PnO), mesencephalic reticular formation (mRt), PPTg, PAG, and ventral tegmental area (VTA). The labelled colour key identifies each brainstem region; colours correspond to the original FreeSurfer lookup tables associated with the atlas. The middle panel shows three-dimensional renderings of the brainstem in sagittal, anterior coronal (COR ANT), and posterior coronal (COR POST) views. Horizontal blue lines indicate the levels corresponding to the displayed axial sections. The lower panel shows representative coronal and sagittal views and serial axial sections of whole-brain parcellations derived from HistoAtlas within the NextBrain framework, providing cytoarchitectonically informed segmentation of cortical, subcortical, cerebellar, and brainstem regions. Together, these atlases enabled volumetric assessment of small brainstem nuclei and whole-brain morphometry in vivo.

Within this framework, the AAN atlas provided probabilistic volumetric estimates for the nuclei central to our hypotheses, including the LC, LDTg, PPTg, and dorsal raphe (DR). The approach also quantified additional brainstem regions implicated in arousal, sensory integration, and neuromodulation, such as the median raphe nucleus (MnR), which is rich in serotonergic neurons and has limbic projections involved in mood, memory, and circadian regulation^50^; the mesencephalic reticular formation (mRt), associated with wakefulness and sensorimotor processing; the ventral tegmental area (VTA), the principal dopaminergic source of mesolimbic and mesocortical pathways^51,52^; the pontis oralis (PnO), involved in REM sleep generation^53^; the parabrachial complex (PBC), a key node for visceral integration and autonomic control^54^; and the periaqueductal grey (PAG), critical for pain modulation and defensive behaviours.^55,56^

The HistoAtlas further enabled volumetric assessment of additional structures, including hippocampal subfields, amygdalar nuclei, thalamic subregions, hypothalamic areas, mammillary bodies, and cortical and cerebellar structures. These regions formed part of the exploratory analysis contextualizing brainstem volumetric differences within broader networks potentially involved in hallucination-related vulnerability. Cerebellar coverage was incomplete and represented a methodological limitation. Within this exploratory set, the claustrum and habenula were of theoretical interest given their documented involvement in mechanisms underlying hallucinations across neurodegenerative and psychiatric conditions.^57,58^ Volumetric measures of the substantia nigra subdivisions (SNc, SNr) were also included, given their relevance to dopaminergic pathology and motor disease asymmetry in PD.

### Statistical models

Only participants with complete demographic, clinical, and MRI data were included in the study. The sole exception was the Trail Making Test, which exhibited a high proportion of missing values. To avoid excluding a large number of otherwise eligible participants, individuals with missing Trail Making Test scores were retained, and this variable was omitted from the regression models.

We first tested for differences in demographic variables (age, sex, years of education), clinical measures (disease duration, MDS-UPDRS Part III, Hoehn and Yahr stage, GDS, STAI), and neuropsychological scores (MoCA, semantic fluency, Hopkins Verbal Learning Test–Revised—HVLT, SDM-TOT, LNS, and JLO) between participants with and without early hallucination-related phenomena compatible with mVHs using independent-samples *t*-tests or χ^2^ tests, as appropriate.

We then tested our a priori hypothesis that hallucination-related status would be associated with MRI-derived volumetric differences in brainstem nuclei with cholinergic, noradrenergic, and serotonergic relevance. The focused analysis included seven a priori nuclei: left and right LC, left and right LDTg, left and right PPTg, and DR. For each nucleus, group differences were examined using ANCOVA. Group and sex were entered as categorical fixed factors, together with the group × sex interaction, whereas age, education, standardized estimated total intracranial volume (eTIV z-score), disease duration, and MoCA score were included as continuous covariates. The reported F statistics correspond to Type III tests of the main effect of group. To control for multiple comparisons across the seven a priori nuclei, a Bonferroni threshold was applied (0.05/7 = 0.007). FDR-adjusted *P*-values were also reported as a complementary correction across the same predefined set of nuclei. Effect sizes for ANCOVA models were reported as partial eta squared (ηp^2^).

To contextualize the hypothesis-driven brainstem findings within broader anatomical networks, we also conducted exploratory regional ANCOVAs across the remaining brainstem, subcortical, cerebellar, and cortical regions. These analyses included the same covariate set. The exploratory analyses were not corrected for multiple comparisons and were used only to identify candidate regions for contextual sensitivity analyses. Regions showing between-group differences at an uncorrected *P* ≤ 0.05 level were considered candidate regions for the exploratory sensitivity analyses. Accordingly, results from these regional screens were considered hypothesis-generating and were not used for primary confirmatory inference.

Hierarchical logistic regression with bootstrap resampling was then used to assess whether the hypothesis-driven LC and LDTg volumes added explanatory value beyond clinical and neuropsychological variables. The aim of these models was not to develop a predictive classifier, but to test the incremental association of a priori brainstem volumes with hallucination-related status. Hallucination-related status (0 = PD-mVHs^−^; 1 = PD-mVHs^+^) was the dependent variable. Before constructing the regression models, demographic, clinical, and neuropsychological variables were screened using between-group comparisons. To limit model complexity, given the relatively small number of PD–mVHs^+^ participants, only variables showing significant between-group differences were entered into the baseline regression model. Accordingly, Block 1 included disease duration, semantic fluency (SF-Animal), GDS score, and HVLT-IR. Block 2 added the hypothesis-driven brainstem predictors identified in the primary volumetric analysis, namely left LC and left LDTg volumes. Block 2 was considered the main hypothesis-driven regression model. For the primary hierarchical logistic regression only, model fit was further compared between Block 1 and Block 2 using the Akaike Information Criterion (AIC), to quantify whether the hypothesis-driven addition of the selected MRI-derived brainstem predictors improved fit while accounting for the increase in model complexity.

As a sensitivity analysis to address the phenomenological breadth of MDS-UPDRS Part I item 1.2, we repeated the focused brainstem ANCOVA after restricting the hallucination-positive group to participants whose maximum item 1.2 score was 1 at screening, baseline, or during the first nine months of follow-up. Participants with scores ≥2 were excluded, whereas the PD-mVHs^−^ group remained defined as participants scoring 0 throughout the first year of follow-up. We also repeated the main Block 2 logistic regression model in this restricted score-1 subgroup to test whether the incremental association of left LC and left LDTg volumes was preserved in participants reporting symptoms most compatible with minor hallucination-related phenomena.

Blocks 3 and 4 were treated as exploratory sensitivity analyses. Block 3 added the candidate regions identified from the disease-duration-adjusted regional ANCOVAs to the Block 2 predictors. Block 4 repeated the same extended model after removing left LC and left LDTg. Importantly, these models were used to examine whether the LC/LDTg associations remained directionally stable when considered alongside other candidate morphometric variables and were not interpreted as providing corrected evidence for the independent contribution of each region identified with the ANCOVAs. Given the limited number of PD–mVHs^+^ cases relative to the number of candidate predictors, the results of Blocks 3 and 4 should be treated with caution, given the risk of overfitting even after bootstrap resampling.

As an additional sensitivity analysis addressing scanner-related variability and disease asymmetry, we repeated the main Block 2 logistic regression model after adding scanner manufacturer and an asymmetry index of substantia nigra pars compacta (SNc) volume [(Left SNc − Right SNc)/(Left SNc + Right SNc)] as covariates. Scanner information was harmonized at the manufacturer level into three categories: GE, Philips, and Siemens, with Siemens used as the reference category because it represented the largest manufacturer group. This sensitivity model was used to test whether the LC/LDTg associations were explained by multi-vendor acquisition effects or by global nigrostriatal asymmetry.

For the standard logistic regression models, we reported regression coefficients (B ± SE), Wald statistics, *P*-values, and odds ratios [Exp(B)]. Model fit was assessed using omnibus *χ*^2^ tests and Cox–Snell and Nagelkerke pseudo-*R*^2^ indices. The robustness of the regression coefficients was evaluated using non-parametric bootstrapping with 5000 resamples, with bias-corrected and accelerated (BCa) 95% confidence intervals reported for B. Bootstrap support for individual coefficients was defined by BCa 95% confidence intervals excluding zero. Bootstrap *P*-values are reported for completeness. Incremental model fit between nested blocks was assessed using likelihood-ratio *χ*^2^ tests based on changes in −2 log likelihood.

## Results

### Demographic and clinical outcomes

Independent-samples *t*-tests revealed clinical and neuropsychological differences between PD–mVHs^+^ and PD–mVHs^−^ participants (Table S1). Compared with PD–mVHs^−^ participants, the PD–mVHs^+^ group had longer disease duration, higher depressive symptom scores on the GDS, slightly higher verbal episodic memory performance as measured by Hopkins Verbal Learning Test–Revised Immediate Recall (HVLT-IR), and lower semantic fluency performance on the animal-naming task. No significant group differences were observed for age, education, global motor severity, Hoehn and Yahr stage, or performance across the other cognitive domains examined. The overall sample included 403 male and 238 female participants, corresponding to a male:female ratio of 1.69:1. Sex distribution did not differ significantly between the PD–mVHs^−^ and PD–mVHs^+^ groups [*χ*^2^(1) = 2.470, *P* = 0.116, *φ* = 0.062].

### Morphometry of noradrenergic, serotonergic, and cholinergic brainstem nuclei

PD-mVHs^+^ participants showed reduced volumes in the left LDTg and left LC compared with PD-mVHs^−^ participants (Table 1). The left LDTg effect survived Bonferroni correction across the seven predefined brainstem nuclei (*F* = 7.955, *P* = 0.005, pFDR = 0.030, ηp^2^ = 0.012), whereas the left LC effect remained significant after FDR correction but did not meet the Bonferroni-adjusted threshold (*F* = 6.969, *P* = 0.008, pFDR = 0.030, ηp^2^ = 0.011). No significant group differences were observed for the right LC, right LDTg, bilateral PPTg, or DR. These findings identify the left LDTg as the most robust brainstem association, with potential additional involvement of the left LC.

**Table 1:** Focused analysis of predefined brainstem nuclei of interest

Structure	Group	*N*	Mean (mm^3^)	S.D. (mm^3^)	S.E.	*F*	*P*-value	pFDR	Observed power (α = 0.05)	Partial eta squared
DR	PD-mVHs^−^	577	162.2	32.7	1.4	2.359	0.125	0.219	0.339	0.004
	PD-mVHs^+^	64	156.0	33.0	4.1					
LC-L	PD-mVHs^−^	577	22.6	5.1	0.2	6.969	0.008	0.030	0.756	0.011
	PD-mVHs^+^	64	21.1	5.0	0.6					
LC-R	PD-mVHs^−^	577	20.4	5.4	0.2	3.365	0.067	0.157	0.454	0.005
	PD-mVHs^+^	64	19.4	5.6	0.7					
LDTg-L	PD-mVHs^−^	577	4.2	1.8	0.1	7.955	0.005	0.030	0.810	0.012
	PD-mVHs^+^	64	3.6	1.6	0.2					
LDTg-R	PD-mVHs^−^	577	3.6	1.6	0.1	1.770	0.184	0.257	0.267	0.003
	PD-mVHs^+^	64	3.5	1.7	0.2					
PPTg-L	PD-mVHs^−^	577	55.9	7.6	0.3	1.211	0.272	0.317	0.198	0.002
	PD-mVHs^+^	64	56.0	8.6	1.1					
PPTg-R	PD-mVHs^−^	577	62.4	8.2	0.3	<0.001	0.985	0.985	0.050	<0.001
	PD-mVHs^+^	64	62.8	8.6	1.1					

Group volumes (mm^3^) are reported as means, standard deviations (S.D.), and standard errors (S.E.). ANCOVA models included group and sex as fixed factors, together with the group × sex interaction, and age, education, standardized eTIV, MoCA score, and disease duration as continuous covariates. The reported *F* statistics, *P*-values, FDR-adjusted *P*-values, observed power estimates, and partial eta-squared values refer to the Type III main effect of group. No group × sex interaction was significant across the seven predefined regions. To account for multiple comparisons across the seven predefined brainstem regions, both Bonferroni and false discovery rate (FDR) corrections were considered. The Bonferroni-corrected significance threshold was *α* = 0.007 (0.05/7), and FDR-adjusted *P*-values (pFDR) were calculated using the Benjamini–Hochberg procedure. Left LDTg remained significant after both Bonferroni and FDR correction, whereas left LC remained significant after FDR correction but did not meet the Bonferroni-adjusted threshold.

*Abbreviations:* DR, dorsal raphe; eTIV, estimated total intracranial volume; FDR, false discovery rate; L, left; LC, locus coeruleus; LDTg, laterodorsal tegmental nucleus; MoCA, Montreal Cognitive Assessment; pFDR, FDR-adjusted *P*-value; PPTg, pedunculopontine tegmental nucleus; R, right; S.D., standard deviation; S.E., standard error; ηp^2^, partial eta squared.

Because MDS-UPDRS Part I item 1.2 may include formed hallucinations at scores above 1, we examined the distribution of scores within the PD-mVHs^+^ group. Among the 64 PD-mVHs^+^ participants, 57 (89.1%) had a maximum score of 1, 5 (7.8%) had a score of 2, and 2 (3.1%) had a score of 3. When the sensitivity analysis was restricted to participants scoring exactly 1, PD–mVHs^+^ participants continued to show reduced volumes in the left LDTg and left LC compared with PD–mVHs^−^ participants. Both effects survived Bonferroni correction across the seven predefined brainstem nuclei (LDTg-L: *F* = 8.148, *P* = 0.004, pFDR = 0.016, ηp^2^ = 0.013; LC-L: *F* = 8.166, *P* = 0.004, pFDR = 0.016, ηp^2^ = 0.013). No significant group differences were observed for the right LC, right LDTg, bilateral PPTg, or DR.

### Exploratory analyses on subcortical and cortical structures

Exploratory regional ANCOVAs were conducted to contextualize the focused brainstem findings within broader cortical, subcortical, cerebellar, and additional brainstem regions (Tables S2–S7). Because these analyses involved screening across a large number of structures with no correction for multiple comparisons, the results should be interpreted as hypothesis-generating rather than confirmatory. Outside the a priori LC/LDTg/PPTg/DR set, group differences involved the VTA (Tables S2 and S3) as well as thalamic/epithalamic, hypothalamic, and cerebellar regions (Tables S4 and S5), including the left dentate lateral nucleus, right medial interpositus/globose nucleus, left lateral pulvinar, right lateral hypothalamic area (posterior part), right posterior hypothalamic nucleus, left habenular commissure, left stria medullaris of the thalamus, and right cerebellar white matter. In the neocortex, group differences were confined to a small set of higher-order associative regions, including the right inferior parietal, right lingual, right medial orbitofrontal, and bilateral middle temporal cortices (Tables S6 and S7).

#### Block 1—Demographic, clinical, and neuropsychological predictors

The baseline logistic regression model included the four clinical/neuropsychological variables that differed between groups in the preliminary analyses: disease duration, GDS score, HVLT-IR, and semantic fluency assessed using the animal-naming task (SF-Animal) (Tables S8–S9). The model was significant overall (*χ*^2^ = 36.092, df = 4, *P* < 0.001; Nagelkerke *R*^2^ = 0.115). Higher GDS scores, longer disease duration, and higher HVLT-IR scores were independently associated with increased odds of early hallucination-related status [GDS: *B* = 0.261, *P* = 0.002, odds ratio (OR) = 1.298; disease duration: *B* = 0.254, *P* = 0.001, OR = 1.289; HVLT-IR: *B* = 0.092, *P* = 0.003, OR = 1.097]. In contrast, higher SF-Animal scores were associated with lower odds of hallucination-related status (*B* = −0.094, *P* = 0.001, OR = 0.911). Bootstrap resampling supported the stability of all four coefficients, with their 95% confidence intervals excluding zero.

#### Block 2—Demographic, clinical, and neuropsychological predictors plus left LC and left LDTg

Adding left LC and left LDTg volumes to the clinical/neuropsychological Block 1 model significantly improved model fit on the likelihood-ratio test (Δ*χ*^2^ = 9.161, df = 2, *P* = 0.010). The resulting Block 2 model was significant overall (*χ*^2^ = 45.253, df = 6, *P* < 0.001; Nagelkerke *R*^2^ = 0.143; Tables 2 and  3). In line with the hypothesis-driven addition of selected MRI-derived brainstem predictors, the AIC decreased from 390.23 in Block 1 to 385.06 in Block 2, supporting improved model fit after accounting for model complexity. Clinical and neuropsychological predictors remained directionally stable, with GDS score (*B* = 0.273, *P* = 0.001, OR = 1.314), disease duration (*B* = 0.244, *P* = 0.002, OR = 1.276), and HVLT-IR (*B* = 0.093, *P* = 0.003, OR = 1.097) retaining positive associations with hallucination-related status, whereas SF-Animal retained an inverse association (*B* = −0.093, *P* = 0.001, OR = 0.911). Bootstrap confidence intervals excluded zero for all four clinical and neuropsychological predictors. Among the MRI-derived brainstem measures, left LDTg volume showed an inverse association with hallucination-related status (*B* = −0.182, *P* = 0.026, OR = 0.833), whereas left LC volume showed a similar negative direction but was not significant (*B* = −0.044, *P* = 0.107, OR = 0.957). Bootstrap resampling supported the stability of the LDTg coefficient, but not the LC coefficient.

**Table 2:** Hierarchical logistic regression model including selected clinical and neuropsychological variables and hypothesis-driven brainstem predictors of hallucination-related status

Variable	*B*	S.E.	Wald	*P*-value	Exp(*B*)
HVLT-IR	0.093	0.031	8.959	0.003	1.097
SF-Animal	−0.093	0.028	11.066	0.001	0.911
GDS	0.273	0.084	10.703	0.001	1.314
Disease duration (years)	0.244	0.078	9.862	0.002	1.276
LC-L	−0.044	0.027	2.604	0.107	0.957
LDTg-L	−0.182	0.082	4.933	0.026	0.833
Constant	−2.781	1.151	5.840	0.016	0.062

Block 1 included the clinical/neuropsychological predictors that differed between groups, namely disease duration, GDS score, HVLT-IR, and SF-Animal. Block 2 added left LC and left LDTg volumes. Higher HVLT-IR, GDS, and disease duration scores and lower SF-Animal scores were associated with hallucination-related status. Among the hypothesis-driven brainstem predictors, left LDTg showed an inverse association, indicating that smaller left LDTg volume was associated with greater odds of hallucination-related status, whereas left LC was not significantly associated. −2 Log Likelihood (−2LL) = 371.064; Nagelkerke’s *R*^2^ = 0.143; Cox & Snell *R*^2^ = 0.068.

HVLT-IR, GDS, disease duration, SF-Animal, and left LDTg volume were significantly associated with hallucination-related status. Higher HVLT-IR, GDS, and disease duration scores were associated with greater odds, whereas higher SF-Animal scores and larger left LDTg volume were associated with lower odds. Left LC volume did not reach statistical significance. The addition of left LC and left LDTg volumes improved model fit relative to the clinical/neuropsychological model, with left LDTg emerging as an independent correlate of hallucination-related status.

*Abbreviations*: B, regression coefficient; GDS, Geriatric Depression Scale; HVLT-IR, Hopkins Verbal Learning Test-Revised—Immediate Recall; L, left; LC, locus coeruleus; LDTg, laterodorsal tegmental nucleus; S.E., standard error; SF-Animal, Semantic Fluency—Total Number of Animals; Exp(B), odds ratio.

**Table 3:** Bootstrap-estimated regression coefficients for the clinical and hypothesis-driven brainstem model

Variable	*B*	Bias	S.E.	*P*-value	BCa 95% CI lower	BCa 95% CI upper
HVLT-IR	0.093	0.002	0.032	0.003	0.029	0.165
SF-Animal	−0.093	−0.003	0.030	0.002	−0.154	−0.042
GDS	0.273	0.007	0.093	0.003	0.067	0.478
Disease duration (years)	0.244	0.006	0.094	0.006	0.049	0.463
LC-L	−0.044	<0.001	0.029	0.114	−0.102	0.014
LDTg-L	−0.182	−0.009	0.085	0.024	−0.345	−0.044
Constant	−2.781	−0.065	1.133	0.012	−4.960	−0.790

Bootstrap regression coefficients and BCa 95% confidence intervals are reported for the final Block 2 model. HVLT-IR, GDS, and disease duration showed bootstrap-supported positive associations, whereas SF-Animal showed a bootstrap-supported inverse association with hallucination-related status. Among the hypothesis-driven brainstem predictors, left LDTg showed a bootstrap-supported inverse association, whereas the confidence interval for left LC crossed zero. The bootstrap analysis supported positive associations of HVLT-IR, GDS, and disease duration and an inverse association of SF-Animal with hallucination-related status. Among the brainstem predictors included on an a priori basis, only left LDTg showed a bootstrap-supported association, whereas the BCa 95% confidence interval for left LC crossed zero.

*Abbreviations:* B, regression coefficient; BCa, bias-corrected and accelerated; GDS, Geriatric Depression Scale; HVLT-IR, Hopkins Verbal Learning Test-Revised—Immediate Recall; L, left; LC, locus coeruleus; LDTg, laterodorsal tegmental nucleus; S.E., bootstrap standard error; SF-Animal, Semantic Fluency—Total Number of Animals.

We then repeated the main Block 2 logistic regression in the restricted score-1 subgroup. Left LDTg volume remained inversely associated with hallucination-related status (*B* = −0.184, *P* = 0.032, OR = 0.832), with bootstrap resampling supporting coefficient stability (BCa 95% CI = −0.353 to −0.037). Left LC volume showed the same negative direction but was not significant in the multivariable model (*B* = −0.048, *P* = 0.095, OR = 0.954), and its bootstrap confidence interval crossed zero (BCa 95% CI = −0.109 to 0.009). These findings support the robustness of the left LDTg association within the subgroup of PD–mVHs^+^ participants whose scores are most compatible with minor hallucination-related phenomena.

#### Block 3—Exploratory extended model including LC/LDTg and candidate morphometric regions

Block 3 added the candidate regions identified from the exploratory regional ANCOVAs to the Block 2 predictors (Tables 4 and 5). The extended model was significant overall, *χ*^2^(20) = 77.276, *P* < 0.001, with Nagelkerke *R*^2^ = 0.238 and −2 log likelihood (−2LL) = 339.040. Relative to Block 2, the addition of the 14 candidate morphometric predictors significantly improved model fit, likelihood-ratio *χ*^2^(14) = 32.024, *P* = 0.004, while Nagelkerke pseudo-R^2^ increased descriptively from 0.143 to 0.238. Within this exploratory model, HVLT immediate recall showed a positive association with hallucination-related status (*B* = 0.097, *P* = 0.003, OR = 1.102), with its BCa 95% confidence interval excluding zero. SF-Animal showed an inverse association with hallucination-related status (*B* = −0.099, *P* = 0.001, OR = 0.906), with its BCa 95% confidence interval excluding zero. GDS score also showed a positive association (*B* = 0.274, *P* = 0.001, OR = 1.315), with its BCa 95% confidence interval excluding zero. Disease duration showed a positive association (*B* = 0.293, *P* < 0.001, OR = 1.341), with its BCa 95% confidence interval also excluding zero.

**Table 4:** Exploratory extended logistic regression model including LC/LDTg and candidate morphometric regions

Variable	*B*	S.E.	Wald	*P*-value	Exp(*B*)
HVLT-IR	0.097	0.033	8.673	0.003	1.102
SF-Animal	−0.099	0.030	10.984	0.001	0.906
GDS	0.274	0.085	10.401	0.001	1.315
Disease duration (years)	0.293	0.083	12.397	<0.001	1.341
LC-L	−0.062	0.032	3.691	0.055	0.940
LDTg-L	−0.210	0.086	5.907	0.015	0.811
VTA	0.004	0.006	0.483	0.487	1.004
Dentate lateral nucleus-L	−0.004	0.001	8.699	0.003	0.996
Lateral hypothalamic area posterior part-R	0.014	0.012	1.325	0.250	1.014
Lateral nucleus of pulvinar left	−0.007	0.004	3.267	0.071	0.993
Medial interpositus globose nucleus-R	−0.056	0.028	3.865	0.049	0.946
Posterior hypothalamic nucleus-R	−0.006	0.009	0.396	0.529	0.994
Habenular commissure-L	0.654	0.265	6.091	0.014	1.924
Stria medullaris of thalamus-L	−0.037	0.016	5.206	0.023	0.964
White matter of the cerebellum-R	<0.001	<0.001	2.784	0.095	1.000
ctx inferiorparietal-R	<0.001	<0.001	0.323	0.570	1.000
ctx middletemporal-L	<0.001	<0.001	0.342	0.558	1.000
ctx middletemporal-R	<0.001	<0.001	0.032	0.859	1.000
ctx lingual-R	<0.001	<0.001	0.251	0.616	1.000
ctx medialorbitofrontal-R	<0.001	<0.001	0.019	0.890	1.000
Constant	−1.052	1.809	0.338	0.561	0.349

Block 3 included the clinical/neuropsychological predictors, left LC and left LDTg volumes, and candidate cortical, subcortical, cerebellar, thalamic, hypothalamic, and additional brainstem regions identified from exploratory regional ANCOVAs. HVLT-IR, SF-Animal, GDS, disease duration, left LDTg, left dentate lateral nucleus, right medial interpositus/globose nucleus, left habenular commissure, and left stria medullaris of the thalamus were associated with hallucination-related status at the uncorrected *P* ≤ 0.05 level. −2 Log Likelihood (−2LL) = 339.040; Nagelkerke’s *R*^2^ = 0.238; Cox & Snell *R*^2^ = 0.114.

In this exploratory extended model, HVLT-IR, GDS, and disease duration showed positive associations with hallucination-related status, whereas SF-Animal, left LDTg, left dentate lateral nucleus, right medial interpositus/globose nucleus, and left stria medullaris of the thalamus showed inverse associations. Left habenular commissure showed a positive association. Left LC and left lateral pulvinar did not retain significant associations after mutual adjustment. Because candidate regions were selected from uncorrected exploratory screening and the event-per-variable ratio was low, these findings should be interpreted cautiously as hypothesis-generating.

*Abbreviations:* B, regression coefficient; ctx, cortex; GDS, Geriatric Depression Scale; HVLT-IR, Hopkins Verbal Learning Test-Revised—Immediate Recall; L, left; LC, locus coeruleus; LDTg, laterodorsal tegmental nucleus; R, right; S.E., standard error; SF-Animal, Semantic Fluency—Total Number of Animals; VTA, ventral tegmental area; Exp(B), odds ratio.

**Table 5:** Bootstrap-estimated regression coefficients for the exploratory extended model including LC/LDTg

Variable	*B*	Bias	S.E.	*P*-value	BCa 95% CI lower	BCa 95% CI upper
HVLT-IR	0.097	0.008	0.034	0.003	0.027	0.195
SF-Animal	−0.099	−0.006	0.035	0.002	−0.166	−0.049
GDS	0.274	0.021	0.102	0.002	0.044	0.560
Disease duration (years)	0.293	0.022	0.105	0.002	0.047	0.576
LC-L	−0.062	−0.005	0.037	0.065	−0.133	−0.006
LDTg-L	−0.210	−0.018	0.093	0.013	−0.384	−0.085
VTA	0.004	<0.001	0.006	0.444	−0.008	0.016
Dentate lateral nucleus-L	−0.004	<0.001	0.002	0.009	−0.008	−0.002
Lateral hypothalamic area posterior part-R	0.014	<0.001	0.014	0.265	−0.013	0.042
Lateral nucleus of pulvinar left	−0.007	−0.001	0.005	0.077	−0.016	−0.0002
Medial interpositus globose nucleus-R	−0.056	−0.004	0.037	0.100	−0.124	0.005
Posterior hypothalamic nucleus-R	−0.006	<0.001	0.011	0.582	−0.027	0.018
Habenular commissure-L	0.654	0.058	0.319	0.023	−0.025	1.485
Stria medullaris of thalamus-L	−0.037	−0.002	0.017	0.018	−0.069	−0.010
White matter of the cerebellum-R	<0.001	<0.001	<0.001	0.087	−0.000063	0.000443
ctx inferiorparietal-R	<0.001	<0.001	<0.001	0.559	−0.000260	0.000136
ctx middletemporal-L	<0.001	<0.001	<0.001	0.588	−0.000358	0.000212
ctx middletemporal-R	<0.001	<0.001	<0.001	0.875	−0.000261	0.000290
ctx lingual-R	<0.001	<0.001	<0.001	0.649	−0.000353	0.000589
ctx medialorbitofrontal-R	<0.001	<0.001	<0.001	0.890	−0.000339	0.000355
Constant	−1.052	0.058	2.018	0.568	−5.249	3.191

Bootstrap regression coefficients and BCa 95% confidence intervals are reported for Block 3. BCa 95% confidence intervals excluded zero for HVLT-IR, SF-Animal, GDS, disease duration, left LC, left LDTg, left dentate lateral nucleus, left lateral pulvinar, and left stria medullaris of the thalamus. In contrast, the BCa confidence intervals for the right medial interpositus/globose nucleus, left habenular commissure, and the remaining exploratory predictors included zero. These results should be regarded as hypothesis-generating. Bootstrap BCa 95% confidence intervals excluded zero for HVLT-IR, SF-Animal, GDS, disease duration, left LC, left LDTg, left dentate lateral nucleus, left lateral pulvinar, and left stria medullaris of the thalamus. In contrast, the BCa confidence intervals for the right medial interpositus/globose nucleus, left habenular commissure, and the remaining exploratory predictors included zero. These findings should be regarded as hypothesis-generating.

*Abbreviations:* B, regression coefficient; BCa, bias-corrected and accelerated; ctx, cortex; GDS, Geriatric Depression Scale; HVLT-IR, Hopkins Verbal Learning Test-Revised—Immediate Recall; L, left; LC, locus coeruleus; LDTg, laterodorsal tegmental nucleus; R, right; S.E., bootstrap standard error; SF-Animal, Semantic Fluency—Total Number of Animals; VTA, ventral tegmental area.

Among the brainstem predictors, left LDTg volume showed an inverse association with hallucination-related status (*B* = −0.210, *P* = 0.015, OR = 0.811), with its BCa 95% confidence interval excluding zero. Left LC volume showed an inverse association that did not reach the conventional significance threshold in the standard model (*B* = −0.062, *P* = 0.055, OR = 0.940), although its BCa 95% confidence interval excluded zero. Among the exploratory morphometric predictors, left dentate lateral nucleus volume showed a nominal inverse association with hallucination-related status (*B* = −0.004, *P* = 0.003, OR = 0.996), with its BCa 95% confidence interval excluding zero. Right medial interpositus/globose nucleus volume showed a nominal inverse association (*B* = −0.056, *P* = 0.049, OR = 0.946), although its BCa 95% confidence interval included zero. Left habenular commissure volume showed a nominal positive association (*B* = 0.654, *P* = 0.014, OR = 1.924), although its BCa 95% confidence interval included zero. Left stria medullaris of the thalamus showed a nominal inverse association (*B* = −0.037, *P* = 0.023, OR = 0.964), with its BCa 95% confidence interval excluding zero. The left lateral nucleus of the pulvinar did not reach the conventional significance threshold in the standard model after mutual adjustment (*B* = −0.007, *P* = 0.071, OR = 0.993), although its BCa 95% confidence interval excluded zero. The remaining cortical, subcortical, cerebellar, thalamic, hypothalamic, and additional brainstem variables did not show associations at the uncorrected *P* ≤ 0.05 level after mutual adjustment. Because the candidate regions were selected through uncorrected exploratory screening in the same dataset, both the individual coefficients and the incremental model comparison should be interpreted as exploratory and hypothesis-generating.

#### Block 4—Exploratory extended model excluding left LC and left LDTg

Block 4 repeated the extended exploratory model after removing left LC and left LDTg (Tables 6 and 7). The model remained significant overall, *χ*^2^(18) = 66.522, *P* < 0.001, with Nagelkerke *R*^2^ = 0.206 and −2 log likelihood (−2LL) = 349.794. Compared with Block 3, removal of left LC and left LDTg significantly worsened model fit, likelihood-ratio *χ*^2^(2) = 10.754, *P* = 0.005, while Nagelkerke *R*^2^ decreased descriptively from 0.238 to 0.206. Among the clinical and neuropsychological predictors, GDS score showed a positive association with hallucination-related status (*B* = 0.248, *P* = 0.003, OR = 1.282), as did disease duration (*B* = 0.296, *P* < 0.001, OR = 1.344). HVLT immediate recall showed a positive association with hallucination-related status (*B* = 0.092, *P* = 0.004, OR = 1.096). SF-Animal showed an inverse association with hallucination-related status (*B* = −0.092, *P* = 0.002, OR = 0.912). BCa bootstrap intervals supported all four clinical and neuropsychological effects, with their 95% confidence intervals excluding zero. Among the exploratory morphometric variables, reduced left dentate lateral nucleus volume showed an inverse association with hallucination-related status (*B* = −0.004, *P* = 0.003, OR = 0.996), with its BCa 95% confidence interval excluding zero (BCa 95% CI = −0.007 to −0.002). Left habenular commissure volume showed a nominal positive association (*B* = 0.612, *P* = 0.018, OR = 1.844), although its BCa confidence interval included zero (BCa 95% CI = −0.022 to 1.367). Right medial interpositus/globose nucleus volume showed a nominal inverse association in the standard model (*B* = −0.057, *P* = 0.040, OR = 0.945), although its BCa confidence interval included zero. Left stria medullaris of the thalamus also showed a nominal inverse association (*B* = −0.032, *P* = 0.038, OR = 0.968), with its BCa confidence interval excluding zero (BCa 95% CI = −0.063 to −0.009). The left lateral nucleus of the pulvinar did not remain significantly associated with hallucination-related status after mutual adjustment (*B* = −0.006, *P* = 0.123, OR = 0.994), and its BCa confidence interval included zero. The remaining exploratory cortical, subcortical, cerebellar, thalamic, hypothalamic, and additional brainstem predictors did not show significant associations with hallucination-related status after mutual adjustment. These findings indicate that, after removal of left LC and left LDTg, only a limited subset of regions retained evidence of association with hallucination-related status. However, because Block 4 was based on regions selected from uncorrected exploratory screening and remained affected by a low event-per-variable ratio, it should be interpreted as an exploratory sensitivity model rather than as a definitive test of independent regional contributions.

**Table 6:** Exploratory extended logistic regression model excluding left LC and left LDTg

Variable	*B*	S.E.	Wald	*P*-value	Exp(*B*)
HVLT-IR	0.092	0.032	8.097	0.004	1.096
SF-Animal	−0.092	0.029	9.945	0.002	0.912
GDS	0.248	0.083	8.996	0.003	1.282
Disease duration (years)	0.296	0.082	13.049	<0.001	1.344
VTA	0.002	0.005	0.140	0.709	1.002
Dentate lateral nucleus-L	−0.004	0.001	8.871	0.003	0.996
Lateral hypothalamic area posterior part-R	0.010	0.012	0.639	0.424	1.010
Lateral nucleus of pulvinar left	−0.006	0.004	2.385	0.123	0.994
Medial interpositus globose nucleus-R	−0.057	0.028	4.204	0.040	0.945
Posterior hypothalamic nucleus-R	−0.002	0.009	0.064	0.801	0.998
Habenular commissure-L	0.612	0.258	5.640	0.018	1.844
Stria medullaris of thalamus-L	−0.032	0.015	4.326	0.038	0.968
White matter of the cerebellum-R	<0.001	<0.001	1.088	0.297	1.000
ctx inferiorparietal-R	<0.001	<0.001	0.397	0.529	1.000
ctx middletemporal-L	<0.001	<0.001	0.353	0.553	1.000
ctx middletemporal-R	<0.001	<0.001	0.143	0.705	1.000
ctx lingual-R	<0.001	<0.001	0.011	0.915	1.000
ctx medialorbitofrontal-R	<0.001	<0.001	0.029	0.864	1.000
Constant	−1.416	1.776	0.636	0.425	0.243

Block 4 repeated the extended exploratory model after removing left LC and left LDTg volumes. HVLT-IR, SF-Animal, GDS, disease duration, left dentate lateral nucleus, right medial interpositus/globose nucleus, left habenular commissure, and left stria medullaris of the thalamus were associated with hallucination-related status at the uncorrected *P* ≤ 0.05 level. −2 Log Likelihood (−2LL) = 349.794; Nagelkerke’s *R*^2^ = 0.206; Cox & Snell *R*^2^ = 0.099.

After removal of left LC and left LDTg, HVLT-IR, GDS, and disease duration showed positive associations with hallucination-related status, whereas SF-Animal, left dentate lateral nucleus, right medial interpositus/globose nucleus, and left stria medullaris of the thalamus showed inverse associations. Left habenular commissure showed a positive association. The remaining exploratory anatomical predictors did not show significant associations after mutual adjustment. Although Block 4 contained two fewer predictors than Block 3, it showed lower explained variance and poorer fit relative to Block 3. Given the uncorrected exploratory selection of candidate regions and the low event-per-variable ratio, the findings should be interpreted cautiously as hypothesis-generating.

*Abbreviations:* B, regression coefficient; ctx, cortex; Exp(B), odds ratio; GDS, Geriatric Depression Scale; HVLT-IR, Hopkins Verbal Learning Test-Revised—Immediate Recall; L, left; LC, locus coeruleus; LDTg, laterodorsal tegmental nucleus; R, right; S.E., standard error; SF-Animal, Semantic Fluency—Total Number of Animals; VTA, ventral tegmental area.

**Table 7:** Bootstrap-estimated regression coefficients for the exploratory extended model excluding LC/LDTg

Variable	*B*	Bias	S.E.	*P*-value	BCa 95% CI lower	BCa 95% CI upper
HVLT-IR	0.092	0.007	0.033	0.002	0.025	0.181
SF-Animal	−0.092	−0.005	0.034	0.002	−0.158	−0.042
GDS	0.248	0.020	0.099	0.006	0.025	0.511
Disease duration (years)	0.296	0.018	0.101	0.001	0.072	0.557
VTA	0.002	<0.001	0.005	0.678	−0.009	0.014
Dentate lateral nucleus-L	−0.004	<0.001	0.002	0.004	−0.007	−0.002
Lateral hypothalamic area posterior part-R	0.010	−0.001	0.014	0.435	−0.017	0.035
Lateral nucleus of pulvinar left	−0.006	<0.001	0.005	0.145	−0.015	0.001
Medial interpositus globose nucleus-R	−0.057	−0.001	0.035	0.083	−0.127	0.011
Posterior hypothalamic nucleus-R	−0.002	<0.001	0.011	0.819	−0.024	0.022
Habenular commissure-L	0.612	0.054	0.293	0.020	−0.022	1.367
Stria medullaris of thalamus-L	−0.032	−0.002	0.016	0.026	−0.063	−0.009
White matter of the cerebellum-R	<0.001	<0.001	<0.001	0.276	−0.000110	0.000318
ctx inferiorparietal-R	<0.001	<0.001	<0.001	0.528	−0.000259	0.000120
ctx middletemporal-L	<0.001	<0.001	<0.001	0.585	−0.000340	0.000202
ctx middletemporal-R	<0.001	<0.001	<0.001	0.730	−0.000222	0.000312
ctx lingual-R	<0.001	<0.001	<0.001	0.913	−0.000388	0.000448
ctx medialorbitofrontal-R	<0.001	<0.001	<0.001	0.867	−0.000353	0.000389
Constant	−1.416	0.050	1.976	0.441	−5.645	2.630

Bootstrap regression coefficients and BCa 95% confidence intervals are reported for Block 4. Bootstrap-supported associations were observed for HVLT-IR, SF-Animal, GDS, disease duration, left dentate lateral nucleus, and left stria medullaris of the thalamus. Left habenular commissure, right medial interpositus/globose nucleus, and the remaining exploratory predictors showed less stable estimates, with confidence intervals overlapping zero. These results should be regarded as hypothesis-generating. BCa 95% confidence intervals excluded zero for HVLT-IR, SF-Animal, GDS, disease duration, left dentate lateral nucleus, and left stria medullaris of the thalamus, supporting the stability of these coefficients in the bootstrap analysis. In contrast, the confidence intervals for the right medial interpositus/globose nucleus and left habenular commissure included zero, despite their nominal associations in the standard logistic regression model, and the remaining exploratory anatomical predictors also showed confidence intervals overlapping zero. Block 4 was more parsimonious in terms of the number of predictors than Block 3, but removal of left LC and left LDTg significantly worsened model fit, likelihood-ratio *χ*^2^(2) = 10.754, *P* = 0.005. Nagelkerke pseudo-*R*^2^ decreased descriptively from 0.238 to 0.206. These exploratory findings should not be interpreted as definitive evidence of independent regional contributions.

*Abbreviations:* B, regression coefficient; BCa, bias-corrected and accelerated; ctx, cortex; GDS, Geriatric Depression Scale; HVLT-IR, Hopkins Verbal Learning Test-Revised—Immediate Recall; L, left; LC, locus coeruleus; LDTg, laterodorsal tegmental nucleus; R, right; S.E., bootstrap standard error; SF-Animal, Semantic Fluency—Total Number of Animals; VTA, ventral tegmental area.

#### Sensitivity analysis for scanner manufacturers and nigrostriatal asymmetry

As an additional sensitivity analysis, we repeated the main Block 2 logistic regression model including scanner manufacturer and nigrostriatal asymmetry index as additional covariates. Because direct clinical measures of motor laterality, such as side of motor onset or lateralized MDS-UPDRS Part III item-level scores, were not available, the volume asymmetry of the SNc was used as an anatomical proxy of nigral lateralization. The sensitivity model remained significant overall (*χ*^2^ = 47.459, df = 9, *P* < 0.001; Nagelkerke *R*^2^ = 0.149). The left LDTg volume remained inversely associated with hallucination-related status after adjustment for scanner manufacturer and nigrostriatal asymmetry (*B* = −0.191, *P* = 0.022, OR = 0.826), whereas the left LC volume was not significant (*B* = −0.044, *P* = 0.105, OR = 0.957). Scanner manufacturer was not significant as a global categorical predictor (*P* = 0.392), and the nigrostriatal asymmetry index was also not associated with hallucination-related status (*B* = −0.0005, *P* = 0.940, OR = 1.000). These findings suggest that the left LDTg association is unlikely to be explained by multi-vendor acquisition effects or by global nigrostriatal asymmetry, although the analysis does not eliminate residual scanner-, site-, or laterality-related confounding.

## Discussion

The present study examined MRI-derived volumetric correlates of early hallucination-related phenomena compatible with mVHs in PD, focusing on small neuromodulatory brainstem nuclei estimated using histology-informed probabilistic atlases. PD-mVHs^+^ participants showed reduced left LC and left LDTg volumes compared with PD-mVHs^−^ participants, but only the left LDTg association remained significant under Bonferroni correction. Adding left LC and left LDTg volumes to the clinical/neuropsychological baseline model improved overall fit. Although clinical and neuropsychological variables remained important contributors to hallucination-related status, the left LDTg provided the clearest volumetric association in the brainstem-focused model, yielding a directionally consistent negative coefficient and a bootstrap-supported association. We also demonstrate that this finding is unlikely to be explained by multi-vendor acquisition effects or by global nigrostriatal asymmetry. When a broader set of candidate morphometric regions identified from exploratory regional ANCOVAs was added to an exploratory model, only a limited subset of regions retained an association after mutual adjustment. Crucially, the left LDTg remained directionally stable. Overall, the present results suggest that hallucination-related status in this cohort reflects a combination of clinical factors, particularly disease duration and depressive symptoms, neuropsychological performance, including lower semantic fluency and higher immediate verbal recall, and MRI-derived volumetric variation in selected neuromodulatory brainstem regions, most robustly the left LDTg.

The directionally consistent LDTg association across modelling stages, coupled with its robustness in the brainstem-focused block, supports an association between reduced LDTg volume and early hallucination-related vulnerability in PD. The LDTg provides ascending cholinergic and glutamatergic projections to the thalamus, midbrain, and cortex, as well as descending projections to the pontine reticular formation and spinal targets.^59^ Through these pathways, it plays a central role in arousal regulation, REM sleep generation, and sensory gating, processes repeatedly implicated in the emergence of hallucinations in PD and related disorders.^24^ Reduced LDTg volume may therefore reflect the disruption of cholinergic brainstem systems involved in filtering sensory inputs and stabilizing perceptual states. In line with this interpretation, neuropathological and molecular imaging studies have demonstrated cholinergic neuronal loss in the pontine tegmentum of PD patients with hallucinations,^30,60,61^ while PET imaging using acetylcholinesterase tracers shows reduced cortical cholinergic activity in hallucinators.^62^

Notably, the LDTg is a key component of the ascending reticular activating system and interacts functionally with the PPTg and basal forebrain cholinergic complex.^59^ Although the PPTg did not show a significant difference between groups, its close anatomical and functional coupling with the LDTg suggests that degeneration within this cholinergic network may impair thalamocortical transmission and attentional modulation. Overall, our results extend this evidence by identifying the LDTg as an MRI-derived structural correlate of early hallucination-related vulnerability in PD. Nevertheless, we emphasize that, given the cross-sectional imaging design and the use of T1-weighted volumetry, the directionality and neurochemical specificity of this association remain inferential rather than causal.

The LC showed a convergent, but less robust, pattern. In the focused volumetric analysis, left LC volume was reduced in PD–mVHs^+^ participants, but this association did not survive Bonferroni correction, and the predictor was not independently retained in the main logistic regression model. In Block 3, left LC showed a directionally consistent inverse coefficient that did not reach nominal significance in the standard logistic regression, although its BCa 95% confidence interval excluded zero. This finding emerged only within an exploratory extended model containing a relatively large number of predictors and should therefore not supersede the non-significant result obtained in the primary Block 2 model. Moreover, joint removal of left LC and left LDTg significantly worsened model fit, although this comparison does not establish the independent contribution of either region. Overall, the LC pattern remains less robust and less consistent than the LDTg association. This result should also be interpreted with caution, given that the locus coeruleus is one of the smallest and most challenging brainstem nuclei for in vivo segmentation. Thus, reduced measurement precision may attenuate detectable effects. Nevertheless, the LC remains theoretically relevant given its integrative role in arousal, attention, and sensory processing. It is among the earliest sites of Lewy pathology^38,63^ and exhibits neuronal loss comparable to or greater than the substantia nigra.^64^ LC degeneration has been linked to depressive symptoms, cognitive slowing, dysautonomia, and sleep–wake disturbances.^34,40^ Within this broader framework, the present findings suggest that reduced LC integrity may contribute, directly or indirectly, to perceptual instability. A decline in cortical noradrenergic tone could weaken attentional selection and sensory filtering, consistent with noradrenaline’s role in modulating signal-to-noise ratios in sensory networks^35,36^ and in regulating transitions between wakefulness and REM states.^65^ Moreover, the LC interacts with dopaminergic and cholinergic systems: noradrenaline can facilitate basal forebrain cholinergic output^66^ and activate D2-like receptors.^67^ LC degeneration could therefore exert cascading effects on dopaminergic–cholinergic balance, potentially increasing hallucinatory vulnerability even when dopaminergic loss is relatively limited.

The absence of DR effects in our models is also informative. The DR is a major source of serotonergic projections to cortical and limbic regions involved in mood and higher-order perceptual integration. Serotonergic dysfunction has been linked to cVHs in PD,^4,27^ while raphe serotonergic alterations are detectable even in early PD.^68^ Nevertheless, their specific contribution to hallucination-related phenomena may become more prominent at later stages of the hallucination spectrum. This interpretation aligns with the clinical progression of hallucinations in PD: mVHs are typically simple, fleeting misperceptions (e.g. presence or passage hallucinations), whereas cVHs involve detailed, formed images often associated with cognitive decline and broader serotonergic and cortical dysfunction.^69,70^ The lack of DR involvement in our cohort, composed of patients with early PD, is therefore compatible with the possibility that structural involvement of serotonergic brainstem nuclei may be less prominent at this early hallucination-related stage, although this interpretation remains speculative.

We stress, however, that the present findings are based on T1-weighted MRI-derived volumetry and therefore do not provide direct evidence of neurotransmitter-specific degeneration. Although the LC and LDTg are anatomically and neurochemically relevant to noradrenergic and cholinergic systems, respectively, reduced regional volume should be interpreted as an indirect structural proxy. Such volume reductions may reflect multiple biological processes, including neuronal loss, gliosis, changes in neuropil organization, or other microstructural alterations, as well as methodological factors such as partial-volume effects or segmentation variability. Therefore, although the present results are consistent with cholinergic or noradrenergic neuronal loss, a firm conclusion can only be achieved through an independent and direct confirmation. Multimodal studies combining structural MRI with neuromelanin-sensitive MRI, cholinergic PET, noradrenergic PET when available, or post-mortem validation will be needed to directly test neurotransmitter-specific mechanisms.

The exploratory regional analyses suggested that additional subcortical, cerebellar, thalamic, hypothalamic, and cortical structures may be associated with hallucination-related vulnerability. However, the candidate regions entered into the analysis were selected from an exploratory screening that involved no correction for multiple comparisons. Therefore, these secondary findings should be interpreted cautiously and treated as hypothesis-generating pending replication. With this caveat in mind, reduced left dentate lateral nucleus volume showed an association with hallucination-related status in the exploratory extended models, with BCa confidence intervals excluding zero. As the largest cerebellar output nucleus, the dentate contributes to sensorimotor prediction, temporal coordination, and multimodal integration^71^; its alteration may therefore be relevant to models proposing that impaired predictive coding increases susceptibility to sensory misinterpretation.^21,72^ The positive association involving the left habenular commissure was nominally significant in the standard logistic regression, but its BCa confidence interval included zero, indicating limited bootstrap support. Accordingly, its possible involvement in reward- and salience-related systems should be interpreted cautiously. The habenula integrates dopaminergic and serotonergic signals and contributes to motivational valence and sensory salience.^73^ An inverse association involving the left stria medullaris of the thalamus was supported by BCa confidence intervals excluding zero in both exploratory models, although this finding remains hypothesis-generating. Thalamic findings involving the stria medullaris and trends involving the pulvinar are compatible with a possible contribution of thalamocortical and habenular circuitry to sensory filtering, attention, and salience processing, given the previous links between thalamic dysfunction, pareidolic misperceptions in PD^74^ and hallucinations in DLB.^19^ Taken together, these exploratory findings may point to a broader distributed vulnerability encompassing brainstem neuromodulatory nuclei, cerebellar predictive systems, and thalamo–hypothalamic circuits involved in arousal and salience assignment.^69,70^ However, this hypothesis requires independent replication using corrected or cross-validated analytical approaches.

The left-lateralized associations observed in the present study should also be interpreted cautiously. Parkinson’s disease is characteristically asymmetric, and lateralized structural findings may partly reflect disease laterality rather than hallucination-specific mechanisms.^75,76^ In particular, if patients with hallucination-related phenomena had a different distribution of motor onset side or clinically more affected side, this could contribute to apparent left-sided brainstem effects. Therefore, the left-lateralized LC/LDTg associations observed in this sample should be tested in future studies with explicit adjustment for motor laterality. In a sensitivity model including scanner manufacturer and a SNc asymmetry index, the left LDTg association remained significant, whereas the asymmetry index was not associated with hallucination-related status. This suggests that the left LDTg finding could not be explained by global nigrostriatal asymmetry. However, the SNc asymmetry index represents only an indirect anatomical proxy and does not replace direct clinical measures of motor laterality, such as side of motor onset or lateralized MDS-UPDRS Part III item-level scores. Hemispheric asymmetries in PD are well documented^75,76^ and may reflect disease-related vulnerability rather than hallucination-specific mechanisms.^77^ Of note, when the sensitivity analysis was restricted to participants scoring exactly 1, both the left LDTg and left LC volume reductions remained significant and survived Bonferroni correction across the seven predefined brainstem nuclei. The left LC effect was numerically stronger in this restricted subgroup than in the full PD-mVHs^+^ group despite the smaller sample. This pattern supports the robustness of the left-sided brainstem findings within participants showing the mildest hallucination-related phenomena and is compatible with the possibility that scores ≥2 capture phenomenologically distinct experiences. However, given the small number of participants with scores ≥2 (*n* = 7), this observation should remain descriptive.

Several methodological strengths enhance our confidence in the results. The use of the PPMI cohort ensured standardized assessments, harmonized 3T MRI acquisition, and diagnostic homogeneity. The use of multi-atlas segmentation^44^ extended anatomical coverage to nuclei not routinely accessible in large datasets, while avoiding the operator dependence of manual tracing. Bootstrap resampling provided an assessment of coefficient stability. Incremental model fit was formally evaluated using likelihood-ratio tests based on changes in −2 log likelihood, while changes in Nagelkerke pseudo-*R*^2^ were reported descriptively to facilitate interpretation of model fit across blocks. In addition, treating HistoAtlas-derived and whole-brain analyses as exploratory helped to distinguish primary hypothesis-driven inference from hypothesis-generating contextual analyses.

Nonetheless, several limitations of the present study must be acknowledged. First, hallucination phenotyping relied on MDS-UPDRS Part I item 1.2, which is available in large multicentre cohorts but is not a dedicated instrument for mVHs. This item does not allow a fine-grained distinction among illusions, presence hallucinations, passage hallucinations, other visual hallucination phenomena, or non-visual psychosis-related symptoms. Therefore, our classification should be interpreted as a proxy for early hallucination-related phenomena compatible with mVHs, rather than as a definitive phenomenological diagnosis of mVHs. Future studies should employ dedicated structured interviews or hallucination-specific scales to distinguish minor visual hallucination subtypes and to test whether the present brainstem associations differ across specific hallucinatory phenomena.

Second, PD treatment represents an important potential confounder. A subset of participants was already receiving treatment for PD symptoms at baseline, while additional participants may have initiated dopaminergic therapy during follow-up. Hallucination-related status was defined using information collected at screening, baseline, and during the early follow-up period. Dopamine agonists are established risk factors for hallucinations in PD, and we could not consistently determine detailed medication exposure, treatment timing, levodopa equivalent daily dose, or dopamine agonist use at the time of hallucination-related assessment. Therefore, medication-related effects may have contributed to the presence or reporting of hallucination-related symptoms in a subset of PD–mVHs^+^ participants. This limitation prevents us from making strong claims that the observed associations are entirely independent of dopaminergic treatment. Future longitudinal studies should explicitly model dopaminergic medication exposure and test whether baseline LC and LDTg volumes predict hallucination-related outcomes independently of baseline treatment status, subsequent treatment initiation, dose, and drug class.

Third, although the overall imaging sample was adequate for the focused volumetric analyses, the number of hallucination-positive cases was modest, reflecting the early-stage and predominantly untreated-at-baseline composition of the study sample and resulting in class imbalance. This imbalance may have limited sensitivity for detecting subtle neuroanatomical effects and may have contributed to the stronger influence of clinical and neuropsychological variables in multivariable models. Model estimates may have been affected by class imbalance. To address this limitation, we adopted a hypothesis-driven modelling strategy and assessed coefficient stability using BCa bootstrap procedures. Nevertheless, replication in independent cohorts with larger hallucination-positive samples will be necessary to confirm the generalizability of these findings.

Fourth, volumetric estimation of very small brainstem nuclei, including the LC, LDTg, and PPTg, remains technically challenging with conventional 3T T1-weighted MRI. These nuclei are small relative to voxel size, show limited intrinsic contrast with surrounding brainstem tissue, and are therefore segmented primarily through atlas-informed probabilistic information rather than direct tissue contrast. Partial-volume effects, contrast variability, B0-related distortion, geometric variability, and interindividual anatomical differences may therefore have influenced the volumetric estimates, particularly for the locus coeruleus. In addition, PPMI is a multi-site and multi-vendor study. Although scanner manufacturer was included in a sensitivity analysis and did not abolish the left LDTg association, this adjustment cannot substitute for full site-level harmonization. Future studies should replicate these findings using explicit site-level modelling, ComBat-like harmonization approaches,^78^ higher-resolution structural MRI, neuromelanin-sensitive imaging for the LC, cholinergic PET, or ultra-high-field MRI. The absolute between-group differences were small in absolute terms, and the effect sizes modest (ηp^2^ ≈ 0.01–0.012). These findings should therefore be read as group-level associations rather than individually diagnostic.

Fifth, the cross-sectional imaging design precludes inferences about temporal or causal relationships between brainstem structural alterations and hallucination-related status. It therefore remains unclear whether reduced LC or LDTg volume precedes hallucination-related vulnerability or reflects a downstream correlate of broader disease progression. Longitudinal imaging studies will be required to clarify the temporal sequence of these alterations.

Finally, the exploratory whole-brain analyses and the extended regression models should be interpreted cautiously. The broader regional ANCOVAs were conducted to contextualize the hypothesis-driven brainstem findings within wider networks potentially relevant to hallucination vulnerability, but they were not corrected for multiple comparisons. Using regions selected from these uncorrected exploratory analyses to populate Blocks 3 and 4 may have propagated type I error and inflated the apparent contribution of additional neuroimaging variables. Moreover, blocks 3 and 4 included a large number of candidate predictors relative to the 64 hallucination-positive cases, resulting in a low event-per-variable ratio and a substantial risk of overfitting. Bootstrap resampling provided information about coefficient stability within a selected model, but it did not correct for the initial feature-selection step or resolve the underlying event-per-variable limitation. Accordingly, blocks 3 and 4 should be regarded strictly as exploratory sensitivity analyses, whereas the main inferential emphasis should remain on the hypothesis-driven brainstem ANCOVAs and the Block 2 regression model. Future studies should test broader morphometric associations using independent samples, correction for multiple comparisons, penalized regression, dimension-reduction approaches, or cross-validated feature-selection procedures.

## Conclusions

This study provides evidence that reduced T1-weighted MRI-derived volume in brainstem nuclei with cholinergic and noradrenergic relevance is associated with early hallucination-related vulnerability in PD. These volumetric associations were observed alongside clinical and neuropsychological factors, particularly depressive symptoms, disease duration, semantic fluency, and immediate verbal recall performance. Among the nuclei examined, the left LDTg showed the clearest and most robust association, whereas the left LC provided less robust evidence. Exploratory analyses further suggested that additional subcortical, cerebellar, and thalamic/hypothalamic regions may be involved in hallucination-related vulnerability. Overall, the results are consistent with a model in which early hallucination-related status in PD reflects distributed differences across neuromodulatory and subcortical systems, with the strongest evidence centred on the left LDTg, rather than focal degeneration of a single nucleus. Future studies should combine higher-resolution and neuromodulator-specific imaging—such as neuromelanin-sensitive MRI and cholinergic and noradrenergic PET—with longitudinal designs and dedicated phenomenological assessment of minor hallucinations to clarify the temporal sequence, causal direction, and neurochemical specificity of these brainstem associations, and to test their value for early risk stratification in PD.

## Supplementary Material

fcag357_Supplementary_Data

## Data Availability

Data used in this study are available through the Parkinson’s Progression Markers Initiative upon approved access. No custom analysis code was generated for this study.

## References

[1] Aarsland D, Creese B, Politis M, et al Cognitive decline in Parkinson's disease. Nat Rev Neurol. 2017;13(4):217–231. 10.1038/nrneurol.2017.27 PMC5643027 28257128

[2] de Lau LM, Breteler MM. Epidemiology of Parkinson's disease. Lancet Neurol. 2006;5(6):525–535. 10.1016/S1474-4422(06)70471-9 16713924

[3] Lenka A, Jhunjhunwala KR, Saini J, Pal PK. Structural and functional neuroimaging in patients with Parkinson’s disease and visual hallucinations: A critical review. Parkinsonism Relat Disord. 2015;21(7):683–691. 10.1016/j.parkreldis.2015.04.005 25920541

[4] Huot P, Johnston TH, Darr T, et al Increased 5-HT2A receptors in the temporal cortex of parkinsonian patients with visual hallucinations. Mov Disord. 2010;25(10):1399–1408. 10.1002/mds.23083 20629135

[5] Titova N, Padmakumar C, Lewis SJG, Chaudhuri KR. Parkinson’s: A syndrome rather than a disease? J Neural Transm (Vienna). 2017;124(7):907–914. 10.1007/s00702-016-1667-6 PMC5514217 28028643

[6] Stahl SM . Parkinson’s disease psychosis as a serotonin-dopamine imbalance syndrome. CNS Spectr. 2016;21(5):355–359. 10.1017/S1092852916000602 27686027

[7] Lenka A, Pagonabarraga J, Pal PK, Bejr-Kasem H, Kulisevsky J. Minor hallucinations in Parkinson disease: A subtle symptom with major clinical implications. Neurology. 2019;93(6):259–266. 10.1212/WNL.0000000000007913 PMC6709995 31289146

[8] Onofrj M, Thomas A, Bonanni L. New approaches to understanding hallucinations in Parkinson's disease: Phenomenology and possible origins. Expert Rev Neurother. 2007;7(12):1731–1750. 10.1586/14737175.7.12.1731 18052766

[9] Fénelon G, Mahieux F, Huon R, Ziégler M. Hallucinations in Parkinson’s disease: Prevalence, phenomenology and risk factors. Brain. 2000;123(4):733–745. 10.1093/brain/123.4.733 10734005

[10] Bhome R, Thomas GEC, Zarkali A, Weil RS. Structural and functional imaging correlates of visual hallucinations in Parkinson’s disease. Curr Neurol Neurosci Rep. 2023;23(6):287–299. 10.1007/s11910-023-01267-1 PMC10257588 37126201

[11] Fénelon G, Soulas T, Cleret de Langavant L, Trinkler I, Bachoud-Lévi A-C. Feeling of presence in Parkinson's disease. J Neurol Neurosurg Psychiatry. 2011;82(11):1219–1224. 10.1136/jnnp.2010.234799 PMC3382202 21551471

[12] Firbank MJ, Parikh J, Murphy N, et al Reduced occipital GABA in Parkinson disease with visual hallucinations. Neurology. 2018;91(7):e675–e685. 10.1212/WNL.0000000000006007 PMC6105043 30021920

[13] Vignando M, Ffytche D, Lewis SJG, et al Mapping brain structural differences and neuroreceptor correlates in Parkinson's disease visual hallucinations. Nat Commun. 2022;13(1):519. 10.1038/s41467-022-28087-0 PMC8791961 35082285

[14] Ramírez-Ruiz B, Martí MJ, Tolosa E, et al Cerebral atrophy in Parkinson's disease patients with visual hallucinations. Eur J Neurol. 2007;14:750–756. 10.1111/j.1468-1331.2007.01768.x 17594330

[15] Watanabe H, Senda J, Kato S, et al Cortical and subcortical brain atrophy in Parkinson's disease with visual hallucination. Mov Disord. 2013;28(12):1732–1736. 10.1002/mds.25641 24150865

[16] Zarkali A, McColgan P, Leyland LA, Lees AJ, Weil RS. Visual hallucinations in Parkinson’s disease: Phenomenology, neuroimaging and clinical management. Prog Brain Res. 2020;252:63–95.

[17] Bertram K, & Williams DR. Visual hallucinations in the differential diagnosis of parkinsonism. J Neurol Neurosurg Psychiatry. 2012;83(4):448–452. 10.1136/jnnp-2011-300980 PMC3297805 22228724

[18] Matar E, Brooks D, Lewis SJG, Halliday GM. Limbic thalamus atrophy is associated with visual hallucinations in Lewy body disorders. Neurobiol Aging. 2022;112:122–128. 10.1016/j.neurobiolaging.2022.01.001 35151034

[19] Delli Pizzi S, Franciotti R, Tartaro A, et al Structural alteration of the dorsal visual network in DLB patients with visual hallucinations: A cortical thickness MRI study. PLoS One. 2014;9(1):e86624. 10.1371/journal.pone.0086624 PMC3900597 24466177

[20] Mehraram R, Peraza LR, Murphy NRE, et al Functional and structural brain network correlates of visual hallucinations in Lewy body dementia. Brain. 2022;145(6):2190–2205. 10.1093/brain/awac094 PMC9246710 35262667

[21] Pagonabarraga J, Soriano-Mas C, Llebaria G, López-Solà M, Pujol J, Kulisevsky J. Neural correlates of minor hallucinations in non-demented patients with Parkinson's disease. Parkinsonism Relat Disord. 2014;20(3):290–296. 10.1016/j.parkreldis.2013.11.017 24373690

[22] Bejr-Kasem H, Pagonabarraga J, Martínez-Horta S, et al Disruption of the default mode network and its intrinsic functional connectivity underlies minor hallucinations in Parkinson’s disease. Mov Disord. 2019;34(1):78–86. 10.1002/mds.27557 30536829

[23] Bejr-Kasem H, Sampedro F, Marín-Lahoz J, Martínez-Horta S, Pagonabarraga J, Kulisevsky J. Minor hallucinations reflect early gray matter loss and predict subjective cognitive decline in Parkinson's disease. Eur J Neurol. 2021;28(2):438–447. 10.1111/ene.14576 33032389

[24] Ignatavicius A, Matar E, Lewis SJG. Visual hallucinations in Parkinson’s disease: Spotlight on central cholinergic dysfunction. Brain. 2025;148(2):376–393. 10.1093/brain/awae289 PMC11788216 39252645

[25] Schultz W, Dayan P, Montague PR. A neural substrate of prediction and reward. Science. 1997;275(5306):1593–1599. 10.1126/science.275.5306.1593 9054347

[26] Schultz W . Dopamine reward prediction-error signalling: A two-component response. Nat Rev Neurosci. 2016;17(3):183–195. 10.1038/nrn.2015.26 PMC5549862 26865020

[27] Ballanger B, Strafella AP, van Eimeren T, et al Serotonin 2A receptors and visual hallucinations in Parkinson disease. Arch Neurol. 2010;67(4):416–421. 10.1001/archneurol.2010.35 20385906

[28] Leptourgos P, Fortier-Davy M, Carhart-Harris R, et al Hallucinations under psychedelics and in the schizophrenia spectrum: An interdisciplinary and multiscale comparison. Schizophr Bull. 2020;46(6):1396–1408. 10.1093/schbul/sbaa117 PMC7707069 32944778

[29] Orlando IF, Shine JM, Robbins TW, Rowe JB, O’Callaghan C. Noradrenergic and cholinergic systems take centre stage in neuropsychiatric diseases of ageing. Neurosci Biobehav Rev. 2023;149:105167. 10.1016/j.neubiorev.2023.105167 37054802

[30] Bohnen NI, Kanel P, Müller MLTM. Molecular imaging of the cholinergic system in Parkinson’s disease. Int Rev Neurobiol. 2018;141:211–250. 10.1016/bs.irn.2018.07.027 PMC6218162 30314597

[31] d’Angremont E, van der Zee S, Slingerland S, et al Cholinergic deficiency in Parkinson’s disease patients with visual hallucinations. Brain. 2024;147(10):3370–3378. 10.1093/brain/awae186 PMC11449127 38864492

[32] Janzen J, van ‘t Ent D, Lemstra AW, Berendse HW, Barkhof F, Foncke EM. The pedunculopontine nucleus is related to visual hallucinations in Parkinson's disease: Preliminary results of a voxel-based morphometry study. J Neurol. 2012;259(1):147–154. 10.1007/s00415-011-6149-z PMC3251778 21717194

[33] Foote SL, Bloom FE, Aston-Jones G. Nucleus locus ceruleus: New evidence of anatomical and physiological specificity. Physiol Rev. 1983;63(3):844–914. 10.1152/physrev.1983.63.3.844 6308694

[34] Benarroch EE . The locus ceruleus norepinephrine system: Functional organization and potential clinical significance. Neurology. 2009;73(20):1699–1704. 10.1212/WNL.0b013e3181c2937c 19917994

[35] Sara SJ . The locus coeruleus and noradrenergic modulation of cognition. Nat Rev Neurosci. 2009;10(3):211–223. 10.1038/nrn2573 19190638

[36] Berridge CW, Waterhouse BD. The locus coeruleus-noradrenergic system: Modulation of behavioral state and state-dependent cognitive processes. Brain Res Brain Res Rev. 2003;42(1):33–84. 10.1016/s0165-0173(03)00143-7 12668290

[37] Waterhouse BD, Navarra RL. The locus coeruleus-norepinephrine system and sensory signal processing: A historical review and current perspectives. Brain Res. 2019;1709:1–15. 10.1016/j.brainres.2018.08.032 30179606

[38] Braak H, Del Tredici K, Rüb U, de Vos RA, Jansen Steur EN, Braak E. Staging of brain pathology related to sporadic Parkinson’s disease. Neurobiol Aging. 2003;24(2):197–211. 10.1016/s0197-4580(02)00065-9 12498954

[39] Halliday G . Substantia nigra and locus coeruleus. In: The human nervous system; 2004.

[40] Del Tredici K, Braak H. Dysfunction of the locus coeruleus-norepinephrine system and related circuitry in Parkinson's disease-related dementia. J Neurol Neurosurg Psychiatry. 2013;84(7):774–783. 10.1136/jnnp-2011-301817 23064099

[41] Tilley BS, Patel SR, Goldfinger MH, Pearce RKB, Gentleman SM. Locus coeruleus pathology indicates a continuum of Lewy body dementia. J Parkinsons Dis. 2021;11(4):1641–1650. 10.3233/JPD-212748 34334423

[42] Ghosh S, Maunsell JHR. Locus coeruleus norepinephrine contributes to visual-spatial attention by selectively enhancing perceptual sensitivity. Neuron. 2024;112(13):2231–2240.e5. 10.1016/j.neuron.2024.04.001 PMC11223979 38701788

[43] Edlow BL, Takahashi E, Wu O, et al Neuroanatomic connectivity of the human ascending arousal system critical to consciousness and its disorders. J Neuropathol Exp Neurol. 2012;71(6):531–546. 10.1097/NEN.0b013e3182588293 PMC3387430 22592840

[44] Casamitjana A, Mancini M, Robinson E, et al A probabilistic histological atlas of the human brain for MRI segmentation. Nature. 2025;648:678–685. 10.1038/s41586-025-09708-2 PMC12711564 41193801

[45] Marek K, Jennings D, Lasch S, et al The Parkinson's progression markers initiative (PPMI). Prog Neurobiol. 2011;95(4):629–635. 10.1016/j.pneurobio.2011.09.005 PMC9014725 21930184

[46] Olchanyi MD, Augustinack J, Haynes RL, et al Automated MRI segmentation of brainstem nuclei critical to consciousness. Hum Brain Mapp. 2025;46:e70357. 10.1002/hbm.70357 PMC12514455 41074651

[47] Padulo C, Sestieri C, Punzi M, et al Atrophy of specific amygdala subfields in subjects converting to mild cognitive impairment. Alzheimers Dement (N Y). 2023;9(4):e12436. 10.1002/trc2.12436 PMC10694338 38053753

[48] Fischl B, Salat DH, Busa E, et al Whole brain segmentation: Automated labeling of neuroanatomical structures in the human brain. Neuron. 2002;33(3):341–355. 10.1016/s0896-6273(02)00569-x 11832223

[49] Billot B, Greve DN, Puonti O, et al SynthSeg: Segmentation of brain MRI scans of any contrast and resolution without retraining. Med Image Anal. 2023;86:102789. 10.1016/j.media.2023.102789 PMC10154424 36857946

[50] Hornung JP . The human raphe nuclei and the serotonergic system. J Chem Neuroanat. 2003;26(4):331–343. 10.1016/j.jchemneu.2003.10.002 14729135

[51] Gantz SC, Ford CP, Morikawa H, Williams JT. Synaptic plasticity in the ventral tegmental area: Cellular mechanisms underlying behaviorally relevant plasticity. Neuron. 2018;97(6):1219–1233.

[52] Bariselli S, Tzanoulinou S, Glangetas C, et al SHANK3 controls maturation of social reward circuits in the VTA. Nat Neurosci. 2016;19(7):926–934. 10.1038/nn.4319 PMC4948673 27273769

[53] Xi M-C, Morales FR, Chase MH. Interactions between GABAergic and cholinergic processes in the nucleus pontis oralis: Neuronal mechanisms controlling active (rapid eye movement) sleep and wakefulness. J Neurosci. 2004;24:10670–10678. 10.1523/JNEUROSCI.1987-04.2004 PMC6730134 15564583

[54] Benarroch EE . Parabrachial nuclear complex: Multiple functions and potential clinical implications. Neurology. 2016;86(7):676–683. 10.1212/WNL.0000000000002393 26791152

[55] Harrison R, Gandhi W, van Reekum CM, Salomons TV. Conditioned pain modulation is associated with heightened connectivity between the periaqueductal grey and cortical regions. Pain Rep. 2022;7(3):e999. 10.1097/PR9.0000000000000999 PMC9084428 35558091

[56] Vázquez-León P, Miranda-Páez A, Valencia-Flores K, Sánchez-Castillo H. Defensive and emotional behavior modulation by serotonin in the periaqueductal gray. Cell Mol Neurobiol. 2023;43:1453–1468. 10.1007/s10571-022-01262-z PMC11412428 35902460

[57] Proulx CD, Hikosaka O, Malinow R. Reward processing by the lateral habenula in normal and depressive behaviors. Nat Neurosci. 2014;17(9):1146–1152. 10.1038/nn.3779 PMC4305435 25157511

[58] Ayyildiz E, Yildiz M, Ozdemir H, Poyraz BÇ. The claustrum and its role in neuropsychiatric disorders: A review. Front Neuroanat. 2023;17:1170217.

[59] Mena-Segovia J, Bolam JP. Rethinking the pedunculopontine nucleus: From cellular organization to function. Neuron. 2017;94(1):7–18. 10.1016/j.neuron.2017.02.027 28384477

[60] Diederich NJ, Goetz CG, Stebbins GT. Repeated visual hallucinations in Parkinson's disease as disturbed external/internal perceptions: Focused review and a new integrative model. Mov Disord. 2005;20(2):130–140. 10.1002/mds.20308 15486924

[61] Hepp DH, Ruiter AM, Galis Y, et al Pedunculopontine cholinergic cell loss in hallucinating Parkinson disease patients but not in dementia with Lewy bodies patients. J Neuropathol Exp Neurol. 2013;72(12):1162–1170. 10.1097/NEN.0000000000000014 24226265

[62] Bohnen NI, Roytman S, Kanel P, et al Progression of regional cortical cholinergic denervation in Parkinson’s disease. Brain Commun. 2022;4(6):fcac320. 10.1093/braincomms/fcac320 PMC9772878 36569603

[63] Huynh B, Fu Y, Kirik D, Shine JM, Halliday GM. Comparison of locus coeruleus pathology with nigral and forebrain pathology in Parkinson’s disease. Mov Disord. 2021;36(9):2085–2093. 10.1002/mds.28615 33899954

[64] Betts MJ, Kirilina E, Otaduy MCG, et al Locus coeruleus imaging as a biomarker for noradrenergic dysfunction in neurodegenerative diseases. Brain. 2019;142(9):2558–2571. 10.1093/brain/awz193 PMC6736046 31327002

[65] Gottesmann C . Noradrenaline involvement in basic and higher integrated REM sleep processes: In memoriam of jean-michel gaillard. Prog Neurobiol. 2008;85(3):237–272. 10.1016/j.pneurobio.2008.04.002 18514380

[66] Vazey EM, Aston-Jones G. The emerging role of norepinephrine in cognitive dysfunctions of Parkinson's disease. Front Behav Neurosci. 2012;6:48. 10.3389/fnbeh.2012.00048 PMC3404393 22848194

[67] Sánchez-Soto M, Bonifazi A, Cai NS, et al Evidence for noncanonical neurotransmitter activation: Norepinephrine as a dopamine D2-like receptor agonist. Mol Pharmacol. 2016;89(4):457–466. 10.1124/mol.115.101808 PMC4809307 26843180

[68] Pasquini J, Ceravolo R, Brooks DJ, Bonuccelli U, Pavese N. Progressive loss of raphe nuclei serotonin transporter in early Parkinson's disease: A longitudinal ^123^I-FP-CIT SPECT study. Parkinsonism Relat Disord. 2020;77:170–175. 10.1016/j.parkreldis.2019.03.025 30981664

[69] Onofrj M, Espay AJ, Bonanni L, Delli Pizzi S, Sensi SL. Hallucinations, somatic-functional disorders of PD-DLB as expressions of thalamic dysfunction. Mov Disord. 2019;34:1100–1111. 10.1002/mds.27781 PMC6707070 31307115

[70] Onofrj M, Russo M, Delli Pizzi S, et al The central role of the thalamus in psychosis, lessons from neurodegenerative diseases and psychedelics. Transl Psychiatry. 2023;13:384. 10.1038/s41398-023-02691-0 PMC10719401 38092757

[71] Guell X, D’Mello AM, Hubbard NA, et al Functional territories of the human dentate nucleus. Cereb Cortex. 2020;30(4):2401–2417. 10.1093/cercor/bhz247 PMC7175000 31701117

[72] Lawn T, Ffytche D. Cerebellar correlates of visual hallucinations in Parkinson's disease and charles bonnet syndrome. Cortex. 2021;135:311–325. 10.1016/j.cortex.2020.10.024 33390262

[73] Samanci B, Tan S, Michielse S, Kuijf ML, Temel Y. The habenula in Parkinson's disease: Anatomy, function, and implications for mood disorders—A narrative review. J Chem Neuroanat. 2024;136:102392. 10.1016/j.jchemneu.2024.102392 38237746

[74] Ozawa M, Shiraishi T, Murakami H, et al Structural MRI study of pareidolia and visual hallucinations in drug-naïve Parkinson’s disease. Sci Rep. 2024;14(1):31293. 10.1038/s41598-024-82707-x PMC11682137 39733021

[75] Meppelink AM, de Jong BM, Teune LK, van Laar T. Regional cortical grey matter loss in Parkinson’s disease without dementia is independent from visual hallucinations. Mov Disord. 2011;26(1):142-147. 10.1002/mds.23375 20922809

[76] Walters RP, Harrison DW, Williamson J, Foster P. Lateralized visual hallucinations: An analysis of affective valence. Appl Neuropsychol. 2006;13(3):160–165. 10.1207/s15324826an1303_3 17361668

[77] Riederer P, Jellinger KA, Kolber P, Hipp G, Sian-Hülsmann J, Krüger R. Lateralisation in Parkinson disease. Cell Tissue Res. 2018;373(1):297–312. 10.1007/s00441-018-2832-z 29656343

[78] Orlhac F, Eertink JJ, Cottereau A-S, et al A guide to ComBat harmonization of imaging biomarkers in multicenter studies. J Nucl Med. 2022;63(2):172–179. 10.2967/jnumed.121.262464 PMC8805779 34531263

