# Supplementary material for: Reduced volume of neuromodulatory brainstem nuclei is associated with early hallucination-related vulnerability in Parkinson’s disease
Source: Brain Commun. 2026 Sep 20;8(5):fcag357. doi: 10.1093/braincomms/fcag357 (PMC13621912; doi:10.1093/braincomms/fcag357)
Supplement: Supplementary file 1 [file fcag357_supplementary_data.docx]

**Table S1. Statistics on demographic, clinical, and neuropsychological outcomes.**

| Variable | | | | | | | PD–mVHs⁻  (N = 577) | | PD–mVHs^+^  (N = 64) | | | χ² | | | p-value | | |
| --- | --- | --- | --- | --- | --- | --- | --- | --- | --- | --- | --- | --- | --- | --- | --- | --- | --- |
| Sex, male/female, n (%) | | | | | | | 357/220 (61.9/38.1) | | 46/18  (71.9/28.1) | | | 2.470 | | | 0.116 | | |
|  | | | | | | | Levene's Test for Equality of Variances | | t-test for equality of means | | | | | | | | |
|  |  |  |  |  |  |  | F | p-value | t | df | p-value | | Mean Difference | SE Difference | | 95% Confidence Interval of the Difference | |
| Variables | Type of equal variance | Group | N | Mean | SD | SE |  |  |  |  |  |  |  |  |  | Inferior | Superior |
| Educational level (years) | Assumed | PD-mVHs⁻ | 577 | 16.1 | 3.1 | 0.1 | 1.306 | 0.254 | 1.623 | 639.0 | 0.105 | | 0.677 | 0.417 | | -0.142 | 1.496 |
|  | Not assumed | PD-mVHs^+^ | 64 | 15.5 | 3.5 | 0.4 |  |  | 1.490 | 74.7 | 0.141 | | 0.677 | 0.454 | | -0.228 | 1.582 |
| Age (years) | Assumed | PD-mVHs⁻ | 577 | 63.1 | 9.2 | 0.4 | 3.449 | 0.064 | 1.218 | 639.0 | 0.224 | | 1.510 | 1.240 | | -0.924 | 3.945 |
|  | Not assumed | PD-mVHs^+^ | 64 | 61.6 | 10.9 | 1.4 |  |  | 1.067 | 73.4 | 0.290 | | 1.510 | 1.416 | | -1.312 | 4.333 |
| MoCA | Assumed | PD-mVHs⁻ | 577 | 27.1 | 2.4 | 0.1 | 3.944 | 0.047 | 1.056 | 639.0 | 0.292 | | 0.340 | 0.322 | | -0.293 | 0.973 |
|  | Not assumed | PD-mVHs^+^ | 64 | 26.7 | 3.0 | 0.4 |  |  | 0.889 | 72.4 | 0.377 | | 0.340 | 0.383 | | -0.423 | 1.103 |
| HVLT-IR | Assumed | PD-mVHs⁻ | 577 | 24.5 | 5.1 | 0.2 | 0.608 | 0.436 | -2.008 | 639.0 | 0.045 | | -1.334 | 0.665 | | -2.639 | -0.029 |
|  | Not assumed | PD-mVHs^+^ | 64 | 25.8 | 4.8 | 0.6 |  |  | -2.109 | 79.7 | 0.038 | | -1.334 | 0.633 | | -2.593 | -0.075 |
| HVLT-DR | Assumed | PD-mVHs⁻ | 577 | 8.3 | 2.8 | 0.1 | 2.823 | 0.093 | -1.787 | 639.0 | 0.074 | | -0.641 | 0.359 | | -1.346 | 0.064 |
|  | Not assumed | PD-mVHs^+^ | 64 | 9.0 | 2.4 | 0.3 |  |  | -1.980 | 82.2 | 0.051 | | -0.641 | 0.324 | | -1.285 | 0.003 |
| HVLT-R | Assumed | PD-mVHs⁻ | 577 | 11.1 | 1.4 | 0.1 | 1.270 | 0.260 | -0.949 | 639.0 | 0.343 | | -0.169 | 0.178 | | -0.517 | 0.180 |
|  | Not assumed | PD-mVHs^+^ | 64 | 11.3 | 1.0 | 0.1 |  |  | -1.239 | 92.9 | 0.219 | | -0.169 | 0.136 | | -0.439 | 0.102 |
| SDM-TOT | Assumed | PD-mVHs⁻ | 577 | 42.5 | 10.4 | 0.4 | 2.510 | 0.114 | 0.172 | 639.0 | 0.864 | | 0.239 | 1.390 | | -2.492 | 2.969 |
|  | Not assumed | PD-mVHs^+^ | 64 | 42.3 | 12.1 | 1.5 |  |  | 0.151 | 73.5 | 0.880 | | 0.239 | 1.578 | | -2.905 | 3.383 |
| LNS | Assumed | PD-mVHs⁻ | 577 | 10.6 | 2.9 | 0.1 | 1.807 | 0.179 | -0.839 | 639.0 | 0.402 | | -0.315 | 0.376 | | -1.053 | 0.422 |
|  | Not assumed | PD-mVHs^+^ | 64 | 10.9 | 2.6 | 0.3 |  |  | -0.911 | 81.2 | 0.365 | | -0.315 | 0.346 | | -1.004 | 0.373 |
| JLO | Assumed | PD-mVHs⁻ | 577 | 25.5 | 4.3 | 0.2 | 0.257 | 0.612 | 0.166 | 639.0 | 0.868 | | 0.095 | 0.570 | | -1.025 | 1.214 |
|  | Not assumed | PD-mVHs^+^ | 64 | 25.4 | 4.3 | 0.5 |  |  | 0.167 | 78.0 | 0.868 | | 0.095 | 0.565 | | -1.030 | 1.219 |
| SF-Animal | Assumed | PD-mVHs⁻ | 577 | 21.8 | 5.5 | 0.2 | 1.135 | 0.287 | 2.391 | 639.0 | 0.017 | | 1.733 | 0.725 | | 0.310 | 3.156 |
|  | Not assumed | PD-mVHs^+^ | 64 | 20.1 | 5.4 | 0.7 |  |  | 2.432 | 78.3 | 0.017 | | 1.733 | 0.713 | | 0.314 | 3.152 |
| STAI-State | Assumed | PD-mVHs⁻ | 577 | 47.8 | 4.8 | 0.2 | 4.177 | 0.041 | 2.355 | 639.0 | 0.019 | | 1.530 | 0.650 | | 0.254 | 2.805 |
|  | Not assumed | PD-mVHs^+^ | 64 | 46.3 | 6.0 | 0.7 |  |  | 1.984 | 72.4 | 0.051 | | 1.530 | 0.771 | | -0.007 | 3.067 |
| STAI-Trait | Assumed | PD-mVHs⁻ | 577 | 46.1 | 4.0 | 0.2 | 2.672 | 0.103 | 0.829 | 639.0 | 0.407 | | 0.448 | 0.540 | | -0.613 | 1.508 |
|  | Not assumed | PD-mVHs^+^ | 64 | 45.7 | 4.9 | 0.6 |  |  | 0.710 | 72.8 | 0.480 | | 0.448 | 0.630 | | -0.809 | 1.704 |
| GDS | Assumed | PD-mVHs⁻ | 577 | 5.4 | 1.4 | 0.1 | 7.965 | 0.005 | -3.363 | 639.0 | 0.001 | | -0.634 | 0.189 | | -1.005 | -0.264 |
|  | Not assumed | PD-mVHs^+^ | 64 | 6.0 | 1.8 | 0.2 |  |  | -2.672 | 71.0 | 0.009 | | -0.634 | 0.237 | | -1.108 | -0.161 |
| HY | Assumed | PD-mVHs⁻ | 577 | 1.7 | 0.5 | 0.0 | 0.276 | 0.599 | -1.582 | 639.0 | 0.114 | | -0.102 | 0.064 | | -0.228 | 0.025 |
|  | Not assumed | PD-mVHs^+^ | 64 | 1.8 | 0.5 | 0.1 |  |  | -1.447 | 74.6 | 0.152 | | -0.102 | 0.070 | | -0.242 | 0.038 |
| NP3TOT | Assumed | PD-mVH⁻ | 575 | 22.6 | 9.8 | 0.4 | 4.434 | 0.036 | -1.844 | 637.0 | 0.066 | | -2.443 | 1.325 | | -5.046 | 0.159 |
|  | Not assumed | PD-mVH^+^ | 64 | 25.0 | 11.9 | 1.5 |  |  | -1.587 | 72.9 | 0.117 | | -2.443 | 1.540 | | -5.513 | 0.626 |
| Disease duration (years) | Assumed | PD-mVHs⁻ | 577 | 1.0 | 1.3 | 0.1 | 29.298 | <0.001 | -3.918 | 639.0 | 0.000 | | -0.693 | 0.177 | | -1.041 | -0.346 |
|  | Not assumed | PD-mVHs^+^ | 64 | 1.7 | 1.9 | 0.2 |  |  | -2.801 | 69.1 | 0.007 | | -0.693 | 0.248 | | -1.187 | -0.200 |
| Age at PD diagnosis (years) | Assumed | PD-mVHs⁻ | 577 | 62.1 | 9.3 | 0.4 | 5.371 | 0.021 | 1.756 | 639.0 | 0.080 | | 2.201 | 1.254 | | -0.260 | 4.663 |
|  | Not assumed | PD-mVHs^+^ | 64 | 59.9 | 11.4 | 1.4 |  |  | 1.487 | 72.5 | 0.141 | | 2.201 | 1.480 | | -0.749 | 5.152 |

*Abbreviations:* GDS = Geriatric Depression Scale; HY = Hoehn and Yahr stage; JLO = Benton Judgment of Line Orientation; LNS = Letter–Number Sequencing; MoCA = Montreal Cognitive Assessment; NP3-TOT = MDS-UPDRS Part III total score; HVLT-DR = Hopkins Verbal Learning Test–Revised – Delayed Recall; HVLT-IR = Hopkins Verbal Learning Test–Revised – Immediate Recall; HVLT-R = Hopkins Verbal Learning Test–Revised – Delayed Recognition; SDM-TOT = Symbol Digit Modalities Test – Total Correct; SF-Animal = Semantic Fluency – Total Number of Animals; STAI-State = State-Trait Anxiety Inventory – State subscale; STAI-Trait = State-Trait Anxiety Inventory – Trait subscale.

**Table S2. Descriptive statistics for the exploratory volumetric analysis of additional brainstem nuclei segmented using the AAN atlas.**

| Structure | Group | N | Mean  (mm³) | SD  (mm³) | SE |
| --- | --- | --- | --- | --- | --- |
| MnR | PD-mVHs⁻ | 577 | 84.85 | 15.18 | 0.63 |
|  | PD-mVHs^+^ | 64 | 84.13 | 15.36 | 1.92 |
| mRt-L | PD-mVHs⁻ | 577 | 246.24 | 31.41 | 1.31 |
|  | PD-mVHs^+^ | 64 | 240.92 | 30.27 | 3.78 |
| mRt-R | PD-mVHs⁻ | 577 | 246.98 | 32.72 | 1.36 |
|  | PD-mVHs^+^ | 64 | 242.30 | 31.34 | 3.92 |
| PAG | PD-mVHs⁻ | 577 | 428.32 | 57.21 | 2.38 |
|  | PD-mVHs^+^ | 64 | 439.13 | 51.52 | 6.44 |
| PBC-L | PD-mVHs⁻ | 577 | 14.35 | 3.96 | 0.16 |
|  | PD-mVHs^+^ | 64 | 14.72 | 3.93 | 0.49 |
| PBC-R | PD-mVHs⁻ | 577 | 16.95 | 4.98 | 0.21 |
|  | PD-mVHs^+^ | 64 | 17.41 | 4.74 | 0.59 |
| PnO-L | PD-mVHs⁻ | 577 | 74.33 | 11.26 | 0.47 |
|  | PD-mVHs^+^ | 64 | 72.27 | 9.79 | 1.22 |
| PnO-R | PD-mVHs⁻ | 577 | 72.22 | 10.83 | 0.45 |
|  | PD-mVHs^+^ | 64 | 70.64 | 9.87 | 1.23 |
| VTA | PD-mVHs⁻ | 577 | 274.47 | 39.47 | 1.64 |
|  | PD-mVHs^+^ | 64 | 270.02 | 37.16 | 4.65 |

*Abbreviations*: L=left; **MnR=median raphe nucleus; mRt=mesencephalic reticular formation; PAG=periaqueductal gray; PBC=parabrachial complex; PnO=pontis oralis; R=right; VTA=Ventral Tegmental Area.**

**Table S3. ANCOVA outputs for the exploratory volumetric analysis of additional brainstem nuclei segmented using the AAN atlas.** ANCOVA models included group and sex as fixed factors, together with the group × sex interaction, and age, education, standardized eTIV, MoCA score, and disease duration as continuous covariates.

| Structure | F | p-value | Power |
| --- | --- | --- | --- |
| MnR | 0.674 | 0.412 | 0.131 |
| mRt-L | 3.686 | 0.055 | 0.488 |
| mRt-R | 2.628 | 0.105 | 0.371 |
| PAG | 0.415 | 0.520 | 0.099 |
| PBC-L | 0.044 | 0.835 | 0.055 |
| PBC-R | 0.023 | 0.880 | 0.053 |
| PnO-L | 3.596 | 0.058 | 0.479 |
| PnO-R | 2.334 | 0.127 | 0.336 |
| VTA | 4.179 | 0.041 | 0.538 |

*Abbreviations:* **MnR=median raphe nucleus; mRt=mesencephalic reticular formation; PAG=periaqueductal gray; PBC=parabrachial complex; PnO=pontis oralis; VTA=Ventral Tegmental Area.**

**Table S4. Descriptive statistics for the exploratory volumetric analysis of subcortical structures segmented using the HistoAtlas integrated within NextBrain.**

| Structure | Group | N | Mean  (mm³) | SD  (mm³) | SE  (mm³) |
| --- | --- | --- | --- | --- | --- |
| alveus-L | PD-mVHs⁻ | 577 | 451 | 58 | 2 |
|  | PD-mVHs^+^ | 64 | 462 | 66 | 8 |
| alveus-R | PD-mVHs⁻ | 577 | 437 | 53 | 2 |
|  | PD-mVHs^+^ | 64 | 438 | 53 | 7 |
| amygdalocortical corticoamygdaloid transition area-L | PD-mVHs⁻ | 577 | 159 | 26 | 1 |
|  | PD-mVHs^+^ | 64 | 158 | 23 | 3 |
| amygdalocortical corticoamygdaloid transition area-R | PD-mVHs⁻ | 577 | 170 | 27 | 1 |
|  | PD-mVHs^+^ | 64 | 171 | 26 | 3 |
| amygdalohippocampal area-L | PD-mVHs⁻ | 577 | 28 | 4 | 0 |
|  | PD-mVHs^+^ | 64 | 28 | 4 | 0 |
| amygdalohippocampal area-R | PD-mVHs⁻ | 577 | 29 | 4 | 0 |
|  | PD-mVHs^+^ | 64 | 30 | 4 | 1 |
| amygdalohippocampal transition area-L | PD-mVHs⁻ | 577 | 16 | 3 | 0 |
|  | PD-mVHs^+^ | 64 | 17 | 4 | 0 |
| amygdalohippocampal transition area-R | PD-mVHs⁻ | 577 | 17 | 3 | 0 |
|  | PD-mVHs^+^ | 64 | 18 | 3 | 0 |
| amygdaloid complex-L | PD-mVHs⁻ | 577 | 84 | 13 | 1 |
|  | PD-mVHs^+^ | 64 | 84 | 14 | 2 |
| amygdaloid complex-R | PD-mVHs⁻ | 577 | 98 | 15 | 1 |
|  | PD-mVHs^+^ | 64 | 99 | 17 | 2 |
| amygdalostriatal transition area-L | PD-mVHs⁻ | 577 | 27 | 6 | 0 |
|  | PD-mVHs^+^ | 64 | 27 | 5 | 1 |
| amygdalostriatal transition area-R | PD-mVHs⁻ | 577 | 33 | 7 | 0 |
|  | PD-mVHs^+^ | 64 | 34 | 6 | 1 |
| anterior amygdaloid area-L | PD-mVHs⁻ | 577 | 142 | 30 | 1 |
|  | PD-mVHs^+^ | 64 | 143 | 29 | 4 |
| anterior amygdaloid area-R | PD-mVHs⁻ | 577 | 150 | 31 | 1 |
|  | PD-mVHs^+^ | 64 | 148 | 28 | 3 |
| anterior commissure-L | PD-mVHs⁻ | 577 | 179 | 38 | 2 |
|  | PD-mVHs^+^ | 64 | 182 | 36 | 5 |
| anterior commissure-R | PD-mVHs⁻ | 577 | 161 | 30 | 1 |
|  | PD-mVHs^+^ | 64 | 163 | 28 | 3 |
| anterior hypothalamic nucleus-L | PD-mVHs⁻ | 577 | 6 | 2 | 0 |
|  | PD-mVHs^+^ | 64 | 6 | 1 | 0 |
| anterior hypothalamic nucleus-R | PD-mVHs⁻ | 577 | 6 | 2 | 0 |
|  | PD-mVHs^+^ | 64 | 6 | 2 | 0 |
| anterior nucleus of pulvinar-L | PD-mVHs⁻ | 577 | 183 | 39 | 2 |
|  | PD-mVHs^+^ | 64 | 189 | 42 | 5 |
| anterior nucleus of pulvinar-R | PD-mVHs⁻ | 577 | 189 | 43 | 2 |
|  | PD-mVHs^+^ | 64 | 195 | 39 | 5 |
| anterior olfactory nucleus-L | PD-mVHs⁻ | 577 | 38 | 6 | 0 |
|  | PD-mVHs^+^ | 64 | 37 | 6 | 1 |
| anterior olfactory nucleus-R | PD-mVHs⁻ | 577 | 36 | 6 | 0 |
|  | PD-mVHs^+^ | 64 | 36 | 6 | 1 |
| anterodorsal nucleus of thalamus-L | PD-mVHs⁻ | 577 | 13 | 6 | 0 |
|  | PD-mVHs^+^ | 64 | 12 | 5 | 1 |
| anterodorsal nucleus of thalamus-R | PD-mVHs⁻ | 577 | 16 | 6 | 0 |
|  | PD-mVHs^+^ | 64 | 16 | 6 | 1 |
| anteromedial nucleus of thalamus-L | PD-mVHs⁻ | 577 | 18 | 7 | 0 |
|  | PD-mVHs^+^ | 64 | 18 | 8 | 1 |
| anteromedial nucleus of thalamus-R | PD-mVHs⁻ | 577 | 18 | 7 | 0 |
|  | PD-mVHs^+^ | 64 | 18 | 8 | 1 |
| anteroventral nucleus of thalamus-L | PD-mVHs⁻ | 577 | 147 | 53 | 2 |
|  | PD-mVHs^+^ | 64 | 139 | 55 | 7 |
| anteroventral nucleus of thalamus-R | PD-mVHs⁻ | 577 | 173 | 61 | 3 |
|  | PD-mVHs^+^ | 64 | 170 | 59 | 7 |
| arcuate nucleus of hypothalamus-L | PD-mVHs⁻ | 577 | 17 | 5 | 0 |
|  | PD-mVHs^+^ | 64 | 18 | 5 | 1 |
| arcuate nucleus of hypothalamus-R | PD-mVHs⁻ | 577 | 20 | 5 | 0 |
|  | PD-mVHs^+^ | 64 | 19 | 5 | 1 |
| basal division of medial mammillary nucleus-L | PD-mVHs⁻ | 577 | 8 | 3 | 0 |
|  | PD-mVHs^+^ | 64 | 9 | 3 | 0 |
| basal division of medial mammillary nucleus-R | PD-mVHs⁻ | 577 | 8 | 3 | 0 |
|  | PD-mVHs^+^ | 64 | 8 | 3 | 0 |
| basal nucleus of meynert-L | PD-mVHs⁻ | 577 | 14 | 2 | 0 |
|  | PD-mVHs^+^ | 64 | 14 | 3 | 0 |
| basal nucleus of meynert-R | PD-mVHs⁻ | 577 | 16 | 2 | 0 |
|  | PD-mVHs^+^ | 64 | 16 | 3 | 0 |
| basal ventral medial nucleus-L | PD-mVHs⁻ | 577 | 6 | 2 | 0 |
|  | PD-mVHs^+^ | 64 | 6 | 1 | 0 |
| basal ventral medial nucleus-R | PD-mVHs⁻ | 577 | 6 | 2 | 0 |
|  | PD-mVHs^+^ | 64 | 6 | 1 | 0 |
| basolateral nucleus basal nucleus-L | PD-mVHs⁻ | 577 | 535 | 80 | 3 |
|  | PD-mVHs^+^ | 64 | 536 | 77 | 10 |
| basolateral nucleus basal nucleus-R | PD-mVHs⁻ | 577 | 570 | 83 | 3 |
|  | PD-mVHs^+^ | 64 | 580 | 80 | 10 |
| bed nucleus of stria terminalis-L | PD-mVHs⁻ | 577 | 103 | 15 | 1 |
|  | PD-mVHs^+^ | 64 | 104 | 15 | 2 |
| bed nucleus of stria terminalis-R | PD-mVHs⁻ | 577 | 92 | 14 | 1 |
|  | PD-mVHs^+^ | 64 | 92 | 14 | 2 |
| body of caudate-L | PD-mVHs⁻ | 577 | 1021 | 177 | 7 |
|  | PD-mVHs^+^ | 64 | 1030 | 170 | 21 |
| body of caudate-R | PD-mVHs⁻ | 577 | 1098 | 179 | 7 |
|  | PD-mVHs^+^ | 64 | 1101 | 165 | 21 |
| caudal division of ventral posterior lateral nucleus-L | PD-mVHs⁻ | 577 | 365 | 91 | 4 |
|  | PD-mVHs^+^ | 64 | 348 | 84 | 11 |
| caudal division of ventral posterior lateral nucleus-R | PD-mVHs⁻ | 577 | 353 | 94 | 4 |
|  | PD-mVHs^+^ | 64 | 336 | 86 | 11 |
| caudal subiculum-L | PD-mVHs⁻ | 577 | 158 | 26 | 1 |
|  | PD-mVHs^+^ | 64 | 156 | 21 | 3 |
| caudal subiculum-R | PD-mVHs⁻ | 577 | 145 | 26 | 1 |
|  | PD-mVHs^+^ | 64 | 148 | 22 | 3 |
| central dorsal nucleus of thalamus-L | PD-mVHs⁻ | 577 | 4 | 2 | 0 |
|  | PD-mVHs^+^ | 64 | 4 | 2 | 0 |
| central dorsal nucleus of thalamus-R | PD-mVHs⁻ | 577 | 4 | 2 | 0 |
|  | PD-mVHs^+^ | 64 | 4 | 2 | 0 |
| central gray of medulla oblongata-L | PD-mVHs⁻ | 577 | 12 | 5 | 0 |
|  | PD-mVHs^+^ | 64 | 12 | 4 | 1 |
| central gray of medulla oblongata-R | PD-mVHs⁻ | 577 | 12 | 4 | 0 |
|  | PD-mVHs^+^ | 64 | 13 | 5 | 1 |
| central medial nucleus of thalamus-L | PD-mVHs⁻ | 577 | 5 | 4 | 0 |
|  | PD-mVHs^+^ | 64 | 5 | 3 | 0 |
| central medial nucleus of thalamus-R | PD-mVHs⁻ | 577 | 5 | 3 | 0 |
|  | PD-mVHs^+^ | 64 | 4 | 2 | 0 |
| central nucleus of inferior colliculus-L | PD-mVHs⁻ | 577 | 97 | 22 | 1 |
|  | PD-mVHs^+^ | 64 | 101 | 24 | 3 |
| central nucleus of inferior colliculus-R | PD-mVHs⁻ | 577 | 95 | 23 | 1 |
|  | PD-mVHs^+^ | 64 | 98 | 24 | 3 |
| central part of medial division of md-L | PD-mVHs⁻ | 577 | 41 | 14 | 1 |
|  | PD-mVHs^+^ | 64 | 41 | 12 | 1 |
| central part of medial division of md-R | PD-mVHs⁻ | 577 | 44 | 14 | 1 |
|  | PD-mVHs^+^ | 64 | 46 | 12 | 2 |
| central part of ventromedial hypothalamic nucleus-L | PD-mVHs⁻ | 577 | 12 | 3 | 0 |
|  | PD-mVHs^+^ | 64 | 12 | 2 | 0 |
| central part of ventromedial hypothalamic nucleus-R | PD-mVHs⁻ | 577 | 13 | 3 | 0 |
|  | PD-mVHs^+^ | 64 | 13 | 3 | 0 |
| centromedian nucleus of thalamus-L | PD-mVHs⁻ | 577 | 20 | 6 | 0 |
|  | PD-mVHs^+^ | 64 | 19 | 5 | 1 |
| centromedian nucleus of thalamus-R | PD-mVHs⁻ | 577 | 19 | 6 | 0 |
|  | PD-mVHs^+^ | 64 | 18 | 5 | 1 |
| claustrum-L | PD-mVHs⁻ | 577 | 703 | 116 | 5 |
|  | PD-mVHs^+^ | 64 | 714 | 116 | 15 |
| claustrum-R | PD-mVHs⁻ | 577 | 717 | 118 | 5 |
|  | PD-mVHs^+^ | 64 | 713 | 116 | 15 |
| core of nucleus accumbens-L | PD-mVHs⁻ | 577 | 502 | 78 | 3 |
|  | PD-mVHs^+^ | 64 | 515 | 61 | 8 |
| core of nucleus accumbens-R | PD-mVHs⁻ | 577 | 503 | 84 | 4 |
|  | PD-mVHs^+^ | 64 | 526 | 82 | 10 |
| densocellular paralamellar division of md-L | PD-mVHs⁻ | 577 | 93 | 30 | 1 |
|  | PD-mVHs^+^ | 64 | 92 | 28 | 3 |
| densocellular paralamellar division of md-R | PD-mVHs⁻ | 577 | 103 | 31 | 1 |
|  | PD-mVHs^+^ | 64 | 100 | 33 | 4 |
| dentate lateral nucleus-L | PD-mVHs⁻ | 577 | 600 | 154 | 6 |
|  | PD-mVHs^+^ | 64 | 527 | 118 | 15 |
| dentate lateral nucleus-R | PD-mVHs⁻ | 577 | 583 | 156 | 6 |
|  | PD-mVHs^+^ | 64 | 555 | 139 | 17 |
| diagonal band-L | PD-mVHs⁻ | 577 | 13 | 2 | 0 |
|  | PD-mVHs^+^ | 64 | 12 | 2 | 0 |
| diagonal band-R | PD-mVHs⁻ | 577 | 11 | 3 | 0 |
|  | PD-mVHs^+^ | 64 | 11 | 2 | 0 |
| diffuse pulvinar nucleus-L | PD-mVHs⁻ | 577 | 14 | 4 | 0 |
|  | PD-mVHs^+^ | 64 | 15 | 4 | 1 |
| diffuse pulvinar nucleus-R | PD-mVHs⁻ | 577 | 14 | 4 | 0 |
|  | PD-mVHs^+^ | 64 | 14 | 4 | 0 |
| dorsal division of central lateral nucleus-L | PD-mVHs⁻ | 577 | 3 | 1 | 0 |
|  | PD-mVHs^+^ | 64 | 3 | 1 | 0 |
| dorsal division of central lateral nucleus-R | PD-mVHs⁻ | 577 | 3 | 1 | 0 |
|  | PD-mVHs^+^ | 64 | 3 | 1 | 0 |
| dorsal lateral geniculate nucleus-L | PD-mVHs⁻ | 577 | 165 | 54 | 2 |
|  | PD-mVHs^+^ | 64 | 166 | 56 | 7 |
| dorsal lateral geniculate nucleus-R | PD-mVHs⁻ | 577 | 151 | 49 | 2 |
|  | PD-mVHs^+^ | 64 | 153 | 59 | 7 |
| dorsal medial geniculate nucleus-L | PD-mVHs⁻ | 577 | 23 | 5 | 0 |
|  | PD-mVHs^+^ | 64 | 23 | 5 | 1 |
| dorsal medial geniculate nucleus-R | PD-mVHs⁻ | 577 | 22 | 6 | 0 |
|  | PD-mVHs^+^ | 64 | 22 | 6 | 1 |
| dorsal part of ventromedial hypothalamic nucleus-L | PD-mVHs⁻ | 577 | 9 | 2 | 0 |
|  | PD-mVHs^+^ | 64 | 10 | 2 | 0 |
| dorsal part of ventromedial hypothalamic nucleus-R | PD-mVHs⁻ | 577 | 10 | 2 | 0 |
|  | PD-mVHs^+^ | 64 | 10 | 2 | 0 |
| dorsal raphe nucleus-L | PD-mVHs⁻ | 577 | 59 | 10 | 0 |
|  | PD-mVHs^+^ | 64 | 59 | 11 | 1 |
| dorsal raphe nucleus-R | PD-mVHs⁻ | 577 | 61 | 12 | 0 |
|  | PD-mVHs^+^ | 64 | 61 | 8 | 1 |
| dorsal subdivision of vlc-L | PD-mVHs⁻ | 577 | 188 | 34 | 1 |
|  | PD-mVHs^+^ | 64 | 183 | 29 | 4 |
| dorsal subdivision of vlc-R | PD-mVHs⁻ | 577 | 192 | 35 | 1 |
|  | PD-mVHs^+^ | 64 | 189 | 33 | 4 |
| dorsomedial hypothalamic nucleus-L | PD-mVHs⁻ | 577 | 6 | 1 | 0 |
|  | PD-mVHs^+^ | 64 | 6 | 1 | 0 |
| dorsomedial hypothalamic nucleus-R | PD-mVHs⁻ | 577 | 6 | 1 | 0 |
|  | PD-mVHs^+^ | 64 | 6 | 1 | 0 |
| external segment of globus pallidus-L | PD-mVHs⁻ | 577 | 976 | 179 | 7 |
|  | PD-mVHs^+^ | 64 | 985 | 181 | 23 |
| external segment of globus pallidus-R | PD-mVHs⁻ | 577 | 1092 | 168 | 7 |
|  | PD-mVHs^+^ | 64 | 1092 | 169 | 21 |
| facial nucleus-L | PD-mVHs⁻ | 577 | 11 | 4 | 0 |
|  | PD-mVHs^+^ | 64 | 11 | 3 | 0 |
| facial nucleus-R | PD-mVHs⁻ | 577 | 11 | 7 | 0 |
|  | PD-mVHs^+^ | 64 | 10 | 2 | 0 |
| fasciculosus nucleus of thalamus-L | PD-mVHs⁻ | 577 | 25 | 8 | 0 |
|  | PD-mVHs^+^ | 64 | 25 | 9 | 1 |
| fasciculosus nucleus of thalamus-R | PD-mVHs⁻ | 577 | 22 | 7 | 0 |
|  | PD-mVHs^+^ | 64 | 23 | 8 | 1 |
| fasciculus retroflexus habenuno interpeduncular tract-L | PD-mVHs⁻ | 577 | 5 | 2 | 0 |
|  | PD-mVHs^+^ | 64 | 5 | 2 | 0 |
| fasciculus retroflexus habenuno interpeduncular tract-R | PD-mVHs⁻ | 577 | 5 | 2 | 0 |
|  | PD-mVHs^+^ | 64 | 5 | 1 | 0 |
| fastigial medial nucleus-L | PD-mVHs⁻ | 577 | 21 | 12 | 1 |
|  | PD-mVHs^+^ | 64 | 20 | 12 | 2 |
| fastigial medial nucleus-R | PD-mVHs⁻ | 577 | 21 | 11 | 0 |
|  | PD-mVHs^+^ | 64 | 18 | 9 | 1 |
| fimbria-L | PD-mVHs⁻ | 577 | 91 | 18 | 1 |
|  | PD-mVHs^+^ | 64 | 90 | 19 | 2 |
| fimbria-R | PD-mVHs⁻ | 577 | 95 | 19 | 1 |
|  | PD-mVHs^+^ | 64 | 93 | 20 | 3 |
| fornix-L | PD-mVHs⁻ | 577 | 285 | 53 | 2 |
|  | PD-mVHs^+^ | 64 | 286 | 58 | 7 |
| fornix-R | PD-mVHs⁻ | 577 | 209 | 42 | 2 |
|  | PD-mVHs^+^ | 64 | 207 | 44 | 6 |
| granular cell layer of pva-L | PD-mVHs⁻ | 577 | 27492 | 2911 | 121 |
|  | PD-mVHs^+^ | 64 | 26923 | 2776 | 347 |
| granular cell layer of pva-R | PD-mVHs⁻ | 577 | 27028 | 2786 | 116 |
|  | PD-mVHs^+^ | 64 | 26615 | 2703 | 338 |
| granular layer of caudal dentate gyrus-L | PD-mVHs⁻ | 577 | 13 | 3 | 0 |
|  | PD-mVHs^+^ | 64 | 13 | 3 | 0 |
| granular layer of caudal dentate gyrus-R | PD-mVHs⁻ | 577 | 13 | 2 | 0 |
|  | PD-mVHs^+^ | 64 | 13 | 2 | 0 |
| granular layer of rostral dentate gyrus-L | PD-mVHs⁻ | 577 | 46 | 9 | 0 |
|  | PD-mVHs^+^ | 64 | 45 | 6 | 1 |
| granular layer of rostral dentate gyrus-R | PD-mVHs⁻ | 577 | 46 | 8 | 0 |
|  | PD-mVHs^+^ | 64 | 45 | 6 | 1 |
| granular layer of uncal dentate gyrus-L | PD-mVHs⁻ | 577 | 5.5 | 0.9 | 0 |
|  | PD-mVHs^+^ | 64 | 5.3 | 0.9 | 0 |
| granular layer of uncal dentate gyrus-R | PD-mVHs⁻ | 577 | 5.4 | 0.9 | 0 |
|  | PD-mVHs^+^ | 64 | 5.3 | 1.0 | 0 |
| habenular commissure-L | PD-mVHs⁻ | 577 | 0.8 | 0.5 | 0 |
|  | PD-mVHs^+^ | 64 | 1.0 | 0.5 | 0 |
| habenular commissure-R | PD-mVHs⁻ | 577 | 0.8 | 0.5 | 0 |
|  | PD-mVHs^+^ | 64 | 0.9 | 0.5 | 0 |
| head of caudate-L | PD-mVHs⁻ | 577 | 2727 | 394 | 16 |
|  | PD-mVHs^+^ | 64 | 2726 | 436 | 54 |
| head of caudate-R | PD-mVHs⁻ | 577 | 2750 | 405 | 17 |
|  | PD-mVHs^+^ | 64 | 2774 | 443 | 55 |
| inferior cerebellar peduncle-L | PD-mVHs⁻ | 577 | 92 | 23 | 1 |
|  | PD-mVHs^+^ | 64 | 91 | 28 | 3 |
| inferior cerebellar peduncle-R | PD-mVHs⁻ | 577 | 90 | 24 | 1 |
|  | PD-mVHs^+^ | 64 | 86 | 21 | 3 |
| inferior nucleus of pulvinar-L | PD-mVHs⁻ | 577 | 54 | 16 | 1 |
|  | PD-mVHs^+^ | 64 | 55 | 16 | 2 |
| inferior nucleus of pulvinar-R | PD-mVHs⁻ | 577 | 48 | 13 | 1 |
|  | PD-mVHs^+^ | 64 | 47 | 13 | 2 |
| inferior olive principal nucleus-L | PD-mVHs⁻ | 577 | 67 | 18 | 1 |
|  | PD-mVHs^+^ | 64 | 67 | 17 | 2 |
| inferior olive principal nucleus-R | PD-mVHs⁻ | 577 | 62 | 19 | 1 |
|  | PD-mVHs^+^ | 64 | 62 | 18 | 2 |
| inferior thalamic peduncle-L | PD-mVHs⁻ | 577 | 16 | 4 | 0 |
|  | PD-mVHs^+^ | 64 | 15 | 4 | 0 |
| inferior thalamic peduncle-R | PD-mVHs⁻ | 577 | 16 | 4 | 0 |
|  | PD-mVHs^+^ | 64 | 16 | 3 | 0 |
| infundibular stalk-L | PD-mVHs⁻ | 577 | 3 | 1 | 0 |
|  | PD-mVHs^+^ | 64 | 3 | 2 | 0 |
| infundibular stalk-R | PD-mVHs⁻ | 577 | 4 | 2 | 0 |
|  | PD-mVHs^+^ | 64 | 4 | 2 | 0 |
| intermediodorsal nucleus of thalamus-L | PD-mVHs⁻ | 577 | 10 | 3 | 0 |
|  | PD-mVHs^+^ | 64 | 11 | 3 | 0 |
| intermediodorsal nucleus of thalamus-R | PD-mVHs⁻ | 577 | 10 | 3 | 0 |
|  | PD-mVHs^+^ | 64 | 10 | 3 | 0 |
| internal segment of globus pallidus-L | PD-mVHs⁻ | 577 | 391 | 78 | 3 |
|  | PD-mVHs^+^ | 64 | 391 | 76 | 10 |
| internal segment of globus pallidus-R | PD-mVHs⁻ | 577 | 462 | 81 | 3 |
|  | PD-mVHs^+^ | 64 | 468 | 80 | 10 |
| interstitial nucleus of cajal-L | PD-mVHs⁻ | 577 | 1 | 0 | 0 |
|  | PD-mVHs^+^ | 64 | 1 | 0 | 0 |
| interstitial nucleus of cajal-R | PD-mVHs⁻ | 577 | 1 | 0 | 0 |
|  | PD-mVHs^+^ | 64 | 1 | 0 | 0 |
| juxtaparaventricular lateral hypothalamic area-L | PD-mVHs⁻ | 577 | 21 | 3 | 0 |
|  | PD-mVHs^+^ | 64 | 21 | 3 | 0 |
| juxtaparaventricular lateral hypothalamic area-R | PD-mVHs⁻ | 577 | 18 | 3 | 0 |
|  | PD-mVHs^+^ | 64 | 18 | 3 | 0 |
| lateral hypothalamic area posterior part-L | PD-mVHs⁻ | 577 | 110 | 19 | 1 |
|  | PD-mVHs^+^ | 64 | 112 | 22 | 3 |
| lateral hypothalamic area posterior part-R | PD-mVHs⁻ | 577 | 100 | 17 | 1 |
|  | PD-mVHs^+^ | 64 | 98 | 17 | 2 |
| lateral division of central lateral nucleus-L | PD-mVHs⁻ | 577 | 75 | 24 | 1 |
|  | PD-mVHs^+^ | 64 | 70 | 14 | 2 |
| lateral division of central lateral nucleus-R | PD-mVHs⁻ | 577 | 76 | 24 | 1 |
|  | PD-mVHs^+^ | 64 | 71 | 17 | 2 |
| lateral division of centromedian nucleus of thalamus-L | PD-mVHs⁻ | 577 | 39 | 13 | 1 |
|  | PD-mVHs^+^ | 64 | 36 | 10 | 1 |
| lateral division of centromedian nucleus of thalamus-R | PD-mVHs⁻ | 577 | 39 | 11 | 0 |
|  | PD-mVHs^+^ | 64 | 38 | 8 | 1 |
| lateral dorsal nucleus of thalamus-L | PD-mVHs⁻ | 577 | 124 | 29 | 1 |
|  | PD-mVHs^+^ | 64 | 123 | 25 | 3 |
| lateral dorsal nucleus of thalamus-R | PD-mVHs⁻ | 577 | 134 | 30 | 1 |
|  | PD-mVHs^+^ | 64 | 134 | 28 | 4 |
| lateral habenular nucleus-L | PD-mVHs⁻ | 577 | 9 | 3 | 0 |
|  | PD-mVHs^+^ | 64 | 10 | 3 | 0 |
| lateral habenular nucleus-R | PD-mVHs⁻ | 577 | 9 | 3 | 0 |
|  | PD-mVHs^+^ | 64 | 9 | 3 | 0 |
| lateral hypothalamic area tuberal part-L | PD-mVHs⁻ | 577 | 44 | 9 | 0 |
|  | PD-mVHs^+^ | 64 | 46 | 8 | 1 |
| lateral hypothalamic area tuberal part-R | PD-mVHs⁻ | 577 | 49 | 9 | 0 |
|  | PD-mVHs^+^ | 64 | 49 | 9 | 1 |
| lateral interpositus emboliform nucleus-L | PD-mVHs⁻ | 577 | 67 | 23 | 1 |
|  | PD-mVHs^+^ | 64 | 62 | 18 | 2 |
| lateral interpositus emboliform nucleus-R | PD-mVHs⁻ | 577 | 61 | 18 | 1 |
|  | PD-mVHs^+^ | 64 | 56 | 17 | 2 |
| lateral mammillary nucleus-L | PD-mVHs⁻ | 577 | 3 | 1 | 0 |
|  | PD-mVHs^+^ | 64 | 3 | 1 | 0 |
| lateral mammillary nucleus-R | PD-mVHs⁻ | 577 | 3 | 1 | 0 |
|  | PD-mVHs^+^ | 64 | 3 | 1 | 0 |
| lateral nucleus-L | PD-mVHs⁻ | 577 | 429 | 74 | 3 |
|  | PD-mVHs^+^ | 64 | 429 | 76 | 10 |
| lateral nucleus of pulvinar-L | PD-mVHs⁻ | 577 | 201 | 42 | 2 |
|  | PD-mVHs^+^ | 64 | 195 | 43 | 5 |
| lateral nucleus of pulvinar-R | PD-mVHs⁻ | 577 | 183 | 37 | 2 |
|  | PD-mVHs^+^ | 64 | 181 | 37 | 5 |
| lateral nucleus-R | PD-mVHs⁻ | 577 | 462 | 71 | 3 |
|  | PD-mVHs^+^ | 64 | 469 | 71 | 9 |
| lateral olfactory area-L | PD-mVHs⁻ | 577 | 89 | 22 | 1 |
|  | PD-mVHs^+^ | 64 | 88 | 19 | 2 |
| lateral olfactory area-R | PD-mVHs⁻ | 577 | 89 | 21 | 1 |
|  | PD-mVHs^+^ | 64 | 86 | 23 | 3 |
| lateral olfactory stria-L | PD-mVHs⁻ | 577 | 11 | 2 | 0 |
|  | PD-mVHs^+^ | 64 | 10 | 2 | 0 |
| lateral olfactory stria-R | PD-mVHs⁻ | 577 | 9 | 3 | 0 |
|  | PD-mVHs^+^ | 64 | 9 | 3 | 0 |
| lateral part of medial mammillary nucleus-L | PD-mVHs⁻ | 577 | 11 | 4 | 0 |
|  | PD-mVHs^+^ | 64 | 12 | 4 | 0 |
| lateral part of medial mammillary nucleus-R | PD-mVHs⁻ | 577 | 11 | 3 | 0 |
|  | PD-mVHs^+^ | 64 | 11 | 4 | 0 |
| lateral posterior nucleus of thalamus-L | PD-mVHs⁻ | 577 | 420 | 69 | 3 |
|  | PD-mVHs^+^ | 64 | 413 | 59 | 7 |
| lateral posterior nucleus of thalamus-R | PD-mVHs⁻ | 577 | 400 | 72 | 3 |
|  | PD-mVHs^+^ | 64 | 389 | 56 | 7 |
| lateral preoptic area-L | PD-mVHs⁻ | 577 | 31 | 6 | 0 |
|  | PD-mVHs^+^ | 64 | 32 | 5 | 1 |
| lateral preoptic area-R | PD-mVHs⁻ | 577 | 28 | 5 | 0 |
|  | PD-mVHs^+^ | 64 | 29 | 5 | 1 |
| lateral subdivision of central nucleus-L | PD-mVHs⁻ | 577 | 7 | 1 | 0 |
|  | PD-mVHs^+^ | 64 | 7 | 1 | 0 |
| lateral subdivision of central nucleus-R | PD-mVHs⁻ | 577 | 8 | 2 | 0 |
|  | PD-mVHs^+^ | 64 | 8 | 2 | 0 |
| lateral tuberal nuclei-L | PD-mVHs⁻ | 577 | 7 | 2 | 0 |
|  | PD-mVHs^+^ | 64 | 7 | 2 | 0 |
| lateral tuberal nuclei-R | PD-mVHs⁻ | 577 | 7 | 2 | 0 |
|  | PD-mVHs^+^ | 64 | 7 | 1 | 0 |
| laterodorsal portion of sth-L | PD-mVHs⁻ | 577 | 34 | 12 | 1 |
|  | PD-mVHs^+^ | 64 | 36 | 11 | 1 |
| laterodorsal portion of sth-R | PD-mVHs⁻ | 577 | 34 | 11 | 0 |
|  | PD-mVHs^+^ | 64 | 35 | 9 | 1 |
| lateroventral portion of sth-L | PD-mVHs⁻ | 577 | 36 | 9 | 0 |
|  | PD-mVHs^+^ | 64 | 37 | 9 | 1 |
| lateroventral portion of sth-R | PD-mVHs⁻ | 577 | 37 | 7 | 0 |
|  | PD-mVHs^+^ | 64 | 38 | 6 | 1 |
| layer III of piriform cortex-L | PD-mVHs⁻ | 577 | 16 | 4 | 0 |
|  | PD-mVHs^+^ | 64 | 16 | 4 | 1 |
| layer III of piriform cortex-R | PD-mVHs⁻ | 577 | 18 | 5 | 0 |
|  | PD-mVHs^+^ | 64 | 18 | 7 | 1 |
| lenticular fasciculus-L | PD-mVHs⁻ | 577 | 37 | 17 | 1 |
|  | PD-mVHs^+^ | 64 | 35 | 15 | 2 |
| lenticular fasciculus-R | PD-mVHs⁻ | 577 | 37 | 16 | 1 |
|  | PD-mVHs^+^ | 64 | 35 | 16 | 2 |
| limitans nucleus-L | PD-mVHs⁻ | 577 | 26 | 6 | 0 |
|  | PD-mVHs^+^ | 64 | 26 | 6 | 1 |
| limitans nucleus-R | PD-mVHs⁻ | 577 | 24 | 7 | 0 |
|  | PD-mVHs^+^ | 64 | 25 | 6 | 1 |
| magnocellular medial division of md-L | PD-mVHs⁻ | 577 | 367 | 78 | 3 |
|  | PD-mVHs^+^ | 64 | 362 | 67 | 8 |
| magnocellular medial division of md-R | PD-mVHs⁻ | 577 | 410 | 81 | 3 |
|  | PD-mVHs^+^ | 64 | 399 | 68 | 9 |
| magnocellular medial nucleus-L | PD-mVHs⁻ | 577 | 4 | 1 | 0 |
|  | PD-mVHs^+^ | 64 | 4 | 1 | 0 |
| magnocellular medial nucleus-R | PD-mVHs⁻ | 577 | 4 | 1 | 0 |
|  | PD-mVHs^+^ | 64 | 4 | 1 | 0 |
| magnocellular division of reticular nucleus-L | PD-mVHs⁻ | 577 | 18 | 4 | 0 |
|  | PD-mVHs^+^ | 64 | 17 | 3 | 0 |
| magnocellular division of reticular nucleus-R | PD-mVHs⁻ | 577 | 19 | 4 | 0 |
|  | PD-mVHs^+^ | 64 | 19 | 3 | 0 |
| magnocellular division of va-L | PD-mVHs⁻ | 577 | 313 | 69 | 3 |
|  | PD-mVHs^+^ | 64 | 321 | 73 | 9 |
| magnocellular division of va-R | PD-mVHs⁻ | 577 | 346 | 79 | 3 |
|  | PD-mVHs^+^ | 64 | 351 | 86 | 11 |
| mammillary peduncle-L | PD-mVHs⁻ | 577 | 21 | 8 | 0 |
|  | PD-mVHs^+^ | 64 | 23 | 8 | 1 |
| mammillary peduncle-R | PD-mVHs⁻ | 577 | 21 | 7 | 0 |
|  | PD-mVHs^+^ | 64 | 21 | 8 | 1 |
| mammillothalamic tract-L | PD-mVHs⁻ | 577 | 57 | 12 | 1 |
|  | PD-mVHs^+^ | 64 | 57 | 11 | 1 |
| mammillothalamic tract-R | PD-mVHs⁻ | 577 | 60 | 12 | 1 |
|  | PD-mVHs^+^ | 64 | 61 | 10 | 1 |
| medial corticohypothalamic tract-L | PD-mVHs⁻ | 577 | 1 | 0 | 0 |
|  | PD-mVHs^+^ | 64 | 1 | 0 | 0 |
| medial corticohypothalamic tract-R | PD-mVHs⁻ | 577 | 1 | 0 | 0 |
|  | PD-mVHs^+^ | 64 | 1 | 0 | 0 |
| medial division of centromedian nucleus of thalamus-L | PD-mVHs⁻ | 577 | 83 | 20 | 1 |
|  | PD-mVHs^+^ | 64 | 80 | 19 | 2 |
| medial division of centromedian nucleus of thalamus-R | PD-mVHs⁻ | 577 | 86 | 19 | 1 |
|  | PD-mVHs^+^ | 64 | 84 | 17 | 2 |
| medial habenular nucleus-L | PD-mVHs⁻ | 577 | 2 | 1 | 0 |
|  | PD-mVHs^+^ | 64 | 2 | 1 | 0 |
| medial habenular nucleus-R | PD-mVHs⁻ | 577 | 3 | 1 | 0 |
|  | PD-mVHs^+^ | 64 | 2 | 1 | 0 |
| medial interpositus globose nucleus-L | PD-mVHs⁻ | 577 | 29 | 7 | 0 |
|  | PD-mVHs^+^ | 64 | 27 | 6 | 1 |
| medial interpositus globose nucleus-R | PD-mVHs⁻ | 577 | 26 | 6 | 0 |
|  | PD-mVHs^+^ | 64 | 24 | 7 | 1 |
| medial nucleus of pulvinar-L | PD-mVHs⁻ | 577 | 673 | 133 | 6 |
|  | PD-mVHs^+^ | 64 | 686 | 127 | 16 |
| medial nucleus of pulvinar-R | PD-mVHs⁻ | 577 | 643 | 121 | 5 |
|  | PD-mVHs^+^ | 64 | 648 | 102 | 13 |
| medial part of medial mammillary nucleus-L | PD-mVHs⁻ | 577 | 13 | 5 | 0 |
|  | PD-mVHs^+^ | 64 | 14 | 4 | 1 |
| medial part of medial mammillary nucleus-R | PD-mVHs⁻ | 577 | 13 | 4 | 0 |
|  | PD-mVHs^+^ | 64 | 13 | 5 | 1 |
| medial portion of sth-L | PD-mVHs⁻ | 577 | 26 | 6 | 0 |
|  | PD-mVHs^+^ | 64 | 26 | 6 | 1 |
| medial portion of sth-R | PD-mVHs⁻ | 577 | 26 | 5 | 0 |
|  | PD-mVHs^+^ | 64 | 26 | 4 | 1 |
| medial preoptic nucleus-L | PD-mVHs⁻ | 577 | 10 | 3 | 0 |
|  | PD-mVHs^+^ | 64 | 11 | 2 | 0 |
| medial preoptic nucleus-R | PD-mVHs⁻ | 577 | 9 | 2 | 0 |
|  | PD-mVHs^+^ | 64 | 10 | 2 | 0 |
| medial septal nucleus-L | PD-mVHs⁻ | 577 | 108 | 21 | 1 |
|  | PD-mVHs^+^ | 64 | 108 | 20 | 3 |
| medial septal nucleus-R | PD-mVHs⁻ | 577 | 80 | 16 | 1 |
|  | PD-mVHs^+^ | 64 | 79 | 17 | 2 |
| medial subdivision of central nucleus-L | PD-mVHs⁻ | 577 | 22 | 4 | 0 |
|  | PD-mVHs^+^ | 64 | 21 | 4 | 1 |
| medial subdivision of central nucleus-R | PD-mVHs⁻ | 577 | 25 | 5 | 0 |
|  | PD-mVHs^+^ | 64 | 26 | 5 | 1 |
| medial subdivision of vlc area x-L | PD-mVHs⁻ | 577 | 113 | 35 | 2 |
|  | PD-mVHs^+^ | 64 | 111 | 34 | 4 |
| medial subdivision of vlc area x-R | PD-mVHs⁻ | 577 | 127 | 38 | 2 |
|  | PD-mVHs^+^ | 64 | 123 | 35 | 4 |
| median preoptic nucleus-L | PD-mVHs⁻ | 577 | 5 | 1 | 0 |
|  | PD-mVHs^+^ | 64 | 5 | 1 | 0 |
| median preoptic nucleus-R | PD-mVHs⁻ | 577 | 5 | 1 | 0 |
|  | PD-mVHs^+^ | 64 | 5 | 1 | 0 |
| midbrain mesencephalon-L | PD-mVHs⁻ | 577 | 2003 | 240 | 10 |
|  | PD-mVHs^+^ | 64 | 1973 | 258 | 32 |
| midbrain mesencephalon-R | PD-mVHs⁻ | 577 | 2059 | 246 | 10 |
|  | PD-mVHs^+^ | 64 | 2038 | 210 | 26 |
| molecular layer of caudal dentate gyrus-L | PD-mVHs⁻ | 577 | 52 | 10 | 0 |
|  | PD-mVHs^+^ | 64 | 53 | 9 | 1 |
| molecular layer of caudal dentate gyrus-R | PD-mVHs⁻ | 577 | 51 | 9 | 0 |
|  | PD-mVHs^+^ | 64 | 53 | 8 | 1 |
| molecular layer of pva-L | PD-mVHs⁻ | 577 | 28273 | 3022 | 126 |
|  | PD-mVHs^+^ | 64 | 27696 | 2940 | 368 |
| molecular layer of pva-R | PD-mVHs⁻ | 577 | 27719 | 2882 | 120 |
|  | PD-mVHs^+^ | 64 | 27292 | 2805 | 351 |
| molecular layer of rostral dentate gyrus-L | PD-mVHs⁻ | 577 | 222 | 38 | 2 |
|  | PD-mVHs^+^ | 64 | 219 | 29 | 4 |
| molecular layer of rostral dentate gyrus-R | PD-mVHs⁻ | 577 | 220 | 34 | 1 |
|  | PD-mVHs^+^ | 64 | 218 | 28 | 3 |
| molecular layer of uncal dentate gyrus-L | PD-mVHs⁻ | 577 | 30 | 5 | 0 |
|  | PD-mVHs^+^ | 64 | 29 | 5 | 1 |
| molecular layer of uncal dentate gyrus-R | PD-mVHs⁻ | 577 | 29 | 5 | 0 |
|  | PD-mVHs^+^ | 64 | 29 | 5 | 1 |
| multiform lateral division of md-L | PD-mVHs⁻ | 577 | 240 | 48 | 2 |
|  | PD-mVHs^+^ | 64 | 241 | 47 | 6 |
| multiform lateral division of md-R | PD-mVHs⁻ | 577 | 259 | 55 | 2 |
|  | PD-mVHs^+^ | 64 | 259 | 56 | 7 |
| myelencephalon medulla oblongata-L | PD-mVHs⁻ | 577 | 1407 | 198 | 8 |
|  | PD-mVHs^+^ | 64 | 1399 | 203 | 25 |
| myelencephalon medulla oblongata-R | PD-mVHs⁻ | 577 | 1408 | 201 | 8 |
|  | PD-mVHs^+^ | 64 | 1420 | 230 | 29 |
| nucleus of the field of forel-L | PD-mVHs⁻ | 577 | 5 | 2 | 0 |
|  | PD-mVHs^+^ | 64 | 5 | 2 | 0 |
| nucleus of the field of forel-R | PD-mVHs⁻ | 577 | 5 | 2 | 0 |
|  | PD-mVHs^+^ | 64 | 5 | 2 | 0 |
| olfactory tubercle-L | PD-mVHs⁻ | 577 | 3 | 1 | 0 |
|  | PD-mVHs^+^ | 64 | 3 | 1 | 0 |
| olfactory tubercle-R | PD-mVHs⁻ | 577 | 3 | 1 | 0 |
|  | PD-mVHs^+^ | 64 | 3 | 1 | 0 |
| optic chiasm-L | PD-mVHs⁻ | 577 | 4 | 2 | 0 |
|  | PD-mVHs^+^ | 64 | 4 | 2 | 0 |
| optic chiasm-R | PD-mVHs⁻ | 577 | 6 | 2 | 0 |
|  | PD-mVHs^+^ | 64 | 5 | 2 | 0 |
| optic nerve-L | PD-mVHs⁻ | 577 | 0 | 0 | 0 |
|  | PD-mVHs^+^ | 64 | 0 | 0 | 0 |
| optic nerve-R | PD-mVHs⁻ | 577 | 0 | 0 | 0 |
|  | PD-mVHs^+^ | 64 | 0 | 0 | 0 |
| optic tract-L | PD-mVHs⁻ | 577 | 152 | 24 | 1 |
|  | PD-mVHs^+^ | 64 | 155 | 22 | 3 |
| optic tract-R | PD-mVHs⁻ | 577 | 155 | 24 | 1 |
|  | PD-mVHs^+^ | 64 | 160 | 23 | 3 |
| pallidohypothalamic area-L | PD-mVHs⁻ | 577 | 46 | 10 | 0 |
|  | PD-mVHs^+^ | 64 | 47 | 10 | 1 |
| pallidohypothalamic area-R | PD-mVHs⁻ | 577 | 48 | 9 | 0 |
|  | PD-mVHs^+^ | 64 | 48 | 8 | 1 |
| paracentral nucleus of thalamus-L | PD-mVHs⁻ | 577 | 85 | 17 | 1 |
|  | PD-mVHs^+^ | 64 | 84 | 14 | 2 |
| paracentral nucleus of thalamus-R | PD-mVHs⁻ | 577 | 95 | 20 | 1 |
|  | PD-mVHs^+^ | 64 | 94 | 16 | 2 |
| parafascicular nucleus of thalamus-L | PD-mVHs⁻ | 577 | 32 | 8 | 0 |
|  | PD-mVHs^+^ | 64 | 32 | 7 | 1 |
| parafascicular nucleus of thalamus-R | PD-mVHs⁻ | 577 | 33 | 7 | 0 |
|  | PD-mVHs^+^ | 64 | 33 | 8 | 1 |
| paralaminar nucleus-L | PD-mVHs⁻ | 577 | 13 | 2 | 0 |
|  | PD-mVHs^+^ | 64 | 13 | 2 | 0 |
| paralaminar nucleus-R | PD-mVHs⁻ | 577 | 13 | 2 | 0 |
|  | PD-mVHs^+^ | 64 | 13 | 2 | 0 |
| parasubthalamic nucleus-L | PD-mVHs⁻ | 577 | 0 | 1 | 0 |
|  | PD-mVHs^+^ | 64 | 0 | 0 | 0 |
| parasubthalamic nucleus-R | PD-mVHs⁻ | 577 | 0 | 1 | 0 |
|  | PD-mVHs^+^ | 64 | 0 | 0 | 0 |
| parataenial nucleus of thalamus-L | PD-mVHs⁻ | 577 | 10 | 4 | 0 |
|  | PD-mVHs^+^ | 64 | 10 | 4 | 1 |
| parataenial nucleus of thalamus-R | PD-mVHs⁻ | 577 | 11 | 4 | 0 |
|  | PD-mVHs^+^ | 64 | 11 | 4 | 1 |
| paraventricular nucleus-L | PD-mVHs⁻ | 577 | 96 | 20 | 1 |
|  | PD-mVHs^+^ | 64 | 94 | 22 | 3 |
| paraventricular nucleus of hypothalamus-L | PD-mVHs⁻ | 577 | 23 | 5 | 0 |
|  | PD-mVHs^+^ | 64 | 25 | 5 | 1 |
| paraventricular nucleus of hypothalamus-R | PD-mVHs⁻ | 577 | 21 | 4 | 0 |
|  | PD-mVHs^+^ | 64 | 22 | 5 | 1 |
| paraventricular nucleus-R | PD-mVHs⁻ | 577 | 94 | 20 | 1 |
|  | PD-mVHs^+^ | 64 | 93 | 20 | 2 |
| parvocellular central division of md-L | PD-mVHs⁻ | 577 | 453 | 78 | 3 |
|  | PD-mVHs^+^ | 64 | 455 | 71 | 9 |
| parvocellular central division of md-R | PD-mVHs⁻ | 577 | 484 | 86 | 4 |
|  | PD-mVHs^+^ | 64 | 479 | 79 | 10 |
| parvocellular division of retic nucl periret nucleus-L | PD-mVHs⁻ | 577 | 18 | 6 | 0 |
|  | PD-mVHs^+^ | 64 | 17 | 4 | 1 |
| parvocellular division of retic nucl periret nucleus-R | PD-mVHs⁻ | 577 | 19 | 7 | 0 |
|  | PD-mVHs^+^ | 64 | 19 | 5 | 1 |
| parvocellular division of va-L | PD-mVHs⁻ | 577 | 635 | 120 | 5 |
|  | PD-mVHs^+^ | 64 | 628 | 119 | 15 |
| parvocellular division of va-R | PD-mVHs⁻ | 577 | 728 | 141 | 6 |
|  | PD-mVHs^+^ | 64 | 726 | 127 | 16 |
| parvocellular division of vpm-L | PD-mVHs⁻ | 577 | 11 | 4 | 0 |
|  | PD-mVHs^+^ | 64 | 12 | 4 | 1 |
| parvocellular division of vpm-R | PD-mVHs⁻ | 577 | 12 | 5 | 0 |
|  | PD-mVHs^+^ | 64 | 11 | 4 | 1 |
| periaqueductal gray substance-L | PD-mVHs⁻ | 577 | 250 | 43 | 2 |
|  | PD-mVHs^+^ | 64 | 252 | 46 | 6 |
| periaqueductal gray substance-R | PD-mVHs⁻ | 577 | 268 | 45 | 2 |
|  | PD-mVHs^+^ | 64 | 273 | 47 | 6 |
| peripeduncular nucleus-L | PD-mVHs⁻ | 577 | 11 | 3 | 0 |
|  | PD-mVHs^+^ | 64 | 11 | 3 | 0 |
| peripeduncular nucleus-R | PD-mVHs⁻ | 577 | 10 | 3 | 0 |
|  | PD-mVHs^+^ | 64 | 10 | 3 | 0 |
| periventricular area of thalamus-L | PD-mVHs⁻ | 577 | 38 | 8 | 0 |
|  | PD-mVHs^+^ | 64 | 39 | 6 | 1 |
| periventricular area of thalamus-R | PD-mVHs⁻ | 577 | 38 | 7 | 0 |
|  | PD-mVHs^+^ | 64 | 38 | 6 | 1 |
| periventricular nucleus supraoptic portion-L | PD-mVHs⁻ | 577 | 5 | 2 | 0 |
|  | PD-mVHs^+^ | 64 | 5 | 2 | 0 |
| periventricular nucleus supraoptic portion-R | PD-mVHs⁻ | 577 | 6 | 2 | 0 |
|  | PD-mVHs^+^ | 64 | 6 | 2 | 0 |
| periventricular nucleus tuberal portion-L | PD-mVHs⁻ | 577 | 8 | 2 | 0 |
|  | PD-mVHs^+^ | 64 | 8 | 2 | 0 |
| periventricular nucleus tuberal portion-R | PD-mVHs⁻ | 577 | 9 | 2 | 0 |
|  | PD-mVHs^+^ | 64 | 9 | 2 | 0 |
| pineal body-L | PD-mVHs⁻ | 577 | 0 | 0 | 0 |
|  | PD-mVHs^+^ | 64 | 0 | 0 | 0 |
| pineal body-R | PD-mVHs⁻ | 577 | 0 | 0 | 0 |
|  | PD-mVHs^+^ | 64 | 0 | 0 | 0 |
| polyform layer of caudal dentate gyrus-L | PD-mVHs⁻ | 577 | 26 | 5 | 0 |
|  | PD-mVHs^+^ | 64 | 27 | 5 | 1 |
| polyform layer of caudal dentate gyrus-R | PD-mVHs⁻ | 577 | 26 | 5 | 0 |
|  | PD-mVHs^+^ | 64 | 27 | 5 | 1 |
| polyform layer of rostral dentate gyrus-L | PD-mVHs⁻ | 577 | 146 | 31 | 1 |
|  | PD-mVHs^+^ | 64 | 143 | 25 | 3 |
| polyform layer of rostral dentate gyrus-R | PD-mVHs⁻ | 577 | 148 | 29 | 1 |
|  | PD-mVHs^+^ | 64 | 147 | 24 | 3 |
| polyform layer of uncal dentate gyrus-L | PD-mVHs⁻ | 577 | 17 | 3 | 0 |
|  | PD-mVHs^+^ | 64 | 17 | 3 | 0 |
| polyform layer of uncal dentate gyrus-R | PD-mVHs⁻ | 577 | 17 | 3 | 0 |
|  | PD-mVHs^+^ | 64 | 16 | 3 | 0 |
| pons-L | PD-mVHs⁻ | 577 | 4348 | 658 | 27 |
|  | PD-mVHs^+^ | 64 | 4304 | 586 | 73 |
| pons-R | PD-mVHs⁻ | 577 | 4506 | 738 | 31 |
|  | PD-mVHs^+^ | 64 | 4501 | 660 | 83 |
| pontine nucleus-L | PD-mVHs⁻ | 577 | 1473 | 223 | 9 |
|  | PD-mVHs^+^ | 64 | 1523 | 298 | 37 |
| pontine nucleus-R | PD-mVHs⁻ | 577 | 1509 | 241 | 10 |
|  | PD-mVHs^+^ | 64 | 1492 | 214 | 27 |
| posterior hypothalamic nucleus-L | PD-mVHs⁻ | 577 | 130 | 23 | 1 |
|  | PD-mVHs^+^ | 64 | 129 | 19 | 2 |
| posterior hypothalamic nucleus-R | PD-mVHs⁻ | 577 | 126 | 22 | 1 |
|  | PD-mVHs^+^ | 64 | 122 | 24 | 3 |
| posterior nucleus of thalamus-L | PD-mVHs⁻ | 577 | 85 | 18 | 1 |
|  | PD-mVHs^+^ | 64 | 85 | 18 | 2 |
| posterior nucleus of thalamus-R | PD-mVHs⁻ | 577 | 82 | 17 | 1 |
|  | PD-mVHs^+^ | 64 | 81 | 18 | 2 |
| posteroventral putamen-L | PD-mVHs⁻ | 577 | 40 | 10 | 0 |
|  | PD-mVHs^+^ | 64 | 43 | 7 | 1 |
| posteroventral putamen-R | PD-mVHs⁻ | 577 | 45 | 11 | 1 |
|  | PD-mVHs^+^ | 64 | 47 | 10 | 1 |
| preoptic region of hth-L | PD-mVHs⁻ | 577 | 35 | 7 | 0 |
|  | PD-mVHs^+^ | 64 | 37 | 6 | 1 |
| preoptic region of hth-R | PD-mVHs⁻ | 577 | 32 | 6 | 0 |
|  | PD-mVHs^+^ | 64 | 33 | 6 | 1 |
| pretectal region-L | PD-mVHs⁻ | 577 | 54 | 9 | 0 |
|  | PD-mVHs^+^ | 64 | 54 | 10 | 1 |
| pretectal region-R | PD-mVHs⁻ | 577 | 53 | 10 | 0 |
|  | PD-mVHs^+^ | 64 | 54 | 9 | 1 |
| pulvinar of thalamus-L | PD-mVHs⁻ | 577 | 40 | 7 | 0 |
|  | PD-mVHs^+^ | 64 | 40 | 7 | 1 |
| pulvinar of thalamus-R | PD-mVHs⁻ | 577 | 41 | 7 | 0 |
|  | PD-mVHs^+^ | 64 | 41 | 7 | 1 |
| putamen-L | PD-mVHs⁻ | 577 | 5019 | 645 | 27 |
|  | PD-mVHs^+^ | 64 | 5023 | 509 | 64 |
| putamen-R | PD-mVHs⁻ | 577 | 4845 | 645 | 27 |
|  | PD-mVHs^+^ | 64 | 4845 | 540 | 67 |
| pyramidal cells of caudal ca4-L | PD-mVHs⁻ | 577 | 20 | 5 | 0 |
|  | PD-mVHs^+^ | 64 | 21 | 5 | 1 |
| pyramidal cells of caudal ca4-R | PD-mVHs⁻ | 577 | 20 | 4 | 0 |
|  | PD-mVHs^+^ | 64 | 21 | 4 | 1 |
| pyramidal cells of rostral ca4-L | PD-mVHs⁻ | 577 | 91 | 23 | 1 |
|  | PD-mVHs^+^ | 64 | 90 | 16 | 2 |
| pyramidal cells of rostral ca4-R | PD-mVHs⁻ | 577 | 92 | 21 | 1 |
|  | PD-mVHs^+^ | 64 | 89 | 18 | 2 |
| pyramidal cells of uncal ca4-L | PD-mVHs⁻ | 577 | 6 | 1 | 0 |
|  | PD-mVHs^+^ | 64 | 6 | 1 | 0 |
| pyramidal cells of uncal ca4-R | PD-mVHs⁻ | 577 | 6 | 1 | 0 |
|  | PD-mVHs^+^ | 64 | 6 | 1 | 0 |
| red nucleus-L | PD-mVHs⁻ | 577 | 141 | 37 | 2 |
|  | PD-mVHs^+^ | 64 | 139 | 33 | 4 |
| red nucleus-R | PD-mVHs⁻ | 577 | 148 | 32 | 1 |
|  | PD-mVHs^+^ | 64 | 146 | 30 | 4 |
| reticular nucleus of thalamus-L | PD-mVHs⁻ | 577 | 325 | 97 | 4 |
|  | PD-mVHs^+^ | 64 | 310 | 64 | 8 |
| reticular nucleus of thalamus-R | PD-mVHs⁻ | 577 | 321 | 101 | 4 |
|  | PD-mVHs^+^ | 64 | 302 | 65 | 8 |
| reuniens nucleus medioventral nucleus of thalamus-L | PD-mVHs⁻ | 577 | 44 | 9 | 0 |
|  | PD-mVHs^+^ | 64 | 45 | 10 | 1 |
| reuniens nucleus medioventral nucleus of thalamus-R | PD-mVHs⁻ | 577 | 43 | 9 | 0 |
|  | PD-mVHs^+^ | 64 | 42 | 9 | 1 |
| rhomboid central nucleus of thalamus-L | PD-mVHs⁻ | 577 | 7 | 2 | 0 |
|  | PD-mVHs^+^ | 64 | 7 | 2 | 0 |
| rhomboid central nucleus of thalamus-R | PD-mVHs⁻ | 577 | 6 | 2 | 0 |
|  | PD-mVHs^+^ | 64 | 6 | 2 | 0 |
| rostral division of ventral posterior lateral nucleus-L | PD-mVHs⁻ | 577 | 196 | 48 | 2 |
|  | PD-mVHs^+^ | 64 | 191 | 50 | 6 |
| rostral division of ventral posterior lateral nucleus-R | PD-mVHs⁻ | 577 | 207 | 50 | 2 |
|  | PD-mVHs^+^ | 64 | 200 | 47 | 6 |
| rostral division of vl-L | PD-mVHs⁻ | 577 | 178 | 37 | 2 |
|  | PD-mVHs^+^ | 64 | 176 | 35 | 4 |
| rostral division of vl-R | PD-mVHs⁻ | 577 | 200 | 43 | 2 |
|  | PD-mVHs^+^ | 64 | 198 | 41 | 5 |
| rostral subdivision of medial nucleus-L | PD-mVHs⁻ | 577 | 27 | 5 | 0 |
|  | PD-mVHs^+^ | 64 | 27 | 5 | 1 |
| rostral subdivision of medial nucleus-R | PD-mVHs⁻ | 577 | 32 | 6 | 0 |
|  | PD-mVHs^+^ | 64 | 33 | 6 | 1 |
| rostral subiculum-L | PD-mVHs⁻ | 577 | 747 | 88 | 4 |
|  | PD-mVHs^+^ | 64 | 745 | 100 | 13 |
| rostral subiculum-R | PD-mVHs⁻ | 577 | 681 | 79 | 3 |
|  | PD-mVHs^+^ | 64 | 676 | 67 | 8 |
| septum pellucidum-L | PD-mVHs⁻ | 577 | 10 | 3 | 0 |
|  | PD-mVHs^+^ | 64 | 10 | 3 | 0 |
| septum pellucidum-R | PD-mVHs⁻ | 577 | 10 | 3 | 0 |
|  | PD-mVHs^+^ | 64 | 10 | 3 | 0 |
| solitary tract-L | PD-mVHs⁻ | 577 | 0 | 0 | 0 |
|  | PD-mVHs^+^ | 64 | 0 | 0 | 0 |
| solitary tract-R | PD-mVHs⁻ | 577 | 0 | 0 | 0 |
|  | PD-mVHs^+^ | 64 | 0 | 0 | 0 |
| stratum lacunosum moleculare of caudal ca1-L | PD-mVHs⁻ | 577 | 52 | 11 | 0 |
|  | PD-mVHs^+^ | 64 | 52 | 11 | 1 |
| stratum lacunosum moleculare of caudal ca1-R | PD-mVHs⁻ | 577 | 52 | 10 | 0 |
|  | PD-mVHs^+^ | 64 | 55 | 11 | 1 |
| stratum lacunosum moleculare of caudal ca2-L | PD-mVHs⁻ | 577 | 7 | 2 | 0 |
|  | PD-mVHs^+^ | 64 | 8 | 2 | 0 |
| stratum lacunosum moleculare of caudal ca2-R | PD-mVHs⁻ | 577 | 8 | 2 | 0 |
|  | PD-mVHs^+^ | 64 | 9 | 2 | 0 |
| stratum lacunosum moleculare of caudal ca3-L | PD-mVHs⁻ | 577 | 8 | 2 | 0 |
|  | PD-mVHs^+^ | 64 | 8 | 2 | 0 |
| stratum lacunosum moleculare of caudal ca3-R | PD-mVHs⁻ | 577 | 8 | 2 | 0 |
|  | PD-mVHs^+^ | 64 | 9 | 2 | 0 |
| stratum lacunosum moleculare of rostral ca1-L | PD-mVHs⁻ | 577 | 284 | 48 | 2 |
|  | PD-mVHs^+^ | 64 | 287 | 42 | 5 |
| stratum lacunosum moleculare of rostral ca1-R | PD-mVHs⁻ | 577 | 291 | 43 | 2 |
|  | PD-mVHs^+^ | 64 | 298 | 38 | 5 |
| stratum lacunosum moleculare of rostral ca2-L | PD-mVHs⁻ | 577 | 24 | 6 | 0 |
|  | PD-mVHs^+^ | 64 | 24 | 5 | 1 |
| stratum lacunosum moleculare of rostral ca2-R | PD-mVHs⁻ | 577 | 26 | 5 | 0 |
|  | PD-mVHs^+^ | 64 | 27 | 5 | 1 |
| stratum lacunosum moleculare of rostral ca3-L | PD-mVHs⁻ | 577 | 28 | 6 | 0 |
|  | PD-mVHs^+^ | 64 | 29 | 6 | 1 |
| stratum lacunosum moleculare of rostral ca3-R | PD-mVHs⁻ | 577 | 30 | 6 | 0 |
|  | PD-mVHs^+^ | 64 | 31 | 6 | 1 |
| stratum lacunosum moleculare of uncal ca1-L | PD-mVHs⁻ | 577 | 148 | 26 | 1 |
|  | PD-mVHs^+^ | 64 | 148 | 28 | 4 |
| stratum lacunosum moleculare of uncal ca1-R | PD-mVHs⁻ | 577 | 137 | 24 | 1 |
|  | PD-mVHs^+^ | 64 | 139 | 25 | 3 |
| stratum lacunosum moleculare of uncal ca2-L | PD-mVHs⁻ | 577 | 50 | 10 | 0 |
|  | PD-mVHs^+^ | 64 | 49 | 10 | 1 |
| stratum lacunosum moleculare of uncal ca2-R | PD-mVHs⁻ | 577 | 51 | 10 | 0 |
|  | PD-mVHs^+^ | 64 | 51 | 11 | 1 |
| stratum lacunosum moleculare of uncal ca3-L | PD-mVHs⁻ | 577 | 16 | 3 | 0 |
|  | PD-mVHs^+^ | 64 | 16 | 3 | 0 |
| stratum lacunosum moleculare of uncal ca3-R | PD-mVHs⁻ | 577 | 17 | 4 | 0 |
|  | PD-mVHs^+^ | 64 | 18 | 4 | 1 |
| stratum lucidum of caudal ca3-L | PD-mVHs⁻ | 577 | 4 | 1 | 0 |
|  | PD-mVHs^+^ | 64 | 4 | 1 | 0 |
| stratum lucidum of caudal ca3-R | PD-mVHs⁻ | 577 | 4 | 1 | 0 |
|  | PD-mVHs^+^ | 64 | 4 | 1 | 0 |
| stratum lucidum of rostral ca3-L | PD-mVHs⁻ | 577 | 12 | 2 | 0 |
|  | PD-mVHs^+^ | 64 | 12 | 2 | 0 |
| stratum lucidum of rostral ca3-R | PD-mVHs⁻ | 577 | 13 | 2 | 0 |
|  | PD-mVHs^+^ | 64 | 13 | 2 | 0 |
| stratum lucidum of uncal ca3-L | PD-mVHs⁻ | 577 | 10 | 2 | 0 |
|  | PD-mVHs^+^ | 64 | 10 | 2 | 0 |
| stratum lucidum of uncal ca3-R | PD-mVHs⁻ | 577 | 10 | 2 | 0 |
|  | PD-mVHs^+^ | 64 | 11 | 2 | 0 |
| stratum oriens of caudal ca1-L | PD-mVHs⁻ | 577 | 25 | 5 | 0 |
|  | PD-mVHs^+^ | 64 | 26 | 5 | 1 |
| stratum oriens of caudal ca1-R | PD-mVHs⁻ | 577 | 24 | 5 | 0 |
|  | PD-mVHs^+^ | 64 | 25 | 5 | 1 |
| stratum oriens of caudal ca2-L | PD-mVHs⁻ | 577 | 2 | 0 | 0 |
|  | PD-mVHs^+^ | 64 | 2 | 0 | 0 |
| stratum oriens of caudal ca2-R | PD-mVHs⁻ | 577 | 2 | 0 | 0 |
|  | PD-mVHs^+^ | 64 | 2 | 1 | 0 |
| stratum oriens of caudal ca3-L | PD-mVHs⁻ | 577 | 7 | 1 | 0 |
|  | PD-mVHs^+^ | 64 | 7 | 1 | 0 |
| stratum oriens of caudal ca3-R | PD-mVHs⁻ | 577 | 8 | 2 | 0 |
|  | PD-mVHs^+^ | 64 | 8 | 2 | 0 |
| stratum oriens of rostral ca1-L | PD-mVHs⁻ | 577 | 68 | 10 | 0 |
|  | PD-mVHs^+^ | 64 | 70 | 9 | 1 |
| stratum oriens of rostral ca1-R | PD-mVHs⁻ | 577 | 70 | 9 | 0 |
|  | PD-mVHs^+^ | 64 | 72 | 9 | 1 |
| stratum oriens of rostral ca2-L | PD-mVHs⁻ | 577 | 4 | 1 | 0 |
|  | PD-mVHs^+^ | 64 | 4 | 1 | 0 |
| stratum oriens of rostral ca2-R | PD-mVHs⁻ | 577 | 4 | 1 | 0 |
|  | PD-mVHs^+^ | 64 | 4 | 1 | 0 |
| stratum oriens of rostral ca3-L | PD-mVHs⁻ | 577 | 26 | 5 | 0 |
|  | PD-mVHs^+^ | 64 | 27 | 4 | 1 |
| stratum oriens of rostral ca3-R | PD-mVHs⁻ | 577 | 27 | 5 | 0 |
|  | PD-mVHs^+^ | 64 | 27 | 5 | 1 |
| stratum oriens of uncal ca1-L | PD-mVHs⁻ | 577 | 23 | 4 | 0 |
|  | PD-mVHs^+^ | 64 | 23 | 4 | 0 |
| stratum oriens of uncal ca1-R | PD-mVHs⁻ | 577 | 22 | 4 | 0 |
|  | PD-mVHs^+^ | 64 | 23 | 4 | 0 |
| stratum oriens of uncal ca2-L | PD-mVHs⁻ | 577 | 15 | 3 | 0 |
|  | PD-mVHs^+^ | 64 | 15 | 3 | 0 |
| stratum oriens of uncal ca2-R | PD-mVHs⁻ | 577 | 15 | 3 | 0 |
|  | PD-mVHs^+^ | 64 | 15 | 3 | 0 |
| stratum oriens of uncal ca3-L | PD-mVHs⁻ | 577 | 21 | 4 | 0 |
|  | PD-mVHs^+^ | 64 | 21 | 4 | 1 |
| stratum oriens of uncal ca3-R | PD-mVHs⁻ | 577 | 21 | 5 | 0 |
|  | PD-mVHs^+^ | 64 | 22 | 5 | 1 |
| stratum pyramidale of caudal ca1-L | PD-mVHs⁻ | 577 | 100 | 16 | 1 |
|  | PD-mVHs^+^ | 64 | 100 | 16 | 2 |
| stratum pyramidale of caudal ca1-R | PD-mVHs⁻ | 577 | 94 | 15 | 1 |
|  | PD-mVHs^+^ | 64 | 97 | 16 | 2 |
| stratum pyramidale of caudal ca2-L | PD-mVHs⁻ | 577 | 5 | 1 | 0 |
|  | PD-mVHs^+^ | 64 | 5 | 2 | 0 |
| stratum pyramidale of caudal ca2-R | PD-mVHs⁻ | 577 | 6 | 1 | 0 |
|  | PD-mVHs^+^ | 64 | 6 | 2 | 0 |
| stratum pyramidale of caudal ca3-L | PD-mVHs⁻ | 577 | 10 | 2 | 0 |
|  | PD-mVHs^+^ | 64 | 10 | 2 | 0 |
| stratum pyramidale of caudal ca3-R | PD-mVHs⁻ | 577 | 10 | 2 | 0 |
|  | PD-mVHs^+^ | 64 | 11 | 2 | 0 |
| stratum pyramidale of rostral ca1-L | PD-mVHs⁻ | 577 | 375 | 52 | 2 |
|  | PD-mVHs^+^ | 64 | 381 | 47 | 6 |
| stratum pyramidale of rostral ca1-R | PD-mVHs⁻ | 577 | 377 | 50 | 2 |
|  | PD-mVHs^+^ | 64 | 387 | 44 | 5 |
| stratum pyramidale of rostral ca2-L | PD-mVHs⁻ | 577 | 12 | 3 | 0 |
|  | PD-mVHs^+^ | 64 | 12 | 2 | 0 |
| stratum pyramidale of rostral ca2-R | PD-mVHs⁻ | 577 | 13 | 3 | 0 |
|  | PD-mVHs^+^ | 64 | 13 | 3 | 0 |
| stratum pyramidale of rostral ca3-L | PD-mVHs⁻ | 577 | 42 | 8 | 0 |
|  | PD-mVHs^+^ | 64 | 42 | 6 | 1 |
| stratum pyramidale of rostral ca3-R | PD-mVHs⁻ | 577 | 43 | 8 | 0 |
|  | PD-mVHs^+^ | 64 | 43 | 7 | 1 |
| stratum pyramidale of uncal ca1-L | PD-mVHs⁻ | 577 | 174 | 29 | 1 |
|  | PD-mVHs^+^ | 64 | 172 | 30 | 4 |
| stratum pyramidale of uncal ca1-R | PD-mVHs⁻ | 577 | 166 | 28 | 1 |
|  | PD-mVHs^+^ | 64 | 166 | 27 | 3 |
| stratum pyramidale of uncal ca2-L | PD-mVHs⁻ | 577 | 51 | 8 | 0 |
|  | PD-mVHs^+^ | 64 | 51 | 9 | 1 |
| stratum pyramidale of uncal ca2-R | PD-mVHs⁻ | 577 | 50 | 8 | 0 |
|  | PD-mVHs^+^ | 64 | 50 | 8 | 1 |
| stratum pyramidale of uncal ca3-L | PD-mVHs⁻ | 577 | 36 | 6 | 0 |
|  | PD-mVHs^+^ | 64 | 37 | 7 | 1 |
| stratum pyramidale of uncal ca3-R | PD-mVHs⁻ | 577 | 38 | 7 | 0 |
|  | PD-mVHs^+^ | 64 | 38 | 7 | 1 |
| stratum radiatum of caudal ca1-L | PD-mVHs⁻ | 577 | 27 | 5 | 0 |
|  | PD-mVHs^+^ | 64 | 28 | 5 | 1 |
| stratum radiatum of caudal ca1-R | PD-mVHs⁻ | 577 | 26 | 5 | 0 |
|  | PD-mVHs^+^ | 64 | 27 | 5 | 1 |
| stratum radiatum of caudal ca2-L | PD-mVHs⁻ | 577 | 3 | 1 | 0 |
|  | PD-mVHs^+^ | 64 | 3 | 1 | 0 |
| stratum radiatum of caudal ca2-R | PD-mVHs⁻ | 577 | 3 | 1 | 0 |
|  | PD-mVHs^+^ | 64 | 3 | 1 | 0 |
| stratum radiatum of rostral ca1-L | PD-mVHs⁻ | 577 | 107 | 17 | 1 |
|  | PD-mVHs^+^ | 64 | 108 | 16 | 2 |
| stratum radiatum of rostral ca1-R | PD-mVHs⁻ | 577 | 105 | 16 | 1 |
|  | PD-mVHs^+^ | 64 | 107 | 13 | 2 |
| stratum radiatum of rostral ca2-L | PD-mVHs⁻ | 577 | 5 | 1 | 0 |
|  | PD-mVHs^+^ | 64 | 5 | 1 | 0 |
| stratum radiatum of rostral ca2-R | PD-mVHs⁻ | 577 | 5 | 1 | 0 |
|  | PD-mVHs^+^ | 64 | 5 | 1 | 0 |
| stratum radiatum of uncal ca1-L | PD-mVHs⁻ | 577 | 40 | 7 | 0 |
|  | PD-mVHs^+^ | 64 | 40 | 6 | 1 |
| stratum radiatum of uncal ca1-R | PD-mVHs⁻ | 577 | 38 | 7 | 0 |
|  | PD-mVHs^+^ | 64 | 38 | 6 | 1 |
| stratum radiatum of uncal ca2-L | PD-mVHs⁻ | 577 | 19 | 3 | 0 |
|  | PD-mVHs^+^ | 64 | 19 | 3 | 0 |
| stratum radiatum of uncal ca2-R | PD-mVHs⁻ | 577 | 19 | 3 | 0 |
|  | PD-mVHs^+^ | 64 | 19 | 3 | 0 |
| stria medullaris of thalamus-L | PD-mVHs⁻ | 577 | 23 | 11 | 0 |
|  | PD-mVHs^+^ | 64 | 20 | 8 | 1 |
| stria medullaris of thalamus-R | PD-mVHs⁻ | 577 | 17 | 10 | 0 |
|  | PD-mVHs^+^ | 64 | 17 | 11 | 1 |
| stria terminalis-L | PD-mVHs⁻ | 577 | 153 | 20 | 1 |
|  | PD-mVHs^+^ | 64 | 155 | 21 | 3 |
| stria terminalis-R | PD-mVHs⁻ | 577 | 155 | 21 | 1 |
|  | PD-mVHs^+^ | 64 | 156 | 22 | 3 |
| subparafascicular nucleus of thalamus-L | PD-mVHs⁻ | 577 | 7 | 2 | 0 |
|  | PD-mVHs^+^ | 64 | 7 | 2 | 0 |
| subparafascicular nucleus of thalamus-R | PD-mVHs⁻ | 577 | 7 | 2 | 0 |
|  | PD-mVHs^+^ | 64 | 7 | 2 | 0 |
| substantia innominata-L | PD-mVHs⁻ | 577 | 244 | 46 | 2 |
|  | PD-mVHs^+^ | 64 | 242 | 48 | 6 |
| substantia innominata-R | PD-mVHs⁻ | 577 | 284 | 44 | 2 |
|  | PD-mVHs^+^ | 64 | 279 | 49 | 6 |
| substantia nigra compact part-L | PD-mVHs⁻ | 577 | 132 | 30 | 1 |
|  | PD-mVHs^+^ | 64 | 126 | 29 | 4 |
| substantia nigra compact part-R | PD-mVHs⁻ | 577 | 133 | 29 | 1 |
|  | PD-mVHs^+^ | 64 | 128 | 23 | 3 |
| substantia nigra reticular part-L | PD-mVHs⁻ | 577 | 144 | 35 | 1 |
|  | PD-mVHs^+^ | 64 | 139 | 31 | 4 |
| substantia nigra reticular part-R | PD-mVHs⁻ | 577 | 144 | 33 | 1 |
|  | PD-mVHs^+^ | 64 | 140 | 26 | 3 |
| superficial pulvinar nucleus-L | PD-mVHs⁻ | 577 | 9 | 2 | 0 |
|  | PD-mVHs^+^ | 64 | 10 | 1 | 0 |
| superficial pulvinar nucleus-R | PD-mVHs⁻ | 577 | 9 | 2 | 0 |
|  | PD-mVHs^+^ | 64 | 9 | 2 | 0 |
| superior cerebellar peduncle brachium conjunctivum-L | PD-mVHs⁻ | 577 | 215 | 35 | 1 |
|  | PD-mVHs^+^ | 64 | 210 | 33 | 4 |
| superior cerebellar peduncle brachium conjunctivum-R | PD-mVHs⁻ | 577 | 207 | 34 | 1 |
|  | PD-mVHs^+^ | 64 | 206 | 32 | 4 |
| suprachiasmatic nucleus-L | PD-mVHs⁻ | 577 | 0 | 0 | 0 |
|  | PD-mVHs^+^ | 64 | 0 | 0 | 0 |
| suprachiasmatic nucleus-R | PD-mVHs⁻ | 577 | 1 | 0 | 0 |
|  | PD-mVHs^+^ | 64 | 0 | 0 | 0 |
| suprageniculate nucleus of thalamus-L | PD-mVHs⁻ | 577 | 3 | 2 | 0 |
|  | PD-mVHs^+^ | 64 | 3 | 1 | 0 |
| suprageniculate nucleus of thalamus-R | PD-mVHs⁻ | 577 | 3 | 2 | 0 |
|  | PD-mVHs^+^ | 64 | 2 | 1 | 0 |
| supramammillary nucleus-L | PD-mVHs⁻ | 577 | 2 | 1 | 0 |
|  | PD-mVHs^+^ | 64 | 2 | 1 | 0 |
| supramammillary nucleus-R | PD-mVHs⁻ | 577 | 2 | 1 | 0 |
|  | PD-mVHs^+^ | 64 | 2 | 1 | 0 |
| supraoptic dicussation-L | PD-mVHs⁻ | 577 | 2 | 1 | 0 |
|  | PD-mVHs^+^ | 64 | 2 | 1 | 0 |
| supraoptic dicussation-R | PD-mVHs⁻ | 577 | 3 | 1 | 0 |
|  | PD-mVHs^+^ | 64 | 2 | 1 | 0 |
| supraoptic nucleus-L | PD-mVHs⁻ | 577 | 10 | 3 | 0 |
|  | PD-mVHs^+^ | 64 | 10 | 2 | 0 |
| supraoptic nucleus-R | PD-mVHs⁻ | 577 | 12 | 3 | 0 |
|  | PD-mVHs^+^ | 64 | 11 | 3 | 0 |
| supraoptic region of hth-L | PD-mVHs⁻ | 577 | 37 | 9 | 0 |
|  | PD-mVHs^+^ | 64 | 37 | 8 | 1 |
| supraoptic region of hth-R | PD-mVHs⁻ | 577 | 42 | 9 | 0 |
|  | PD-mVHs^+^ | 64 | 41 | 9 | 1 |
| tail of caudate-L | PD-mVHs⁻ | 577 | 97 | 17 | 1 |
|  | PD-mVHs^+^ | 64 | 97 | 13 | 2 |
| tail of caudate-R | PD-mVHs⁻ | 577 | 101 | 17 | 1 |
|  | PD-mVHs^+^ | 64 | 99 | 14 | 2 |
| temporal claustrum-L | PD-mVHs⁻ | 577 | 210 | 51 | 2 |
|  | PD-mVHs^+^ | 64 | 208 | 47 | 6 |
| temporal claustrum-R | PD-mVHs⁻ | 577 | 216 | 52 | 2 |
|  | PD-mVHs^+^ | 64 | 210 | 40 | 5 |
| thalamic fasciculus-L | PD-mVHs⁻ | 577 | 15 | 4 | 0 |
|  | PD-mVHs^+^ | 64 | 16 | 3 | 0 |
| thalamic fasciculus-R | PD-mVHs⁻ | 577 | 15 | 4 | 0 |
|  | PD-mVHs^+^ | 64 | 16 | 3 | 0 |
| thalamus-L | PD-mVHs⁻ | 577 | 442 | 69 | 3 |
|  | PD-mVHs^+^ | 64 | 429 | 56 | 7 |
| thalamus-R | PD-mVHs⁻ | 577 | 459 | 67 | 3 |
|  | PD-mVHs^+^ | 64 | 447 | 52 | 6 |
| tuberomammillary nucleus-L | PD-mVHs⁻ | 577 | 31 | 7 | 0 |
|  | PD-mVHs^+^ | 64 | 32 | 7 | 1 |
| tuberomammillary nucleus-R | PD-mVHs⁻ | 577 | 34 | 7 | 0 |
|  | PD-mVHs^+^ | 64 | 33 | 7 | 1 |
| uncal subiculum-L | PD-mVHs⁻ | 577 | 85 | 16 | 1 |
|  | PD-mVHs^+^ | 64 | 84 | 16 | 2 |
| uncal subiculum-R | PD-mVHs⁻ | 577 | 77 | 16 | 1 |
|  | PD-mVHs^+^ | 64 | 75 | 12 | 1 |
| ventral division of basomedial nucleus-L | PD-mVHs⁻ | 577 | 264 | 40 | 2 |
|  | PD-mVHs^+^ | 64 | 263 | 42 | 5 |
| ventral division of basomedial nucleus-R | PD-mVHs⁻ | 577 | 294 | 46 | 2 |
|  | PD-mVHs^+^ | 64 | 298 | 46 | 6 |
| ventral medial nucleus of thalamus-L | PD-mVHs⁻ | 577 | 30 | 8 | 0 |
|  | PD-mVHs^+^ | 64 | 32 | 10 | 1 |
| ventral medial nucleus of thalamus-R | PD-mVHs⁻ | 577 | 32 | 9 | 0 |
|  | PD-mVHs^+^ | 64 | 31 | 8 | 1 |
| ventral pallidus-L | PD-mVHs⁻ | 577 | 30 | 9 | 0 |
|  | PD-mVHs^+^ | 64 | 31 | 9 | 1 |
| ventral pallidus-R | PD-mVHs⁻ | 577 | 29 | 8 | 0 |
|  | PD-mVHs^+^ | 64 | 29 | 8 | 1 |
| ventral part of ventromedial hypothalamic nucleus-L | PD-mVHs⁻ | 577 | 9 | 2 | 0 |
|  | PD-mVHs^+^ | 64 | 9 | 2 | 0 |
| ventral part of ventromedial hypothalamic nucleus-R | PD-mVHs⁻ | 577 | 10 | 2 | 0 |
|  | PD-mVHs^+^ | 64 | 10 | 2 | 0 |
| ventral posterior inferior nucleus-L | PD-mVHs⁻ | 577 | 59 | 14 | 1 |
|  | PD-mVHs^+^ | 64 | 58 | 11 | 1 |
| ventral posterior inferior nucleus-R | PD-mVHs⁻ | 577 | 58 | 15 | 1 |
|  | PD-mVHs^+^ | 64 | 57 | 10 | 1 |
| ventral posterior medial nucleus-L | PD-mVHs⁻ | 577 | 28 | 9 | 0 |
|  | PD-mVHs^+^ | 64 | 27 | 8 | 1 |
| ventral posterior medial nucleus-R | PD-mVHs⁻ | 577 | 28 | 9 | 0 |
|  | PD-mVHs^+^ | 64 | 27 | 9 | 1 |
| ventral subdivision of coa-L | PD-mVHs⁻ | 577 | 19 | 3 | 0 |
|  | PD-mVHs^+^ | 64 | 19 | 3 | 0 |
| ventral subdivision of coa-R | PD-mVHs⁻ | 577 | 23 | 3 | 0 |
|  | PD-mVHs^+^ | 64 | 24 | 4 | 0 |
| ventral subdivision of vlc-L | PD-mVHs⁻ | 577 | 195 | 63 | 3 |
|  | PD-mVHs^+^ | 64 | 190 | 63 | 8 |
| ventral subdivision of vlc-R | PD-mVHs⁻ | 577 | 209 | 63 | 3 |
|  | PD-mVHs^+^ | 64 | 206 | 60 | 8 |
| ventral tegmental area-L | PD-mVHs⁻ | 577 | 178 | 38 | 2 |
|  | PD-mVHs^+^ | 64 | 173 | 41 | 5 |
| ventral tegmental area-R | PD-mVHs⁻ | 577 | 164 | 36 | 1 |
|  | PD-mVHs^+^ | 64 | 159 | 30 | 4 |
| white matter of forebrain-L | PD-mVHs⁻ | 577 | 237024 | 26420 | 1100 |
|  | PD-mVHs^+^ | 64 | 234949 | 23272 | 2909 |
| white matter of forebrain-R | PD-mVHs⁻ | 577 | 234140 | 26025 | 1083 |
|  | PD-mVHs^+^ | 64 | 231177 | 23503 | 2938 |
| white matter of hindbrain-L | PD-mVHs⁻ | 577 | 3448 | 440 | 18 |
|  | PD-mVHs^+^ | 64 | 3416 | 436 | 55 |
| white matter of hindbrain-R | PD-mVHs⁻ | 577 | 3425 | 439 | 18 |
|  | PD-mVHs^+^ | 64 | 3383 | 436 | 54 |
| white matter of the cerebellum-L | PD-mVHs⁻ | 577 | 15735 | 2015 | 84 |
|  | PD-mVHs^+^ | 64 | 15317 | 1786 | 223 |
| white matter of the cerebellum-R | PD-mVHs⁻ | 577 | 14822 | 1916 | 80 |
|  | PD-mVHs^+^ | 64 | 14380 | 1687 | 211 |
| zona incerta-L | PD-mVHs⁻ | 577 | 86 | 30 | 1 |
|  | PD-mVHs^+^ | 64 | 86 | 16 | 2 |
| zona incerta-R | PD-mVHs⁻ | 577 | 89 | 29 | 1 |
|  | PD-mVHs^+^ | 64 | 86 | 13 | 2 |

*Abbreviations:* COA=Cortical amygdaloid area; eTIV=Estimated Total Intracranial Volume; L=left; MD=Mediodorsal nucleus of the thalamus; PVA=Paraventricular Anterior nucleus; R=right; VPsth=Subthalamic nucleus; VA=Ventroanterior nucleus of thalamus; VL=Ventrolateral nucleus of thalamus; VLC=Ventral Lateral nucleus pars caudalis of the thalamus; VPM=Ventral Posteromedial nucleus of the thalamus.

**Table S5. ANCOVA outputs for the exploratory volumetric analysis of subcortical structures segmented using the HistoAtlas integrated within NextBrain.** ANCOVA models included group and sex as fixed factors, together with the group × sex interaction, and age, education, standardized eTIV, MoCA score, and disease duration as continuous covariates.

| Structure | F | p-value | Observed power (alpha=0.05) | Partial eta squared |
| --- | --- | --- | --- | --- |
| alveus-L | 0.153 | 0.696 | 0.068 | <0.001 |
| alveus-R | 0.143 | 0.705 | 0.067 | <0.001 |
| amygdalocortical corticoamygdaloid transition area-L | 1.754 | 0.186 | 0.262 | 0.003 |
| amygdalocortical corticoamygdaloid transition area-R | 0.280 | 0.597 | 0.083 | <0.001 |
| amygdalohippocampal area-L | <0.001 | 0.985 | 0.050 | <0.001 |
| amygdalohippocampal area-R | 0.440 | 0.507 | 0.102 | 0.001 |
| amygdalohippocampal transition area-L | 0.246 | 0.620 | 0.079 | <0.001 |
| amygdalohippocampal transition area-R | 0.502 | 0.479 | 0.109 | 0.001 |
| amygdaloid complex-L | 0.170 | 0.680 | 0.070 | <0.001 |
| amygdaloid complex-R | 0.114 | 0.736 | 0.063 | <0.001 |
| amygdalostriatal transition area-L | 0.625 | 0.430 | 0.124 | 0.001 |
| amygdalostriatal transition area-R | 0.751 | 0.386 | 0.139 | 0.001 |
| anterior amygdaloid area-L | 0.145 | 0.704 | 0.067 | <0.001 |
| anterior amygdaloid area-R | 0.503 | 0.478 | 0.109 | 0.001 |
| anterior commissure-L | 0.017 | 0.898 | 0.052 | <0.001 |
| anterior commissure-R | 0.246 | 0.620 | 0.079 | <0.001 |
| anterior hypothalamic nucleus-L | 0.113 | 0.737 | 0.063 | <0.001 |
| anterior hypothalamic nucleus-R | 1.812 | 0.179 | 0.269 | 0.003 |
| anterior nucleus of pulvinar-L | 1.069 | 0.301 | 0.178 | 0.002 |
| anterior nucleus of pulvinar-R | 0.424 | 0.515 | 0.100 | 0.001 |
| anterior olfactory nucleus-L | 2.588 | 0.108 | 0.362 | 0.004 |
| anterior olfactory nucleus-R | 1.127 | 0.289 | 0.185 | 0.002 |
| anterodorsal nucleus of thalamus-L | 0.981 | 0.322 | 0.167 | 0.002 |
| anterodorsal nucleus of thalamus-R | 0.209 | 0.648 | 0.074 | <0.001 |
| anteromedial nucleus of thalamus-L | 1.467 | 0.226 | 0.227 | 0.002 |
| anteromedial nucleus of thalamus-R | 0.025 | 0.875 | 0.053 | <0.001 |
| anteroventral nucleus of thalamus-L | 2.070 | 0.151 | 0.301 | 0.003 |
| anteroventral nucleus of thalamus-R | 0.252 | 0.616 | 0.079 | <0.001 |
| arcuate nucleus of hypothalamus-L | 0.673 | 0.412 | 0.130 | 0.001 |
| arcuate nucleus of hypothalamus-R | 0.185 | 0.667 | 0.071 | <0.001 |
| basal division of medial mammillary nucleus-L | 1.790 | 0.181 | 0.267 | 0.003 |
| basal division of medial mammillary nucleus-R | 2.178 | 0.141 | 0.314 | 0.003 |
| basal nucleus of meynert-L | 0.143 | 0.706 | 0.066 | <0.001 |
| basal nucleus of meynert-R | 0.110 | 0.741 | 0.063 | <0.001 |
| basal ventral medial nucleus-L | 1.328 | 0.250 | 0.210 | 0.002 |
| basal ventral medial nucleus-R | 1.366 | 0.243 | 0.215 | 0.002 |
| basolateral nucleus basal nucleus-L | 0.287 | 0.592 | 0.083 | <0.001 |
| basolateral nucleus basal nucleus-R | 0.015 | 0.903 | 0.052 | <0.001 |
| bed nucleus of stria terminalis-L | 0.214 | 0.644 | 0.075 | <0.001 |
| bed nucleus of stria terminalis-R | 0.124 | 0.725 | 0.064 | <0.001 |
| body of caudate-L | 0.012 | 0.913 | 0.051 | <0.001 |
| body of caudate-R | 0.151 | 0.698 | 0.067 | <0.001 |
| caudal division of ventral posterior lateral nucleus-L | 2.642 | 0.105 | 0.368 | 0.004 |
| caudal division of ventral posterior lateral nucleus-R | 2.241 | 0.135 | 0.321 | 0.004 |
| caudal subiculum-L | 0.605 | 0.437 | 0.121 | 0.001 |
| caudal subiculum-R | 0.638 | 0.425 | 0.125 | 0.001 |
| central dorsal nucleus of thalamus-L | 0.490 | 0.484 | 0.108 | 0.001 |
| central dorsal nucleus of thalamus-R | 0.029 | 0.866 | 0.053 | <0.001 |
| central gray of medulla oblongata-L | 0.164 | 0.685 | 0.069 | <0.001 |
| central gray of medulla oblongata-R | 2.329 | 0.128 | 0.332 | 0.004 |
| central medial nucleus of thalamus-L | 0.005 | 0.941 | 0.051 | <0.001 |
| central medial nucleus of thalamus-R | 0.242 | 0.623 | 0.078 | <0.001 |
| central nucleus of inferior colliculus-L | 0.088 | 0.767 | 0.060 | <0.001 |
| central nucleus of inferior colliculus-R | 0.212 | 0.645 | 0.075 | <0.001 |
| central part of medial division of md-L | 0.028 | 0.867 | 0.053 | <0.001 |
| central part of medial division of md-R | 1.195 | 0.275 | 0.194 | 0.002 |
| central part of ventromedial hypothalamic nucleus-L | 0.072 | 0.789 | 0.058 | <0.001 |
| central part of ventromedial hypothalamic nucleus-R | 2.398 | 0.122 | 0.340 | 0.004 |
| centromedian nucleus of thalamus-L | 0.385 | 0.535 | 0.095 | 0.001 |
| centromedian nucleus of thalamus-R | 1.210 | 0.272 | 0.196 | 0.002 |
| claustrum-L | 0.210 | 0.647 | 0.074 | <0.001 |
| claustrum-R | 0.038 | 0.846 | 0.054 | <0.001 |
| core of nucleus accumbens-L | 1.190 | 0.276 | 0.193 | 0.002 |
| core of nucleus accumbens-R | 1.550 | 0.214 | 0.237 | 0.002 |
| densocellular paralamellar division of md-L | 0.049 | 0.826 | 0.056 | <0.001 |
| densocellular paralamellar division of md-R | 1.254 | 0.263 | 0.201 | 0.002 |
| dentate lateral nucleus-L | 12.690 | <0.001 | 0.945 | 0.020 |
| dentate lateral nucleus-R | 1.542 | 0.215 | 0.236 | 0.002 |
| diagonal band-L | 0.869 | 0.351 | 0.154 | 0.001 |
| diagonal band-R | 2.000 | 0.158 | 0.292 | 0.003 |
| diffuse pulvinar nucleus-L | 0.656 | 0.418 | 0.128 | 0.001 |
| diffuse pulvinar nucleus-R | <0.001 | 0.985 | 0.050 | <0.001 |
| dorsal division of central lateral nucleus-L | 0.074 | 0.786 | 0.058 | <0.001 |
| dorsal division of central lateral nucleus-R | 0.015 | 0.904 | 0.052 | <0.001 |
| dorsal lateral geniculate nucleus-L | 0.438 | 0.508 | 0.101 | 0.001 |
| dorsal lateral geniculate nucleus-R | 0.180 | 0.672 | 0.071 | <0.001 |
| dorsal medial geniculate nucleus-L | 0.003 | 0.958 | 0.050 | <0.001 |
| dorsal medial geniculate nucleus-R | 1.394 | 0.238 | 0.218 | 0.002 |
| dorsal part of ventromedial hypothalamic nucleus-L | 0.003 | 0.956 | 0.050 | <0.001 |
| dorsal part of ventromedial hypothalamic nucleus-R | 0.924 | 0.337 | 0.160 | 0.001 |
| dorsal raphe nucleus-L | 1.357 | 0.245 | 0.214 | 0.002 |
| dorsal raphe nucleus-R | 0.708 | 0.400 | 0.134 | 0.001 |
| dorsal subdivision of vlc-L | 2.098 | 0.148 | 0.304 | 0.003 |
| dorsal subdivision of vlc-R | 1.988 | 0.159 | 0.291 | 0.003 |
| dorsomedial hypothalamic nucleus-L | 0.654 | 0.419 | 0.127 | 0.001 |
| dorsomedial hypothalamic nucleus-R | 0.220 | 0.640 | 0.075 | <0.001 |
| external segment of globus pallidus-L | 0.504 | 0.478 | 0.109 | 0.001 |
| external segment of globus pallidus-R | 1.112 | 0.292 | 0.183 | 0.002 |
| facial nucleus-L | 0.013 | 0.908 | 0.052 | <0.001 |
| facial nucleus-R | 0.532 | 0.466 | 0.113 | 0.001 |
| fasciculosus nucleus of thalamus-L | 0.924 | 0.337 | 0.160 | 0.001 |
| fasciculosus nucleus of thalamus-R | 0.340 | 0.560 | 0.090 | 0.001 |
| fasciculus retroflexus habenuno interpeduncular tract-L | 0.709 | 0.400 | 0.134 | 0.001 |
| fasciculus retroflexus habenuno interpeduncular tract-R | 1.132 | 0.288 | 0.186 | 0.002 |
| fastigial medial nucleus-L | 0.114 | 0.736 | 0.063 | <0.001 |
| fastigial medial nucleus-R | 3.411 | 0.065 | 0.454 | 0.005 |
| fimbria-L | 1.550 | 0.214 | 0.237 | 0.002 |
| fimbria-R | 1.686 | 0.195 | 0.254 | 0.003 |
| fornix-L | 0.341 | 0.559 | 0.090 | 0.001 |
| fornix-R | 0.180 | 0.672 | 0.071 | <0.001 |
| granular cell layer of pva-L | 3.755 | 0.053 | 0.490 | 0.006 |
| granular cell layer of pva-R | 3.068 | 0.080 | 0.417 | 0.005 |
| granular layer of caudal dentate gyrus-L | 0.420 | 0.517 | 0.099 | 0.001 |
| granular layer of caudal dentate gyrus-R | 2.552 | 0.111 | 0.358 | 0.004 |
| granular layer of rostral dentate gyrus-L | 2.107 | 0.147 | 0.305 | 0.003 |
| granular layer of rostral dentate gyrus-R | 1.144 | 0.285 | 0.187 | 0.002 |
| granular layer of uncal dentate gyrus-L | 3.374 | 0.067 | 0.450 | 0.005 |
| granular layer of uncal dentate gyrus-R | 0.407 | 0.524 | 0.098 | 0.001 |
| habenular commissure-L | 4.464 | 0.035 | 0.559 | 0.007 |
| habenular commissure-R | 1.969 | 0.161 | 0.289 | 0.003 |
| head of caudate-L | 0.276 | 0.600 | 0.082 | <0.001 |
| head of caudate-R | 0.002 | 0.960 | 0.050 | <0.001 |
| inferior cerebellar peduncle-L | 0.568 | 0.452 | 0.117 | 0.001 |
| inferior cerebellar peduncle-R | 2.267 | 0.133 | 0.324 | 0.004 |
| inferior nucleus of pulvinar-L | 0.250 | 0.617 | 0.079 | <0.001 |
| inferior nucleus of pulvinar-R | 0.939 | 0.333 | 0.162 | 0.001 |
| inferior olive principal nucleus-L | 0.031 | 0.859 | 0.054 | <0.001 |
| inferior olive principal nucleus-R | 0.127 | 0.721 | 0.065 | <0.001 |
| inferior thalamic peduncle-L | 1.049 | 0.306 | 0.176 | 0.002 |
| inferior thalamic peduncle-R | 0.514 | 0.473 | 0.111 | 0.001 |
| infundibular stalk-L | 0.072 | 0.788 | 0.058 | <0.001 |
| infundibular stalk-R | 0.040 | 0.842 | 0.055 | <0.001 |
| intermediodorsal nucleus of thalamus-L | 0.004 | 0.952 | 0.050 | <0.001 |
| intermediodorsal nucleus of thalamus-R | 0.536 | 0.464 | 0.113 | 0.001 |
| internal segment of globus pallidus-L | 1.257 | 0.263 | 0.201 | 0.002 |
| internal segment of globus pallidus-R | 0.067 | 0.796 | 0.058 | <0.001 |
| interstitial nucleus of cajal-L | 0.083 | 0.774 | 0.059 | <0.001 |
| interstitial nucleus of cajal-R | 0.007 | 0.932 | 0.051 | <0.001 |
| juxtaparaventricular lateral hypothalamic area-L | 0.431 | 0.512 | 0.101 | 0.001 |
| juxtaparaventricular lateral hypothalamic area-R | 0.681 | 0.410 | 0.131 | 0.001 |
| lateral hypothalamic area posterior part-L | 0.151 | 0.698 | 0.067 | <0.001 |
| lateral hypothalamic area posterior part-R | 6.277 | 0.012 | 0.706 | 0.010 |
| lateral division of central lateral nucleus-L | 2.814 | 0.094 | 0.388 | 0.004 |
| lateral division of central lateral nucleus-R | 3.361 | 0.067 | 0.449 | 0.005 |
| lateral division of centromedian nucleus of thalamus-L | 1.303 | 0.254 | 0.207 | 0.002 |
| lateral division of centromedian nucleus of thalamus-R | 0.813 | 0.367 | 0.147 | 0.001 |
| lateral dorsal nucleus of thalamus-L | 0.101 | 0.751 | 0.062 | <0.001 |
| lateral dorsal nucleus of thalamus-R | 0.095 | 0.758 | 0.061 | <0.001 |
| lateral habenular nucleus-L | 0.491 | 0.484 | 0.108 | 0.001 |
| lateral habenular nucleus-R | 0.018 | 0.892 | 0.052 | <0.001 |
| lateral hypothalamic area tuberal part-L | 2.204 | 0.138 | 0.317 | 0.003 |
| lateral hypothalamic area tuberal part-R | 0.466 | 0.495 | 0.105 | 0.001 |
| lateral interpositus emboliform nucleus-L | 2.133 | 0.145 | 0.308 | 0.003 |
| lateral interpositus emboliform nucleus-R | 3.162 | 0.076 | 0.427 | 0.005 |
| lateral mammillary nucleus-L | 0.018 | 0.892 | 0.052 | <0.001 |
| lateral mammillary nucleus-R | 0.006 | 0.940 | 0.051 | <0.001 |
| lateral nucleus-L | 0.041 | 0.840 | 0.055 | <0.001 |
| lateral nucleus of pulvinar-L | 6.010 | 0.014 | 0.687 | 0.009 |
| lateral nucleus of pulvinar-R | 1.406 | 0.236 | 0.220 | 0.002 |
| lateral nucleus-R | 0.020 | 0.886 | 0.052 | <0.001 |
| lateral olfactory area-L | 0.003 | 0.956 | 0.050 | <0.001 |
| lateral olfactory area-R | 1.178 | 0.278 | 0.192 | 0.002 |
| lateral olfactory stria-L | 2.561 | 0.110 | 0.359 | 0.004 |
| lateral olfactory stria-R | 0.344 | 0.558 | 0.090 | 0.001 |
| lateral part of medial mammillary nucleus-L | 1.260 | 0.262 | 0.202 | 0.002 |
| lateral part of medial mammillary nucleus-R | 0.400 | 0.527 | 0.097 | 0.001 |
| lateral posterior nucleus of thalamus-L | 0.355 | 0.551 | 0.091 | 0.001 |
| lateral posterior nucleus of thalamus-R | 0.647 | 0.421 | 0.127 | 0.001 |
| lateral preoptic area-L | 0.767 | 0.382 | 0.141 | 0.001 |
| lateral preoptic area-R | 1.263 | 0.262 | 0.202 | 0.002 |
| lateral subdivision of central nucleus-L | 0.674 | 0.412 | 0.130 | 0.001 |
| lateral subdivision of central nucleus-R | 0.405 | 0.525 | 0.097 | 0.001 |
| lateral tuberal nuclei-L | 2.945 | 0.087 | 0.403 | 0.005 |
| lateral tuberal nuclei-R | 0.624 | 0.430 | 0.124 | 0.001 |
| laterodorsal portion of sth-L | 0.219 | 0.640 | 0.075 | <0.001 |
| laterodorsal portion of sth-R | 0.001 | 0.970 | 0.050 | <0.001 |
| lateroventral portion of sth-L | 0.632 | 0.427 | 0.125 | 0.001 |
| lateroventral portion of sth-R | 0.483 | 0.487 | 0.107 | 0.001 |
| layer III of piriform cortex-L | 1.243 | 0.265 | 0.200 | 0.002 |
| layer III of piriform cortex-R | 0.364 | 0.547 | 0.092 | 0.001 |
| lenticular fasciculus-L | 1.473 | 0.225 | 0.228 | 0.002 |
| lenticular fasciculus-R | 0.981 | 0.322 | 0.167 | 0.002 |
| limitans nucleus-L | 0.043 | 0.835 | 0.055 | <0.001 |
| limitans nucleus-R | 0.004 | 0.948 | 0.050 | <0.001 |
| magnocellular medial division of md-L | 1.353 | 0.245 | 0.213 | 0.002 |
| magnocellular medial division of md-R | 3.677 | 0.056 | 0.482 | 0.006 |
| magnocellular medial nucleus-L | 0.042 | 0.838 | 0.055 | <0.001 |
| magnocellular medial nucleus-R | 0.348 | 0.555 | 0.091 | 0.001 |
| magnocellular division of reticular nucleus-L | 2.116 | 0.146 | 0.306 | 0.003 |
| magnocellular division of reticular nucleus-R | 0.893 | 0.345 | 0.157 | 0.001 |
| magnocellular division of va-L | 0.017 | 0.896 | 0.052 | <0.001 |
| magnocellular division of va-R | 0.079 | 0.778 | 0.059 | <0.001 |
| mammillary peduncle-L | 2.233 | 0.136 | 0.320 | 0.004 |
| mammillary peduncle-R | 0.669 | 0.414 | 0.129 | 0.001 |
| mammillothalamic tract-L | 0.079 | 0.778 | 0.059 | <0.001 |
| mammillothalamic tract-R | 0.097 | 0.756 | 0.061 | <0.001 |
| medial corticohypothalamic tract-L | 0.012 | 0.912 | 0.051 | <0.001 |
| medial corticohypothalamic tract-R | 0.131 | 0.717 | 0.065 | <0.001 |
| medial division of centromedian nucleus of thalamus-L | 0.815 | 0.367 | 0.147 | 0.001 |
| medial division of centromedian nucleus of thalamus-R | 0.192 | 0.662 | 0.072 | <0.001 |
| medial habenular nucleus-L | 0.054 | 0.816 | 0.056 | <0.001 |
| medial habenular nucleus-R | 1.470 | 0.226 | 0.228 | 0.002 |
| medial interpositus globose nucleus-L | 2.990 | 0.084 | 0.408 | 0.005 |
| medial interpositus globose nucleus-R | 6.922 | 0.009 | 0.748 | 0.011 |
| medial nucleus of pulvinar-L | 0.149 | 0.700 | 0.067 | <0.001 |
| medial nucleus of pulvinar-R | 0.007 | 0.934 | 0.051 | <0.001 |
| medial part of medial mammillary nucleus-L | 0.917 | 0.339 | 0.159 | 0.001 |
| medial part of medial mammillary nucleus-R | 1.413 | 0.235 | 0.221 | 0.002 |
| medial portion of sth-L | 0.250 | 0.618 | 0.079 | <0.001 |
| medial portion of sth-R | <0.001 | 0.992 | 0.050 | <0.001 |
| medial preoptic nucleus-L | 0.105 | 0.746 | 0.062 | <0.001 |
| medial preoptic nucleus-R | 0.836 | 0.361 | 0.150 | 0.001 |
| medial septal nucleus-L | 0.441 | 0.507 | 0.102 | 0.001 |
| medial septal nucleus-R | 2.184 | 0.140 | 0.314 | 0.003 |
| medial subdivision of central nucleus-L | 1.368 | 0.243 | 0.215 | 0.002 |
| medial subdivision of central nucleus-R | 0.306 | 0.580 | 0.086 | <0.001 |
| medial subdivision of vlc area x-L | 1.120 | 0.290 | 0.184 | 0.002 |
| medial subdivision of vlc area x-R | 1.880 | 0.171 | 0.278 | 0.003 |
| median preoptic nucleus-L | 3.562 | 0.060 | 0.470 | 0.006 |
| median preoptic nucleus-R | 1.151 | 0.284 | 0.188 | 0.002 |
| midbrain mesencephalon-L | 2.377 | 0.124 | 0.337 | 0.004 |
| midbrain mesencephalon-R | 1.593 | 0.207 | 0.243 | 0.003 |
| molecular layer of caudal dentate gyrus-L | 0.084 | 0.772 | 0.060 | <0.001 |
| molecular layer of caudal dentate gyrus-R | 1.949 | 0.163 | 0.286 | 0.003 |
| molecular layer of pva-L | 3.781 | 0.052 | 0.493 | 0.006 |
| molecular layer of pva-R | 3.097 | 0.079 | 0.420 | 0.005 |
| molecular layer of rostral dentate gyrus-L | 1.971 | 0.161 | 0.289 | 0.003 |
| molecular layer of rostral dentate gyrus-R | 0.955 | 0.329 | 0.164 | 0.002 |
| molecular layer of uncal dentate gyrus-L | 1.356 | 0.245 | 0.214 | 0.002 |
| molecular layer of uncal dentate gyrus-R | 0.292 | 0.589 | 0.084 | <0.001 |
| multiform lateral division of md-L | 0.084 | 0.772 | 0.060 | <0.001 |
| multiform lateral division of md-R | 0.260 | 0.611 | 0.080 | <0.001 |
| myelencephalon medulla oblongata-L | 1.036 | 0.309 | 0.174 | 0.002 |
| myelencephalon medulla oblongata-R | 0.026 | 0.873 | 0.053 | <0.001 |
| nucleus of the field of forel-L | 0.202 | 0.653 | 0.073 | <0.001 |
| nucleus of the field of forel-R | 0.168 | 0.682 | 0.069 | <0.001 |
| olfactory tubercle-L | 0.234 | 0.629 | 0.077 | <0.001 |
| olfactory tubercle-R | 0.137 | 0.711 | 0.066 | <0.001 |
| optic chiasm-L | 0.154 | 0.695 | 0.068 | <0.001 |
| optic chiasm-R | 0.169 | 0.681 | 0.070 | <0.001 |
| optic nerve-L | 0.680 | 0.410 | 0.130 | 0.001 |
| optic nerve-R | 0.585 | 0.445 | 0.119 | 0.001 |
| optic tract-L | 0.004 | 0.947 | 0.051 | <0.001 |
| optic tract-R | 0.126 | 0.722 | 0.065 | <0.001 |
| pallidohypothalamic area-L | 0.279 | 0.598 | 0.082 | <0.001 |
| pallidohypothalamic area-R | 0.012 | 0.913 | 0.051 | <0.001 |
| paracentral nucleus of thalamus-L | 0.808 | 0.369 | 0.146 | 0.001 |
| paracentral nucleus of thalamus-R | 0.783 | 0.377 | 0.143 | 0.001 |
| parafascicular nucleus of thalamus-L | 0.020 | 0.889 | 0.052 | <0.001 |
| parafascicular nucleus of thalamus-R | 0.060 | 0.806 | 0.057 | <0.001 |
| paralaminar nucleus-L | 0.001 | 0.974 | 0.050 | <0.001 |
| paralaminar nucleus-R | 0.073 | 0.787 | 0.058 | <0.001 |
| parasubthalamic nucleus-L | 0.986 | 0.321 | 0.168 | 0.002 |
| parasubthalamic nucleus-R | 0.312 | 0.576 | 0.086 | <0.001 |
| parataenial nucleus of thalamus-L | 3.311 | 0.069 | 0.443 | 0.005 |
| parataenial nucleus of thalamus-R | 0.004 | 0.949 | 0.050 | <0.001 |
| paraventricular nucleus-L | 3.347 | 0.068 | 0.447 | 0.005 |
| paraventricular nucleus of hypothalamus-L | 0.913 | 0.340 | 0.159 | 0.001 |
| paraventricular nucleus of hypothalamus-R | 1.050 | 0.306 | 0.176 | 0.002 |
| paraventricular nucleus-R | 0.835 | 0.361 | 0.149 | 0.001 |
| parvocellular central division of md-L | 0.225 | 0.636 | 0.076 | <0.001 |
| parvocellular central division of md-R | 1.620 | 0.204 | 0.246 | 0.003 |
| parvocellular division of retic nucl periret nucleus-L | 0.876 | 0.350 | 0.155 | 0.001 |
| parvocellular division of retic nucl periret nucleus-R | 0.506 | 0.477 | 0.110 | 0.001 |
| parvocellular division of va-L | 1.357 | 0.244 | 0.214 | 0.002 |
| parvocellular division of va-R | 0.541 | 0.462 | 0.114 | 0.001 |
| parvocellular division of vpm-L | <0.001 | 0.990 | 0.050 | <0.001 |
| parvocellular division of vpm-R | 0.434 | 0.510 | 0.101 | 0.001 |
| periaqueductal gray substance-L | 0.531 | 0.466 | 0.113 | 0.001 |
| periaqueductal gray substance-R | 0.020 | 0.886 | 0.052 | <0.001 |
| peripeduncular nucleus-L | 0.733 | 0.392 | 0.137 | 0.001 |
| peripeduncular nucleus-R | 1.546 | 0.214 | 0.237 | 0.002 |
| periventricular area of thalamus-L | 0.129 | 0.719 | 0.065 | <0.001 |
| periventricular area of thalamus-R | 0.250 | 0.617 | 0.079 | <0.001 |
| periventricular nucleus supraoptic portion-L | 0.817 | 0.366 | 0.147 | 0.001 |
| periventricular nucleus supraoptic portion-R | 0.380 | 0.538 | 0.094 | 0.001 |
| periventricular nucleus tuberal portion-L | 0.372 | 0.542 | 0.093 | 0.001 |
| periventricular nucleus tuberal portion-R | 0.039 | 0.844 | 0.054 | <0.001 |
| pineal body-L | 1.139 | 0.286 | 0.187 | 0.002 |
| pineal body-R | 0.386 | 0.535 | 0.095 | 0.001 |
| polyform layer of caudal dentate gyrus-L | 0.197 | 0.657 | 0.073 | <0.001 |
| polyform layer of caudal dentate gyrus-R | 1.875 | 0.171 | 0.277 | 0.003 |
| polyform layer of rostral dentate gyrus-L | 1.959 | 0.162 | 0.287 | 0.003 |
| polyform layer of rostral dentate gyrus-R | 0.405 | 0.525 | 0.097 | 0.001 |
| polyform layer of uncal dentate gyrus-L | 1.688 | 0.194 | 0.254 | 0.003 |
| polyform layer of uncal dentate gyrus-R | 0.089 | 0.766 | 0.060 | <0.001 |
| pons-L | 1.250 | 0.264 | 0.200 | 0.002 |
| pons-R | 0.150 | 0.699 | 0.067 | <0.001 |
| pontine nucleus-L | 2.833 | 0.093 | 0.390 | 0.004 |
| pontine nucleus-R | 0.519 | 0.472 | 0.111 | 0.001 |
| posterior hypothalamic nucleus-L | 0.974 | 0.324 | 0.167 | 0.002 |
| posterior hypothalamic nucleus-R | 4.888 | 0.027 | 0.598 | 0.008 |
| posterior nucleus of thalamus-L | 0.395 | 0.530 | 0.096 | 0.001 |
| posterior nucleus of thalamus-R | 0.645 | 0.422 | 0.126 | 0.001 |
| posteroventral putamen-L | 1.019 | 0.313 | 0.172 | 0.002 |
| posteroventral putamen-R | 0.343 | 0.558 | 0.090 | 0.001 |
| preoptic region of hth-L | 3.086 | 0.079 | 0.419 | 0.005 |
| preoptic region of hth-R | 0.546 | 0.460 | 0.114 | 0.001 |
| pretectal region-L | 1.163 | 0.281 | 0.190 | 0.002 |
| pretectal region-R | 0.082 | 0.775 | 0.059 | <0.001 |
| pulvinar of thalamus-L | 0.135 | 0.713 | 0.066 | <0.001 |
| pulvinar of thalamus-R | 0.011 | 0.918 | 0.051 | <0.001 |
| putamen-L | 0.098 | 0.754 | 0.061 | <0.001 |
| putamen-R | 0.006 | 0.940 | 0.051 | <0.001 |
| pyramidal cells of caudal ca4-L | 0.171 | 0.679 | 0.070 | <0.001 |
| pyramidal cells of caudal ca4-R | 1.840 | 0.175 | 0.273 | 0.003 |
| pyramidal cells of rostral ca4-L | 2.030 | 0.155 | 0.296 | 0.003 |
| pyramidal cells of rostral ca4-R | 1.336 | 0.248 | 0.211 | 0.002 |
| pyramidal cells of uncal ca4-L | 0.226 | 0.634 | 0.076 | <0.001 |
| pyramidal cells of uncal ca4-R | 0.004 | 0.947 | 0.050 | <0.001 |
| red nucleus-L | 0.029 | 0.865 | 0.053 | <0.001 |
| red nucleus-R | 0.004 | 0.948 | 0.050 | <0.001 |
| reticular nucleus of thalamus-L | 1.490 | 0.223 | 0.230 | 0.002 |
| reticular nucleus of thalamus-R | 2.404 | 0.122 | 0.340 | 0.004 |
| reuniens nucleus medioventral nucleus of thalamus-L | 0.163 | 0.687 | 0.069 | <0.001 |
| reuniens nucleus medioventral nucleus of thalamus-R | 1.454 | 0.228 | 0.226 | 0.002 |
| rhomboid central nucleus of thalamus-L | 0.646 | 0.422 | 0.126 | 0.001 |
| rhomboid central nucleus of thalamus-R | 1.029 | 0.311 | 0.173 | 0.002 |
| rostral division of ventral posterior lateral nucleus-L | 2.003 | 0.158 | 0.293 | 0.003 |
| rostral division of ventral posterior lateral nucleus-R | 2.051 | 0.153 | 0.298 | 0.003 |
| rostral division of vl-L | 1.254 | 0.263 | 0.201 | 0.002 |
| rostral division of vl-R | 0.964 | 0.326 | 0.165 | 0.002 |
| rostral subdivision of medial nucleus-L | 0.634 | 0.426 | 0.125 | 0.001 |
| rostral subdivision of medial nucleus-R | 2.632 | 0.105 | 0.367 | 0.004 |
| rostral subiculum-L | 0.062 | 0.804 | 0.057 | <0.001 |
| rostral subiculum-R | 0.520 | 0.471 | 0.111 | 0.001 |
| septum pellucidum-L | 0.729 | 0.393 | 0.137 | 0.001 |
| septum pellucidum-R | 0.050 | 0.823 | 0.056 | <0.001 |
| solitary tract-L | 1.566 | 0.211 | 0.239 | 0.002 |
| solitary tract-R | 2.066 | 0.151 | 0.300 | 0.003 |
| stratum lacunosum moleculare of caudal ca1-L | 0.001 | 0.980 | 0.050 | <0.001 |
| stratum lacunosum moleculare of caudal ca1-R | 1.720 | 0.190 | 0.258 | 0.003 |
| stratum lacunosum moleculare of caudal ca2-L | 0.402 | 0.526 | 0.097 | 0.001 |
| stratum lacunosum moleculare of caudal ca2-R | 2.371 | 0.124 | 0.337 | 0.004 |
| stratum lacunosum moleculare of caudal ca3-L | 0.002 | 0.961 | 0.050 | <0.001 |
| stratum lacunosum moleculare of caudal ca3-R | 2.657 | 0.104 | 0.370 | 0.004 |
| stratum lacunosum moleculare of rostral ca1-L | 0.134 | 0.714 | 0.065 | <0.001 |
| stratum lacunosum moleculare of rostral ca1-R | 0.490 | 0.484 | 0.108 | 0.001 |
| stratum lacunosum moleculare of rostral ca2-L | 0.403 | 0.526 | 0.097 | 0.001 |
| stratum lacunosum moleculare of rostral ca2-R | 0.045 | 0.832 | 0.055 | <0.001 |
| stratum lacunosum moleculare of rostral ca3-L | 0.297 | 0.586 | 0.085 | <0.001 |
| stratum lacunosum moleculare of rostral ca3-R | 0.120 | 0.730 | 0.064 | <0.001 |
| stratum lacunosum moleculare of uncal ca1-L | 0.127 | 0.722 | 0.065 | <0.001 |
| stratum lacunosum moleculare of uncal ca1-R | 0.242 | 0.623 | 0.078 | <0.001 |
| stratum lacunosum moleculare of uncal ca2-L | 3.705 | 0.055 | 0.485 | 0.006 |
| stratum lacunosum moleculare of uncal ca2-R | 0.370 | 0.543 | 0.093 | 0.001 |
| stratum lacunosum moleculare of uncal ca3-L | 1.440 | 0.231 | 0.224 | 0.002 |
| stratum lacunosum moleculare of uncal ca3-R | 0.303 | 0.582 | 0.085 | <0.001 |
| stratum lucidum of caudal ca3-L | 0.124 | 0.725 | 0.064 | <0.001 |
| stratum lucidum of caudal ca3-R | 1.132 | 0.288 | 0.186 | 0.002 |
| stratum lucidum of rostral ca3-L | 0.551 | 0.458 | 0.115 | 0.001 |
| stratum lucidum of rostral ca3-R | 0.030 | 0.863 | 0.053 | <0.001 |
| stratum lucidum of uncal ca3-L | 0.306 | 0.580 | 0.086 | <0.001 |
| stratum lucidum of uncal ca3-R | 0.727 | 0.394 | 0.136 | 0.001 |
| stratum oriens of caudal ca1-L | 0.129 | 0.720 | 0.065 | <0.001 |
| stratum oriens of caudal ca1-R | 0.749 | 0.387 | 0.139 | 0.001 |
| stratum oriens of caudal ca2-L | 1.213 | 0.271 | 0.196 | 0.002 |
| stratum oriens of caudal ca2-R | 1.177 | 0.278 | 0.191 | 0.002 |
| stratum oriens of caudal ca3-L | 0.097 | 0.755 | 0.061 | <0.001 |
| stratum oriens of caudal ca3-R | 0.407 | 0.524 | 0.098 | 0.001 |
| stratum oriens of rostral ca1-L | 0.368 | 0.544 | 0.093 | 0.001 |
| stratum oriens of rostral ca1-R | 1.111 | 0.292 | 0.183 | 0.002 |
| stratum oriens of rostral ca2-L | 0.332 | 0.565 | 0.089 | 0.001 |
| stratum oriens of rostral ca2-R | 0.550 | 0.459 | 0.115 | 0.001 |
| stratum oriens of rostral ca3-L | 0.463 | 0.496 | 0.104 | 0.001 |
| stratum oriens of rostral ca3-R | 0.033 | 0.856 | 0.054 | <0.001 |
| stratum oriens of uncal ca1-L | 0.453 | 0.501 | 0.103 | 0.001 |
| stratum oriens of uncal ca1-R | 0.084 | 0.772 | 0.060 | <0.001 |
| stratum oriens of uncal ca2-L | 0.033 | 0.856 | 0.054 | <0.001 |
| stratum oriens of uncal ca2-R | 0.309 | 0.579 | 0.086 | <0.001 |
| stratum oriens of uncal ca3-L | 0.025 | 0.874 | 0.053 | <0.001 |
| stratum oriens of uncal ca3-R | 0.014 | 0.906 | 0.052 | <0.001 |
| stratum pyramidale of caudal ca1-L | 0.009 | 0.926 | 0.051 | <0.001 |
| stratum pyramidale of caudal ca1-R | 1.409 | 0.236 | 0.220 | 0.002 |
| stratum pyramidale of caudal ca2-L | 0.812 | 0.368 | 0.147 | 0.001 |
| stratum pyramidale of caudal ca2-R | 1.322 | 0.251 | 0.209 | 0.002 |
| stratum pyramidale of caudal ca3-L | 0.086 | 0.769 | 0.060 | <0.001 |
| stratum pyramidale of caudal ca3-R | 0.839 | 0.360 | 0.150 | 0.001 |
| stratum pyramidale of rostral ca1-L | 0.018 | 0.892 | 0.052 | <0.001 |
| stratum pyramidale of rostral ca1-R | 1.242 | 0.266 | 0.199 | 0.002 |
| stratum pyramidale of rostral ca2-L | 0.709 | 0.400 | 0.134 | 0.001 |
| stratum pyramidale of rostral ca2-R | 0.120 | 0.729 | 0.064 | <0.001 |
| stratum pyramidale of rostral ca3-L | 0.785 | 0.376 | 0.143 | 0.001 |
| stratum pyramidale of rostral ca3-R | 0.094 | 0.759 | 0.061 | <0.001 |
| stratum pyramidale of uncal ca1-L | 1.201 | 0.274 | 0.194 | 0.002 |
| stratum pyramidale of uncal ca1-R | 0.848 | 0.358 | 0.151 | 0.001 |
| stratum pyramidale of uncal ca2-L | 0.494 | 0.482 | 0.108 | 0.001 |
| stratum pyramidale of uncal ca2-R | 1.160 | 0.282 | 0.189 | 0.002 |
| stratum pyramidale of uncal ca3-L | 0.012 | 0.914 | 0.051 | <0.001 |
| stratum pyramidale of uncal ca3-R | 0.008 | 0.929 | 0.051 | <0.001 |
| stratum radiatum of caudal ca1-L | 0.015 | 0.902 | 0.052 | <0.001 |
| stratum radiatum of caudal ca1-R | 2.091 | 0.149 | 0.303 | 0.003 |
| stratum radiatum of caudal ca2-L | 0.818 | 0.366 | 0.147 | 0.001 |
| stratum radiatum of caudal ca2-R | 1.041 | 0.308 | 0.175 | 0.002 |
| stratum radiatum of rostral ca1-L | 0.021 | 0.885 | 0.052 | <0.001 |
| stratum radiatum of rostral ca1-R | 0.608 | 0.436 | 0.122 | 0.001 |
| stratum radiatum of rostral ca2-L | 0.278 | 0.598 | 0.082 | <0.001 |
| stratum radiatum of rostral ca2-R | 0.001 | 0.975 | 0.050 | <0.001 |
| stratum radiatum of uncal ca1-L | 1.655 | 0.199 | 0.250 | 0.003 |
| stratum radiatum of uncal ca1-R | 0.889 | 0.346 | 0.156 | 0.001 |
| stratum radiatum of uncal ca2-L | 0.438 | 0.509 | 0.101 | 0.001 |
| stratum radiatum of uncal ca2-R | 0.486 | 0.486 | 0.107 | 0.001 |
| stria medullaris of thalamus-L | 3.886 | 0.049 | 0.503 | 0.006 |
| stria medullaris of thalamus-R | 0.001 | 0.976 | 0.050 | <0.001 |
| stria terminalis-L | 0.012 | 0.914 | 0.051 | <0.001 |
| stria terminalis-R | 0.005 | 0.942 | 0.051 | <0.001 |
| subparafascicular nucleus of thalamus-L | 0.049 | 0.825 | 0.056 | <0.001 |
| subparafascicular nucleus of thalamus-R | 0.010 | 0.919 | 0.051 | <0.001 |
| substantia innominata-L | 1.636 | 0.201 | 0.248 | 0.003 |
| substantia innominata-R | 2.675 | 0.102 | 0.372 | 0.004 |
| substantia nigra compact part-L | 2.131 | 0.145 | 0.308 | 0.003 |
| substantia nigra compact part-R | 0.965 | 0.326 | 0.165 | 0.002 |
| substantia nigra reticular part-L | 1.631 | 0.202 | 0.247 | 0.003 |
| substantia nigra reticular part-R | 0.624 | 0.430 | 0.124 | 0.001 |
| superficial pulvinar nucleus-L | 0.145 | 0.704 | 0.067 | <0.001 |
| superficial pulvinar nucleus-R | 0.379 | 0.538 | 0.094 | 0.001 |
| superior cerebellar peduncle brachium conjunctivum-L | 2.307 | 0.129 | 0.329 | 0.004 |
| superior cerebellar peduncle brachium conjunctivum-R | 0.691 | 0.406 | 0.132 | 0.001 |
| suprachiasmatic nucleus-L | 1.195 | 0.275 | 0.194 | 0.002 |
| suprachiasmatic nucleus-R | 0.325 | 0.569 | 0.088 | 0.001 |
| suprageniculate nucleus of thalamus-L | 0.529 | 0.467 | 0.112 | 0.001 |
| suprageniculate nucleus of thalamus-R | 1.567 | 0.211 | 0.240 | 0.002 |
| supramammillary nucleus-L | 0.058 | 0.810 | 0.057 | <0.001 |
| supramammillary nucleus-R | 0.999 | 0.318 | 0.170 | 0.002 |
| supraoptic dicussation-L | 0.083 | 0.774 | 0.059 | <0.001 |
| supraoptic dicussation-R | 1.754 | 0.186 | 0.262 | 0.003 |
| supraoptic nucleus-L | 0.388 | 0.534 | 0.095 | 0.001 |
| supraoptic nucleus-R | 0.591 | 0.442 | 0.120 | 0.001 |
| supraoptic region of hth-L | 0.335 | 0.563 | 0.089 | 0.001 |
| supraoptic region of hth-R | 0.980 | 0.322 | 0.167 | 0.002 |
| tail of caudate-L | 0.066 | 0.797 | 0.058 | <0.001 |
| tail of caudate-R | 0.026 | 0.872 | 0.053 | <0.001 |
| temporal claustrum-L | 0.004 | 0.947 | 0.051 | <0.001 |
| temporal claustrum-R | 0.484 | 0.487 | 0.107 | 0.001 |
| thalamic fasciculus-L | 0.207 | 0.650 | 0.074 | <0.001 |
| thalamic fasciculus-R | 0.192 | 0.662 | 0.072 | <0.001 |
| thalamus-L | 3.132 | 0.077 | 0.424 | 0.005 |
| thalamus-R | 3.313 | 0.069 | 0.443 | 0.005 |
| tuberomammillary nucleus-L | 1.602 | 0.206 | 0.244 | 0.003 |
| tuberomammillary nucleus-R | 0.323 | 0.570 | 0.088 | 0.001 |
| uncal subiculum-L | 0.746 | 0.388 | 0.139 | 0.001 |
| uncal subiculum-R | 3.301 | 0.070 | 0.442 | 0.005 |
| ventral division of basomedial nucleus-L | 1.281 | 0.258 | 0.204 | 0.002 |
| ventral division of basomedial nucleus-R | <0.001 | 0.995 | 0.050 | <0.001 |
| ventral medial nucleus of thalamus-L | 0.262 | 0.609 | 0.080 | <0.001 |
| ventral medial nucleus of thalamus-R | 1.015 | 0.314 | 0.172 | 0.002 |
| ventral pallidus-L | 0.492 | 0.483 | 0.108 | 0.001 |
| ventral pallidus-R | 0.202 | 0.653 | 0.073 | <0.001 |
| ventral part of ventromedial hypothalamic nucleus-L | 0.789 | 0.375 | 0.144 | 0.001 |
| ventral part of ventromedial hypothalamic nucleus-R | 1.918 | 0.167 | 0.282 | 0.003 |
| ventral posterior inferior nucleus-L | 1.300 | 0.255 | 0.207 | 0.002 |
| ventral posterior inferior nucleus-R | 1.531 | 0.216 | 0.235 | 0.002 |
| ventral posterior medial nucleus-L | 0.253 | 0.615 | 0.079 | <0.001 |
| ventral posterior medial nucleus-R | 0.227 | 0.634 | 0.076 | <0.001 |
| ventral subdivision of coa-L | 0.475 | 0.491 | 0.106 | 0.001 |
| ventral subdivision of coa-R | 0.704 | 0.402 | 0.134 | 0.001 |
| ventral subdivision of vlc-L | 1.111 | 0.292 | 0.183 | 0.002 |
| ventral subdivision of vlc-R | 0.526 | 0.469 | 0.112 | 0.001 |
| ventral tegmental area-L | 2.406 | 0.121 | 0.341 | 0.004 |
| ventral tegmental area-R | 1.073 | 0.301 | 0.179 | 0.002 |
| white matter of forebrain-L | 1.819 | 0.178 | 0.270 | 0.003 |
| white matter of forebrain-R | 3.133 | 0.077 | 0.424 | 0.005 |
| white matter of hindbrain-L | 2.006 | 0.157 | 0.293 | 0.003 |
| white matter of hindbrain-R | 1.540 | 0.215 | 0.236 | 0.002 |
| white matter of the cerebellum-L | 3.711 | 0.055 | 0.485 | 0.006 |
| white matter of the cerebellum-R | 4.472 | 0.035 | 0.560 | 0.007 |
| zona incerta-L | 0.117 | 0.732 | 0.063 | <0.001 |
| zona incerta-R | 1.021 | 0.313 | 0.172 | 0.002 |

*Abbreviations:* COA=Cortical amygdaloid area; MD=Mediodorsal nucleus of the thalamus; PVA=Paraventricular Anterior nucleus; sth=Subthalamic nucleus; VA=Ventroanterior nucleus of thalamus; VL=Ventrolateral nucleus of thalamus; VLC=Ventral Lateral nucleus. Pars caudalis of the thalamus; VPM=Ventral Posteromedial nucleus of the thalamus.

**Table S6. Descriptive statistics for the exploratory cortical volumetric analysis using the HistoAtlas integrated within NextBrain.**

| Structure | Group | N | Mean  (mm³) | SD (mm³) | SE (mm³) |
| --- | --- | --- | --- | --- | --- |
| bankssts-L | PD-mVHs⁻ | 577 | 2981 | 698 | 29 |
|  | PD-mVHs^+^ | 64 | 2863 | 783 | 98 |
| bankssts-R | PD-mVHs⁻ | 577 | 3453 | 760 | 32 |
|  | PD-mVHs^+^ | 64 | 3437 | 845 | 106 |
| caudal anterior cingulate-L | PD-mVHs⁻ | 577 | 2688 | 588 | 24 |
|  | PD-mVHs^+^ | 64 | 2666 | 532 | 66 |
| caudal anterior cingulate-R | PD-mVHs⁻ | 577 | 2997 | 637 | 27 |
|  | PD-mVHs^+^ | 64 | 2833 | 472 | 59 |
| caudal middle frontal-L | PD-mVHs⁻ | 577 | 6704 | 1145 | 48 |
|  | PD-mVHs^+^ | 64 | 6673 | 1162 | 145 |
| caudal middle frontal-R | PD-mVHs⁻ | 577 | 6622 | 1207 | 50 |
|  | PD-mVHs^+^ | 64 | 6626 | 1187 | 148 |
| cuneus-L | PD-mVHs⁻ | 577 | 2852 | 659 | 27 |
|  | PD-mVHs^+^ | 64 | 2737 | 504 | 63 |
| cuneus-R | PD-mVHs⁻ | 577 | 2949 | 622 | 26 |
|  | PD-mVHs^+^ | 64 | 2835 | 533 | 67 |
| entorhinal-L | PD-mVHs⁻ | 577 | 2511 | 599 | 25 |
|  | PD-mVHs^+^ | 64 | 2598 | 644 | 80 |
| entorhinal-R | PD-mVHs⁻ | 577 | 2537 | 556 | 23 |
|  | PD-mVHs^+^ | 64 | 2623 | 601 | 75 |
| frontal pole-L | PD-mVHs⁻ | 577 | 1222 | 295 | 12 |
|  | PD-mVHs^+^ | 64 | 1177 | 281 | 35 |
| frontal pole-R | PD-mVHs⁻ | 577 | 1293 | 301 | 13 |
|  | PD-mVHs^+^ | 64 | 1296 | 332 | 42 |
| fusiform-L | PD-mVHs⁻ | 577 | 13939 | 1790 | 75 |
|  | PD-mVHs^+^ | 64 | 13484 | 1531 | 191 |
| fusiform-R | PD-mVHs⁻ | 577 | 13475 | 1832 | 76 |
|  | PD-mVHs^+^ | 64 | 13166 | 1481 | 185 |
| inferior parietal-L | PD-mVHs⁻ | 577 | 14594 | 1969 | 82 |
|  | PD-mVHs^+^ | 64 | 14273 | 1767 | 221 |
| inferior parietal-R | PD-mVHs⁻ | 577 | 15505 | 2145 | 89 |
|  | PD-mVHs^+^ | 64 | 15006 | 1879 | 235 |
| inferior temporal-L | PD-mVHs⁻ | 577 | 12713 | 1785 | 74 |
|  | PD-mVHs^+^ | 64 | 12436 | 1632 | 204 |
| inferior temporal-R | PD-mVHs⁻ | 577 | 12409 | 1743 | 73 |
|  | PD-mVHs^+^ | 64 | 12238 | 1629 | 204 |
| insul-L | PD-mVHs⁻ | 577 | 7380 | 880 | 37 |
|  | PD-mVHs^+^ | 64 | 7372 | 905 | 113 |
| insula-R | PD-mVHs⁻ | 577 | 7038 | 841 | 35 |
|  | PD-mVHs^+^ | 64 | 7027 | 825 | 103 |
| Isthmuscingulate-L | PD-mVHs⁻ | 577 | 3284 | 595 | 25 |
|  | PD-mVHs^+^ | 64 | 3352 | 568 | 71 |
| isthmuscingulate-R | PD-mVHs⁻ | 577 | 2996 | 529 | 22 |
|  | PD-mVHs^+^ | 64 | 2944 | 492 | 62 |
| lateral occipital-L | PD-mVHs⁻ | 577 | 12723 | 2104 | 88 |
|  | PD-mVHs^+^ | 64 | 12968 | 1945 | 243 |
| lateral occipital-R | PD-mVHs⁻ | 577 | 13153 | 2121 | 88 |
|  | PD-mVHs^+^ | 64 | 12918 | 2068 | 258 |
| lateral orbito-frontal-L | PD-mVHs⁻ | 577 | 9649 | 1163 | 48 |
|  | PD-mVHs^+^ | 64 | 9589 | 1145 | 143 |
| lateral orbito-frontal-R | PD-mVHs⁻ | 577 | 9471 | 1155 | 48 |
|  | PD-mVHs^+^ | 64 | 9362 | 1067 | 133 |
| lingual-L | PD-mVHs⁻ | 577 | 8446 | 1258 | 52 |
|  | PD-mVHs^+^ | 64 | 8301 | 1375 | 172 |
| lingual-R | PD-mVHs⁻ | 577 | 7863 | 1065 | 44 |
|  | PD-mVHs^+^ | 64 | 7681 | 1085 | 136 |
| medial orbito-frontal-L | PD-mVHs⁻ | 577 | 6987 | 1067 | 44 |
|  | PD-mVHs^+^ | 64 | 6770 | 1043 | 130 |
| medial orbito-frontal-R | PD-mVHs⁻ | 577 | 7525 | 1121 | 47 |
|  | PD-mVHs^+^ | 64 | 7305 | 1130 | 141 |
| middle temporal-L | PD-mVHs⁻ | 577 | 12831 | 1966 | 82 |
|  | PD-mVHs^+^ | 64 | 12460 | 1993 | 249 |
| Middle temporal-R | PD-mVHs⁻ | 577 | 13203 | 1917 | 80 |
|  | PD-mVHs^+^ | 64 | 12826 | 1907 | 238 |
| paracentral-L | PD-mVHs⁻ | 577 | 4272 | 723 | 30 |
|  | PD-mVHs^+^ | 64 | 4299 | 807 | 101 |
| paracentral-R | PD-mVHs⁻ | 577 | 4548 | 824 | 34 |
|  | PD-mVHs^+^ | 64 | 4505 | 847 | 106 |
| parahippocampal-L | PD-mVHs⁻ | 577 | 2880 | 359 | 15 |
|  | PD-mVHs^+^ | 64 | 2832 | 333 | 42 |
| parahippocampal-R | PD-mVHs⁻ | 577 | 2753 | 339 | 14 |
|  | PD-mVHs^+^ | 64 | 2720 | 332 | 41 |
| pars opercularis-L | PD-mVHs⁻ | 577 | 3999 | 714 | 30 |
|  | PD-mVHs^+^ | 64 | 4050 | 681 | 85 |
| pars opercularis-R | PD-mVHs⁻ | 577 | 4040 | 712 | 30 |
|  | PD-mVHs^+^ | 64 | 3976 | 693 | 87 |
| pars orbitalis-L | PD-mVHs⁻ | 577 | 2666 | 585 | 24 |
|  | PD-mVHs^+^ | 64 | 2624 | 473 | 59 |
| pars orbitalis-R | PD-mVHs⁻ | 577 | 2626 | 562 | 23 |
|  | PD-mVHs^+^ | 64 | 2582 | 509 | 64 |
| pars triangularis-L | PD-mVHs⁻ | 577 | 3434 | 734 | 31 |
|  | PD-mVHs^+^ | 64 | 3315 | 728 | 91 |
| pars triangularis-R | PD-mVHs⁻ | 577 | 3298 | 734 | 31 |
|  | PD-mVHs^+^ | 64 | 3264 | 759 | 95 |
| pericalcarine-L | PD-mVHs⁻ | 577 | 3612 | 752 | 31 |
|  | PD-mVHs^+^ | 64 | 3600 | 721 | 90 |
| pericalcarine-R | PD-mVHs⁻ | 577 | 3404 | 642 | 27 |
|  | PD-mVHs^+^ | 64 | 3397 | 706 | 88 |
| post central-L | PD-mVHs⁻ | 577 | 11178 | 1566 | 65 |
|  | PD-mVHs^+^ | 64 | 10975 | 1580 | 197 |
| post central-R | PD-mVHs⁻ | 577 | 10261 | 1337 | 56 |
|  | PD-mVHs^+^ | 64 | 9981 | 1142 | 143 |
| posterior cingulate-L | PD-mVHs⁻ | 577 | 3433 | 679 | 28 |
|  | PD-mVHs^+^ | 64 | 3293 | 582 | 73 |
| posterior cingulate-R | PD-mVHs⁻ | 577 | 3443 | 648 | 27 |
|  | PD-mVHs^+^ | 64 | 3407 | 552 | 69 |
| precentral-L | PD-mVHs⁻ | 577 | 14807 | 1853 | 77 |
|  | PD-mVHs^+^ | 64 | 14840 | 1866 | 233 |
| precentral-R | PD-mVHs⁻ | 577 | 15186 | 1947 | 81 |
|  | PD-mVHs^+^ | 64 | 14960 | 1939 | 242 |
| precuneus-L | PD-mVHs⁻ | 577 | 9937 | 1735 | 72 |
|  | PD-mVHs^+^ | 64 | 9752 | 1734 | 217 |
| precuneus-R | PD-mVHs⁻ | 577 | 10445 | 1702 | 71 |
|  | PD-mVHs^+^ | 64 | 10339 | 1627 | 203 |
| rostral anterior cingulate-L | PD-mVHs⁻ | 577 | 2819 | 658 | 27 |
|  | PD-mVHs^+^ | 64 | 2764 | 651 | 81 |
| rostral anterior cingulate-R | PD-mVHs⁻ | 577 | 2664 | 597 | 25 |
|  | PD-mVHs^+^ | 64 | 2596 | 547 | 68 |
| rostral middle frontal-L | PD-mVHs⁻ | 577 | 18508 | 2470 | 103 |
|  | PD-mVHs^+^ | 64 | 18384 | 2755 | 344 |
| rostral middle frontal-R | PD-mVHs⁻ | 577 | 18387 | 2337 | 97 |
|  | PD-mVHs^+^ | 64 | 18222 | 2502 | 313 |
| superior frontal-L | PD-mVHs⁻ | 577 | 22237 | 2932 | 122 |
|  | PD-mVHs^+^ | 64 | 21960 | 3275 | 409 |
| superior frontal-R | PD-mVHs⁻ | 577 | 21915 | 2996 | 125 |
|  | PD-mVHs^+^ | 64 | 22218 | 2965 | 371 |
| superior parietal-L | PD-mVHs⁻ | 577 | 13656 | 1819 | 76 |
|  | PD-mVHs^+^ | 64 | 13587 | 1982 | 248 |
| superior parietal-R | PD-mVHs⁻ | 577 | 13966 | 1961 | 82 |
|  | PD-mVHs^+^ | 64 | 13724 | 1834 | 229 |
| superior temporal-L | PD-mVHs⁻ | 577 | 12327 | 1756 | 73 |
|  | PD-mVHs^+^ | 64 | 12174 | 1809 | 226 |
| superior temporal-R | PD-mVHs⁻ | 577 | 12228 | 1523 | 63 |
|  | PD-mVHs^+^ | 64 | 12124 | 1596 | 199 |
| supramarginal-L | PD-mVHs⁻ | 577 | 11840 | 1849 | 77 |
|  | PD-mVHs^+^ | 64 | 11986 | 1972 | 246 |
| supramarginal-R | PD-mVHs⁻ | 577 | 10905 | 1711 | 71 |
|  | PD-mVHs^+^ | 64 | 10975 | 1821 | 228 |
| temporal pole-L | PD-mVHs⁻ | 577 | 2841 | 458 | 19 |
|  | PD-mVHs^+^ | 64 | 2868 | 427 | 53 |
| temporal pole-R | PD-mVHs⁻ | 577 | 3041 | 446 | 19 |
|  | PD-mVHs^+^ | 64 | 3026 | 404 | 51 |
| transverse temporal-L | PD-mVHs⁻ | 577 | 1106 | 246 | 10 |
|  | PD-mVHs^+^ | 64 | 1103 | 245 | 31 |
| transverse temporal-R | PD-mVHs⁻ | 577 | 941 | 214 | 9 |
|  | PD-mVHs^+^ | 64 | 935 | 228 | 28 |

*Abbreviations:* L=left; R=right

**Table S7. ANCOVA outputs for the exploratory cortical volumetric analysis using the HistoAtlas integrated within NextBrain.** ANCOVA models included group and sex as fixed factors, together with the group × sex interaction, and age, education, standardized eTIV, MoCA score, and disease duration as continuous covariates.

| Structure | F | p-value | Observed power (alpha=0.05) | Partial eta squared |
| --- | --- | --- | --- | --- |
| bankssts-L | 3.126 | 0.078 | 0.423 | 0.005 |
| bankssts-R | 0.238 | 0.626 | 0.078 | 0.000 |
| caudal anterior cingulate-L | 0.025 | 0.874 | 0.053 | 0.000 |
| caudal anterior cingulate-R | 3.175 | 0.075 | 0.428 | 0.005 |
| caudal middle frontal-L | 0.406 | 0.524 | 0.098 | 0.001 |
| caudal middle frontal-R | 0.013 | 0.908 | 0.052 | 0.000 |
| cuneus-L | 2.704 | 0.101 | 0.375 | 0.004 |
| cuneus-R | 2.733 | 0.099 | 0.379 | 0.004 |
| entorhinal-L | 1.086 | 0.298 | 0.180 | 0.002 |
| entorhinal-R | 0.233 | 0.630 | 0.077 | 0.000 |
| frontalpole-L | 0.367 | 0.545 | 0.093 | 0.001 |
| frontalpole-R | 0.092 | 0.762 | 0.061 | 0.000 |
| fusiform-L | 3.511 | 0.061 | 0.465 | 0.006 |
| fusiform-R | 3.147 | 0.077 | 0.425 | 0.005 |
| inferior parietal-L | 2.376 | 0.124 | 0.337 | 0.004 |
| inferior parietal-R | 5.945 | 0.015 | 0.682 | 0.009 |
| inferior temporal-L | 2.477 | 0.116 | 0.349 | 0.004 |
| inferior temporal-R | 2.546 | 0.111 | 0.357 | 0.004 |
| insula-L | 0.241 | 0.624 | 0.078 | 0.000 |
| insula-R | 0.174 | 0.677 | 0.070 | 0.000 |
| isthmus cingulate-L | 0.302 | 0.583 | 0.085 | 0.000 |
| isthmus cingulate-R | 0.344 | 0.557 | 0.090 | 0.001 |
| lateral occipital-L | 0.240 | 0.624 | 0.078 | 0.000 |
| lateral occipital-R | 2.155 | 0.143 | 0.311 | 0.003 |
| lateral orbitofrontal-L | 0.500 | 0.480 | 0.109 | 0.001 |
| lateral orbitofrontal-R | 0.789 | 0.375 | 0.144 | 0.001 |
| lingual-L | 3.623 | 0.057 | 0.476 | 0.006 |
| lingual-R | 4.050 | 0.045 | 0.520 | 0.006 |
| medial orbitofrontal-L | 2.239 | 0.135 | 0.321 | 0.004 |
| medial orbitofrontal-R | 3.864 | 0.050 | 0.501 | 0.006 |
| middle temporal-L | 4.918 | 0.027 | 0.600 | 0.008 |
| middle temporal-R | 4.076 | 0.044 | 0.522 | 0.006 |
| para-central-L | 0.063 | 0.802 | 0.057 | 0.000 |
| para-central-R | 1.247 | 0.265 | 0.200 | 0.002 |
| parahippocampal-L | 0.544 | 0.461 | 0.114 | 0.001 |
| parahippocampal-R | 0.425 | 0.515 | 0.100 | 0.001 |
| pars-opercularis-L | 0.086 | 0.770 | 0.060 | 0.000 |
| pars-opercularis-R | 1.151 | 0.284 | 0.188 | 0.002 |
| pars-orbitalis-L | 0.969 | 0.325 | 0.166 | 0.002 |
| pars-orbitalis-R | 0.468 | 0.494 | 0.105 | 0.001 |
| pars-triangularis-L | 1.499 | 0.221 | 0.231 | 0.002 |
| pars-triangularis-R | 1.249 | 0.264 | 0.200 | 0.002 |
| pericalcarine-L | 2.184 | 0.140 | 0.314 | 0.003 |
| pericalcarine-R | 0.667 | 0.414 | 0.129 | 0.001 |
| postcentral-L | 1.481 | 0.224 | 0.229 | 0.002 |
| postcentral-R | 2.715 | 0.100 | 0.377 | 0.004 |
| posterior cingulate-L | 1.968 | 0.161 | 0.288 | 0.003 |
| posterior cingulate-R | 0.180 | 0.671 | 0.071 | 0.000 |
| precentral-L | 0.218 | 0.641 | 0.075 | 0.000 |
| precentral-R | 3.102 | 0.079 | 0.420 | 0.005 |
| precuneus-L | 1.264 | 0.261 | 0.202 | 0.002 |
| precuneus-R | 1.340 | 0.248 | 0.212 | 0.002 |
| rostral anterior-cingulate-L | 1.224 | 0.269 | 0.197 | 0.002 |
| rostral anterior-cingulate-R | 0.716 | 0.398 | 0.135 | 0.001 |
| rostral middle-frontal-L | 0.582 | 0.446 | 0.119 | 0.001 |
| rostral middle-frontal-R | 0.805 | 0.370 | 0.146 | 0.001 |
| superior frontal-L | 1.722 | 0.190 | 0.259 | 0.003 |
| superior frontal-R | 0.448 | 0.504 | 0.103 | 0.001 |
| superior parietal-L | 0.591 | 0.442 | 0.120 | 0.001 |
| superiorparietal-R | 0.492 | 0.483 | 0.108 | 0.001 |
| superior temporal-L | 0.177 | 0.674 | 0.070 | 0.000 |
| superior temporal-R | 0.129 | 0.719 | 0.065 | 0.000 |
| supramarginal-L | 0.049 | 0.825 | 0.056 | 0.000 |
| supramarginal-R | 0.023 | 0.880 | 0.053 | 0.000 |
| temporal pole-L | 0.057 | 0.811 | 0.057 | 0.000 |
| temporal pole-R | 0.936 | 0.334 | 0.162 | 0.001 |
| transverse temporal-L | 0.071 | 0.790 | 0.058 | 0.000 |
| transverse temporal-R | 0.103 | 0.749 | 0.062 | 0.000 |

*Abbreviations:* L=left; R=right

**Table S8. Logistic regression model including selected clinical and neuropsychological predictors of hallucination-related status.** The model included HVLT-IR, SF-Animal, GDS, and disease duration. Higher HVLT-IR, GDS, and disease duration scores were associated with greater odds of hallucination-related status, whereas higher SF-Animal scores were associated with lower odds.

| Variable | B | S.E. | Wald | p-value | Exp(B) |
| --- | --- | --- | --- | --- | --- |
| HVLT-IR | **0.092** | **0.031** | **8.946** | **0.003** | **1.097** |
| **SF-Animal** | **−0.094** | **0.028** | **11.457** | **0.001** | **0.911** |
| GDS | **0.261** | **0.083** | **10.002** | **0.002** | 1.298 |
| Disease duration (years) | **0.254** | **0.076** | **11.109** | **0.001** | **1.289** |
| Constant | **−4.377** | **0.976** | **20.125** | <0.001 | **0.013** |

−2 Log Likelihood (−2LL) = 380.225; Nagelkerke’s R² = 0.115; Cox & Snell R² = 0.055.

HVLT-IR, GDS, disease duration, and SF-Animal were significantly associated with hallucination-related status. Higher HVLT-IR, GDS, and disease duration scores were associated with greater odds, whereas higher SF-Animal scores were associated with lower odds.

*Abbreviations:* B = regression coefficient; GDS = Geriatric Depression Scale; HVLT-IR = Hopkins Verbal Learning Test-Revised – Immediate Recall; SE = standard error; SF-Animal = Semantic Fluency – Total Number of Animals; Exp(B) = odds ratio.

**Table S9. Bootstrap-estimated regression coefficients for the model including selected clinical and neuropsychological predictors of hallucination-related status.** Bootstrap regression coefficients and BCa 95% confidence intervals are reported for the clinical/neuropsychological model. Bootstrap analyses supported the associations of HVLT-IR, SF-Animal, GDS, and disease duration, with all BCa 95% confidence intervals excluding zero.

| Variable | B | Bias | S.E. | p-value | BCa 95% CI lower | BCa 95% CI upper |
| --- | --- | --- | --- | --- | --- | --- |
| HVLT-IR | **0.092** | **0.001** | **0.031** | **0.003** | **0.032** | **0.159** |
| **SF-Animal** | **−0.094** | **−0.002** | **0.029** | **0.002** | **−0.151** | **−0.044** |
| GDS | **0.261** | **0.004** | **0.092** | **0.002** | **0.061** | **0.454** |
| Disease duration (years) | **0.254** | **0.005** | **0.089** | **0.002** | **0.071** | **0.446** |
| Constant | **−4.377** | **−0.050** | **0.975** | <0.001 | **−6.256** | **−2.632** |

**The bootstrap analysis supported positive associations of HVLT-IR, GDS, and disease duration and an inverse association of SF-Animal with hallucination-related status. BCa 95% confidence intervals excluded zero for all four predictors, supporting the stability of their coefficients.**

***Abbreviations:* B = regression coefficient; BCa = bias-corrected and accelerated; GDS = Geriatric Depression Scale; HVLT-IR = Hopkins Verbal Learning Test-Revised – Immediate Recall;** S.E. = bootstrap standard error; **SF-Animal = Semantic Fluency – Total Number of Animals.**
